# FAM134B Restricts African Swine Fever Virus Capsid Assembly via Reticulophagy and Its Antiviral Activity is Antagonized by the Viral Virulence‐Associated Factor pMGF300‐2R

**DOI:** 10.1002/advs.77741

**Published:** 2026-09-16

**Authors:** Rui Luo, Jing Zhang, Ruojia Huang, Xianfeng Zhang, Lian‐Feng Li, Jing Lan, Zhanhao Lu, Yuan Sun, Tao Wang, Hua‐Ji Qiu

**Affiliations:** ^1^ State Key Laboratory of Animal Disease Control and Prevention Professional Laboratory for African Swine Fever (Harbin), National High Containment Facilities for Animal Disease Control and Prevention, Harbin Veterinary Research Institute Chinese Academy of Agricultural Sciences Harbin China

**Keywords:** African swine fever virus, FAM134B, LC3‐interacting region (LIR), p72, pA137R, pMGF300‐2R, reticulophagy

## Abstract

African swine fever (ASF), caused by African swine fever virus (ASFV), is a devastating viral disease in domestic pigs and wild boar. ASFV is a large DNA virus whose capsid assembly takes place within perinuclear viral factories (VFs). Accumulating evidence has demonstrated that reticulophagy confers antiviral activity against multiple RNA viruses. Nevertheless, the roles of reticulophagy in DNA virus replication and viral immunoevasion mechanisms remain largely uncharacterized. Here, we revealed that FAM134B, a reticulophagy receptor, markedly inhibits the maturation of ASFV in VFs. Mechanistically, FAM134B interacts with the major viral capsid proteins p72 and pA137R, facilitating their delivery to lysosomes for reticulophagy‐mediated degradation and thus blocking ASFV capsid assembly. Additionally, we found that ASFV exploits its virulence‐associated factor pMGF300‐2R to target FAM134B and enhance viral replication. Further investigations revealed that pMGF300‐2R binds to FAM134B and triggers its autophagic degradation via the C‐terminal LC3‐interacting region (LIR) motif. Importantly, the *MGF300‐2R*‐deleted ASFV mutant exhibited significantly reduced replication and virulence compared with wild‐type ASFV in pigs, which was attributed to increased FAM134B expression. Collectively, this study establishes the first mechanistic link between reticulophagy and DNA virus replication and underscores the critical role of FAM134B‐mediated reticulophagy in host defense against ASFV infection.

## 1 Introduction

The endoplasmic reticulum (ER) serves as a central hub for viral replication and propagation, rendering it a primary target of host defense mechanisms. Reticulophagy, a selective autophagy pathway, specifically mediates the degradation of ER subdomains [1]. Reticulophagy is mediated by selective autophagy receptors (SARs), including FAM134B, SEC62, RTN3, CCPG1, and ATL3. These receptors selectively recognize misfolded and unfolded proteins in the ER and facilitate their trafficking to lysosomes for degradation via the evolutionarily conserved LC3‐interacting region (LIR), thus maintaining cellular homeostasis [2, 3, 4, 5, 6]. A wide range of pathogens have evolved diverse strategies to evade host immune surveillance by hijacking reticulophagy machinery and inhibiting the functions of SARs [7]. For example, flaviviruses including dengue virus, Zika virus (ZIKV), and West Nile virus, encode proteases that cleave FAM134B to promote viral replication [8]. Recent research has found that the coronavirus subverts reticulophagy by hijacking FAM134B and ATL3 into p62‐containing condensates, thereby enhancing viral replication [9]. Furthermore, ZIKV exemplifies this hijacking mechanism by promoting the AMFR‐mediated K48‐linked polyubiquitination of the viral NS2A protein, which triggers FAM134B degradation, thereby reducing ER turnover and ultimately enhancing viral replication and pathogenicity [10]. The engagement of reticulophagy in RNA viral infections has been extensively studied [11]. However, its function in the clearance of DNA viruses remains largely unexplored.

African swine fever virus (ASFV) is a major animal pathogen [12]. As an enveloped DNA virus, ASFV possesses a genome of approximately 194 kilobase pairs (kb) and encodes roughly 200 viral proteins, many of which disrupt the host's innate antiviral immune response by manipulating the autophagy pathway [13, 14]. ASFV is the etiological agent of African swine fever (ASF), a highly hemorrhagic and often lethal disease affecting both domestic pigs and wild boar [15]. ASF causes severe socioeconomic losses and poses a major threat to global food security. According to the World Organisation for Animal Health (WOAH) African swine fever (ASF)—Situation Report 78 (https://www.woah.org/en/document/african‐swine‐fever‐asf‐ situation‐reports‐78/), ASF outbreaks since 2022 have caused more than 1,194,000 reported cases in domestic pigs, over 54,000 reported cases in wild boar, and the deaths of more than 2.67 million pigs and wild boar. Currently, no licensed vaccines or antiviral therapeutics are available to curb the global spread of ASF, underscoring an urgent demand to elucidate the molecular mechanism underlying ASFV infection [16].

The ASFV virion comprises five concentric layers: an outer envelope, a capsid, an inner membrane, a core shell, and a nucleoid enclosing the viral genome [17, 18, 19]. ASFV capsid assembly and viral particle maturation occur within specialized viral factories (VFs). Although three recent studies have elucidated the cryo‐electron microscopy structures of the intact ASFV virion [17, 18, 19], the regulatory mechanisms governing capsid assembly remain poorly understood. The ASFV capsid consists mainly of seven structural proteins: p72, pA137R, pH240R, pE120R, pM1249L, pB438L, and pD117L. As the major capsid protein, p72 is the most abundant structural component, accounting for approximately 30% of the total virion mass and acting as a major viral antigen. Notably, pA137R, pH240R, and pE120R have been characterized as critical virulence‐associated factors [20, 21, 22, 23]. Specifically, pA137R, pH240R, and pE120R inhibit the NF‐*κ*B and interferon signaling pathways, thereby suppressing innate immunity. ASFV mutants lacking any of these genes (*A137R*, *H240R*, or *E120R*) show potential as live attenuated vaccine candidates, providing complete protection against virulent ASFV challenge [20, 22, 23, 24]. Beyond their structural and immunomodulatory functions, little is known regarding the turnover of these capsid proteins. Whether reticulophagy targets ASFV capsid components and limits viral replication is an intriguing yet unanswered question.

Here, we demonstrated that FAM134B‐mediated reticulophagy degrades p72 and pA137R of ASFV, thereby inhibiting capsid assembly in VFs. In response, the MGF300‐2R protein (pMGF300‐2R), a virulence‐associated factor of ASFV, degrades FAM134B via autophagy, thus antagonizing its antiviral activity. Furthermore, we found that the degradation of FAM134B by pMGF300‐2R depends on the interaction between the LIR3 motif of pMGF300‐2R and LC3B. Notably, using wild‐type ASFV (ASFV‐WT) and the *MGF300‐2R*‐deleted mutant ASFV, we demonstrate that the pMGF300‐2R‐mediated autophagic degradation of FAM134B enhances ASFV infectivity and pathogenicity in vivo. This study elucidates that the FAM134B‐mediated reticulophagy exerts an anti‐ASFV function, emphasizing that ASFV hijacks the autophagy machinery through mimicking a host short linear protein‐protein interaction motif to facilitate efficient infection. Our findings uncover a previously unrecognized mechanism, with important implications for the development of antiviral strategies against ASFV.

## 2 Results

### 2.1 ASFV Infection Activates Reticulophagy

To investigate the effects of ASFV infection on reticulophagy, we constructed a reticulophagy reporter pssRFP‐GFP‐KDEL (Figure 1A), as described previously [25]. This reporter comprises an N‐terminal ER signal sequence (SS), a tandem cassette of monomeric red fluorescent protein (RFP) and green fluorescent protein (GFP), and a C‐terminal ER retention motif KDEL. Upon reticulophagy induction, this reporter is encapsulated within autophagosomes. Following autophagosome‐lysosome fusion, the acid‐labile GFP is quenched and degraded, whereas RFP remains stable in the acidic and proteolytic lysosomal lumen (Figure 1A). Reticulophagy can thus be monitored by Western blotting (to detect the ∼25‐kDa free RFP fragment) and laser confocal microscopy. Using pssRFP‐GFP‐KDEL, we first validated that amino acid starvation and EBSS treatment (a well‐established reticulophagy inducer) markedly increased the level of free RFP fragment in HEK293T cells, confirming the reliability of our reporter system (Figure 1B,C). We subsequently found that infection with either the HEK293T cell‐adapted ASFV strain (ASFV‐P31) or ASFV‐WT significantly upregulated the free RFP fragment levels in HEK293T and wild boar lung (WSL) cells, respectively, and induced abundant red puncta formation, consistent with the starvation‐induced reticulophagy activation (Figure 1B−E). Collectively, these results demonstrate that ASFV infection triggers reticulophagy activation.

**FIGURE 1:**
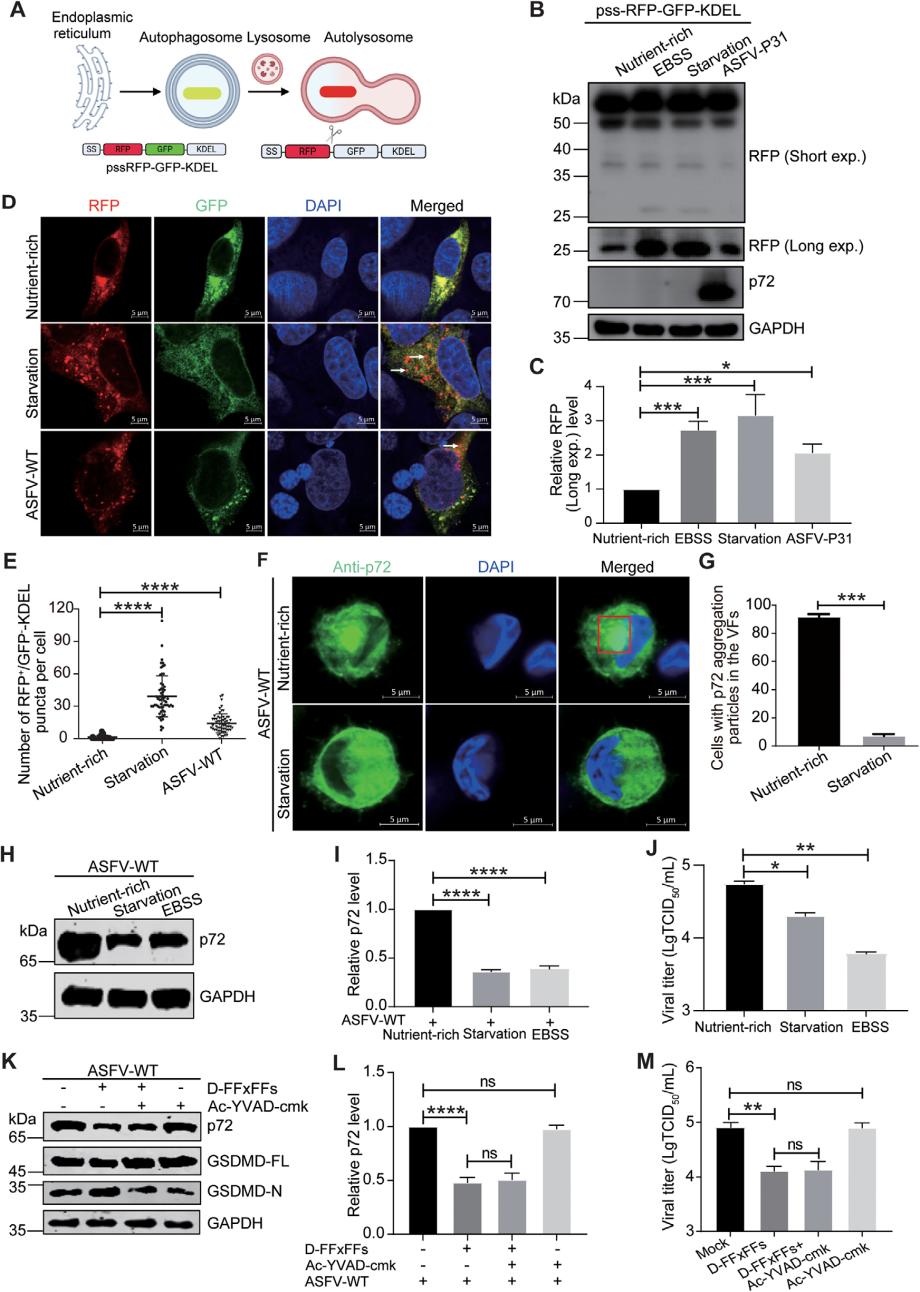
Reticulophagy inhibits ASFV replication. (A) Schematic representation of the reticulophagy reporter. (B–E) Reticulophagy induced by ASFV infection. HEK293T cells were transfected with pssRFP‐GFP‐KDEL and subsequently infected with ASFV‐P31 at a multiplicity of infection (MOI) of 1, or cultured in either nutrient‐rich medium, starvation medium (lacking amino acids) or EBSS as a control group. RFP expression was analyzed by Western blotting at 12 h post‐infection (hpi) (B, C). WSL cells were transfected with pssRFP‐GFP‐KDEL. At 24 h post‐transfection (hpt), the cells were divided into three groups: a nutrient‐rich medium (Control), an amino acid starvation medium (starvation for 12 h), or a nutrient‐rich medium infected with ASFV‐WT at an MOI of 1. Reticulophagy activity was measured using laser confocal microscopy at 12 hpi. Reticulophagy was indicated as RFP^+^/GFP^−^ puncta (white arrowhead) (D). Reticulophagy was quantified for each group in (D) (*n* = 60 cells per group) (E). (F, G) The distribution of the ASFV p72 in PAMs. PAMs were subjected to a 12‐h starvation treatment using high‐glucose D‐MEM medium, which contained sodium salts and lacked amino acids. Subsequently, the cells were infected with ASFV‐WT at an MOI of 1. At 12 hpi, the cells were labeled with anti‐p72 antibodies to observe the intracellular distribution of p72. The red box indicates the p72 aggregate particles in viral factories (VFs). Scale bars, 5 µm (F). The cells with p72 aggregate particles in VFs were quantified for each group (*n* = 100 cells per group) (G). (H–M) Reticulophagy inhibits the replication of ASFV. After treatment of PAMs with amino acid starvation or EBSS for 12 h, the cells were infected with ASFV‐WT at an MOI of 1. At 12 hpi, the p72 expression level (H, I) and viral titers (J) were measured. After treatment with D‐FFxFFs (20 µM) and Ac‐YVAD‐cmk (50 µM) for 12 h as indicated, PAMs were infected with ASFV‐WT (MOI = 1) and the p72 expression level (K, L) and viral titers (M) were measured at 12 hpi. The data are presented as the mean ± SD from three independent experiments. Statistical significance was determined by one‐way ANOVA for the data in panels (C, E, I, J, L, and M), and by two‐tailed unpaired *t*‐test for the data in panel (G) (ns, not significant; *p < 0.05; **p < 0.01; ***p < 0.001; ****p < 0.0001).

### 2.2 Reticulophagy Inhibits ASFV Infection in PAMs

To uncover the relationship between ASFV infection and reticulophagy, we subjected primary porcine alveolar macrophages (PAMs), the target cells for ASFV, to starvation treatment prior to ASFV‐WT infection, followed by observation of VFs changes. Interestingly, compared with the control group, starvation‐induced reticulophagy activation significantly reduced the levels of the major capsid protein p72 in VFs (Figure 1F,G). To test whether reticulophagy affects ASFV VF formation, we examined the localization of p54 (a VF‐associated protein) and vimentin (a VF biomarker) under reticulophagy‐inducing conditions [26, 27]. Both markers retained their characteristic distribution, indistinguishable from controls, demonstrating that VF architecture remains intact (Figure S1A–C). Based on this observation, it was hypothesized that the activation of reticulophagy might inhibit ASFV replication by reducing p72 expression level. To investigate this hypothesis, PAMs were subjected to amino acid starvation or EBSS treatment, and were subsequently infected with ASFV‐WT. The expression of p72 and viral titers were then measured. The results showed that reticulophagy activation significantly reduced p72 expression (Figure 1H,I) and inhibited ASFV replication in PAMs (Figure 1J).

To further validate these findings, a specific reticulophagy activator, the stable short peptide D‐FFxFFs, which has been previously reported to induce reticulophagy [28], was utilized. PAMs were treated with 20 µm D‐FFxFFs, a concentration that was confirmed to have no cytotoxicity in PAMs (Figure S2A–C), to evaluate its effects on p72 expression and ASFV replication. Consistent with the starvation and EBSS treatment results, D‐FFxFFs‐induced reticulophagy significantly downregulated p72 expression and inhibited ASFV replication in PAMs (Figure 1K−M). To exclude the possibility that D‐FFxFFs‐induced pyroptosis contributes to the inhibitory effect on ASFV replication, the caspase‐1‐specific inhibitor Ac‐YVAD‐cmk was administered concurrently with D‐FFxFFs treatment. The results demonstrated that the inhibition of ASFV replication and p72 expression was mediated by reticulophagy activation, but not by pyroptosis inhibition (Figure 1K−M). These data together indicate that reticulophagy can significantly inhibit ASFV replication.

### 2.3 The Reticulophagy Receptor FAM134B Degrades ASFV Capsid Proteins p72 and pA137R and Restricts Viral Replication

Reticulophagy is a process that relies on SARs to recognize clients and deliver them for lysosomal degradation [29, 30]. Therefore, we speculated that the inhibitory effects of reticulophagy on ASFV replication might be attributed to the reduction of ASFV major capsid protein p72 expression mediated by the reticulophagy receptor. To validate this hypothesis, the impact of reticulophagy receptors (SARs) (including FAM134B, RTN3, ATL3, CCPG1, and SEC62) (Figure S3A) on the expression of p72 was assessed. Only FAM134B significantly reduced the expression levels of p72 (Figure 2A). To further analyze whether FAM134B could mediate the degradation of other viral proteins, we performed mass spectrometry analysis, which identified interactions between FAM134B and several ASFV proteins, including pA137R (Figure S3B–D). pA137R is not only a critical virulence‐associated factor of ASFV but also a viral capsid protein [20]. Consequently, we analyzed the effects of FAM134B on pA137R expression and found that FAM134B, but not other reticulophagy receptors, reduced pA137R expression (Figure 2B). Importantly, we confirmed that FAM134B decreased the expression levels of p72 and pA137R in a dose‐dependent manner (Figure 2C,D). To assess the degradation kinetics of p72 and pA137R, cycloheximide (CHX) chase experiments were performed. The results indicated that FAM134B reduced the protein stability of both p72 and pA137R (Figure S4A,B). Moreover, FAM134B significantly reduced the expression of p72 and pA137R at 12, 16, and 24 h post‐infection (hpi) in both WSL cells (Figure 2E) and HEK293T cells (Figure S4C) during ASFV infection, and also inhibited ASFV replication in these cells (Figure 2F and Figure S4D). Altogether, these findings indicate that FAM134B mediates the reduction of ASFV capsid proteins p72 and pA137R, thereby inhibiting viral replication.

**FIGURE 2:**
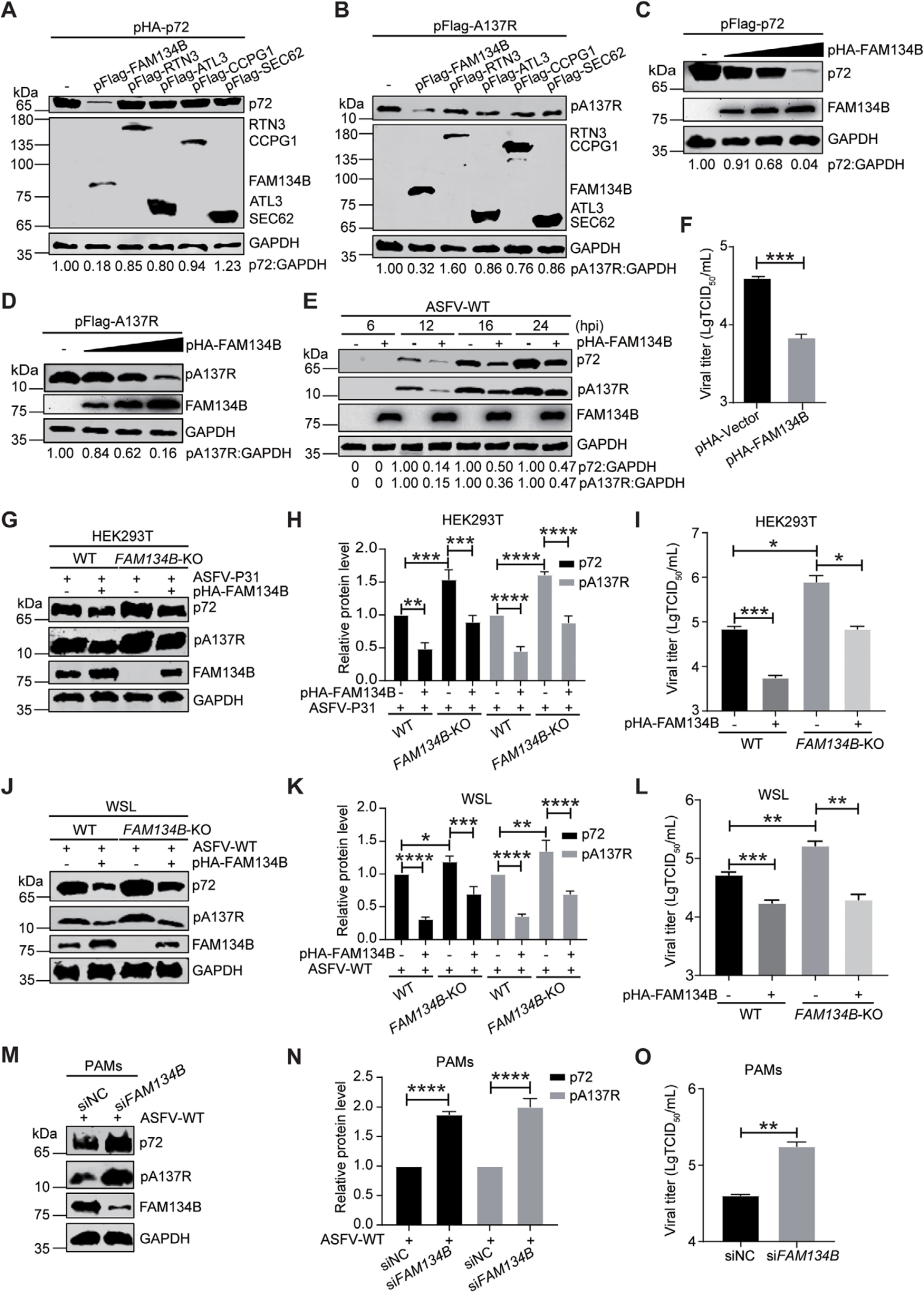
FAM134B mediates the degradation of ASFV capsid proteins. (A–D) FAM134B is associated with a reduction in the expression levels of p72 and pA137R. HEK293T cells were cotransfected with the plasmids expressing five ER‐phagy receptors (FAM134B, RTN3, ATL3, CCPG1, or SEC62) and expression plasmids for either p72 (pHA‐p72) or pA137R (pFlag‐A137R). The expression of p72 (A) and pA137R (B) was analyzed by Western blotting (WB) at 24 h post‐transfection (hpt). HEK293T cells were cotransfected with an increasing amount of pHA‐FAM134B (0.5, 1.0, and 1.5 µg) and pFlag‐p72 or pFlag‐A137R. At 24 hpt, the expression of p72 (C) and pA137R (D) was analyzed by WB. (E, F) FAM134B inhibits the replication of ASFV. WSL cells were transfected with pHA‐FAM134B and subsequently infected with wild‐type ASFV (ASFV‐WT) at a multiplicity of infection (MOI) of 1. The cells were subjected to WB to analyze the expression levels of p72 and pA137R at 6, 12, 16, and 24 h post‐infection (hpi) (E) and titration of viral titers at 24 hpi (F). (G–O) FAM134B degrades ASFV capsid proteins p72 and pA137R to restrict viral replication. *FAM134B*‐knockout (*FAM134B*‐KO) HEK293T or WSL cells were transfected with either an empty vector or pHA‐FAM134B, as indicated. At 24 hpt, the HEK293T cells were infected with cell‐adapted ASFV (ASFV‐P31) at an MOI of 1, while WSL cells were infected with ASFV‐WT at the same MOI. The expression of p72 and pA137R was analyzed by WB (HEK293T: (G, H); WSL: (J, K), and the viral titers were determined at 24 hpi (HEK293T: (I); WSL: (L). PAMs were transfected with either scrambled or *FAM134B*‐specific siRNAs. At 24 hpt, the cells were infected with ASFV‐WT at an MOI of 1. At 12 hpi, the protein levels of p72 and pA137R were examined by WB (M, N). Additionally, viral titers were quantified (O). The data are presented as the mean ± SD from three independent experiments. Statistical significance was determined by one‐way ANOVA for the data in panels (H, I, K, L, and N), and by two‐tailed unpaired *t*‐test for the data in panels (F, O) (*p < 0.05; **p < 0.01; ***p < 0.001; ****p < 0.0001).

Furthermore, we performed loss‐of‐function assays to validate the endogenous antiviral activity of FAM134B during ASFV infection. As shown in Figure 2G,H, *FAM134B*‐KO HEK293T cells exhibited significantly elevated protein levels of p72 and pA137R, along with markedly increased viral titers (Figure 2I), compared with wild‐type HEK293T cells. To confirm the specificity of the observed phenotype, a rescue experiment was performed by reconstituting *FAM134B* expression in *FAM134B*‐KO HEK293T cells. Ectopic re‐expression of *FAM134B* significantly reduced p72 and pA137R expression (Figure 2G,H) and fully restored the restriction of ASFV replication in *FAM134B*‐KO cells (Figure 2I). Importantly, consistent findings were recapitulated in *FAM134B*‐KO WSL cells infected with ASFV‐WT (Figure 2J–L).

To further corroborate these findings in a physiologically relevant in vitro model, we used RNA interference (RNAi) to knockdown endogenous *FAM134B* expression in PAMs, the natural target cells of ASFV in vivo. Consistent with the data from *FAM134B*‐KO HEK293T and *FAM134B*‐KO WSL cell lines, *FAM134B* knockdown increased the expression levels of ASFV p72 and pA137R (Figure 2M,N) and markedly enhanced ASFV‐WT replication in PAMs (Figure 2O). Collectively, these data confirm that FAM134B is a critical host restriction factor that inhibits ASFV infection.

### 2.4 The Degradation of the ASFV Capsid Proteins p72 and pA137R Mediated by FAM134B Inhibits the Maturation of ASFV Virions in VFs

Given that FAM134B mediates the autophagic degradation of ASFV capsid proteins, we presumed that FAM134B might inhibit ASFV capsid assembly in VFs. Indeed, the formation of mature viral particles in VFs was found to be significantly inhibited by FAM134B via transmission electron microscopy analysis, relative to the empty vector (EV)‐transfected control cells (Figure 3A,B). The inhibitory effects of FAM134B on ASFV virion maturation in VFs were further validated by confocal imaging (Figure 3C,D). Considering that p72 oligomerization is indispensable for ASFV capsid assembly [18], we assessed the effects of FAM134B on this process. As shown in Figure 3E, FAM134B inhibited the formation of p72 oligomers in a dose‐dependent manner. To investigate whether the inhibitory effects occurred during ASFV infection, WSL cells were transfected with pHA‐FAM134B and subsequently infected with ASFV‐WT at a multiplicity of infection (MOI) of 1. Analysis of p72 oligomerization showed that FAM134B inhibited this process during ASFV infection (Figure 3F). Altogether, these results indicate that FAM134B significantly inhibits ASFV capsid assembly in VFs.

**FIGURE 3:**
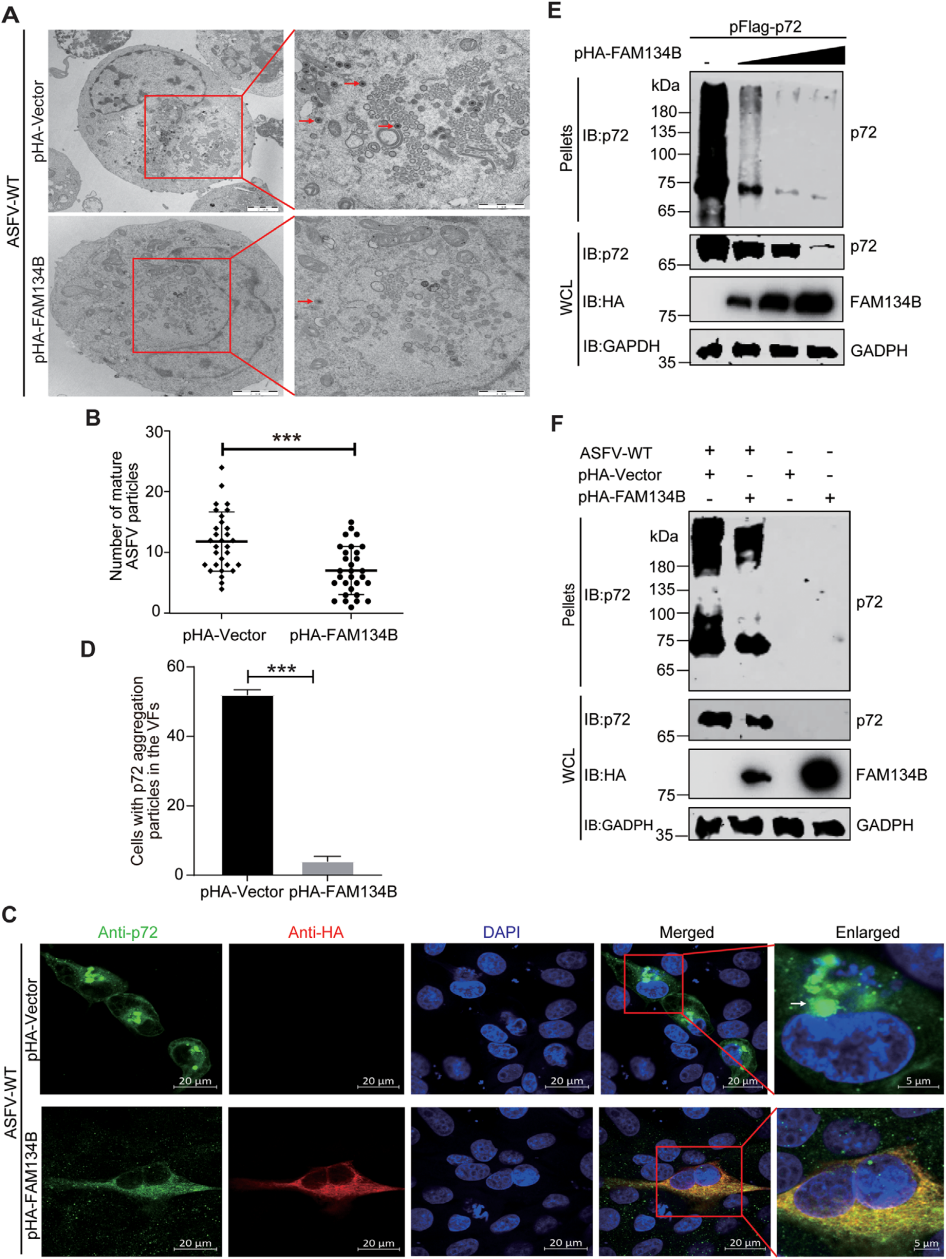
FAM134B inhibits the ASFV capsid assembly in viral factories (VFs). (A–D) FAM134B inhibits the formation of mature virions in VFs. WSL cells were transfected with pHA‐FAM134B, followed by infection with ASFV‐WT at a multiplicity of infection (MOI) of 5 at 24 h post‐transfection (hpt). Transmission electron microscopy (TEM) was utilized to observe the morphology of the viral particles in VFs at 12 h post‐infection (hpi). Scale bars, 2 µm (TEM)  or 1 µm  (insets). The red arrowhead represents a mature virion (A). The number of mature virions in VFs was quantified for each group (*n* = 30) (B). WSL cells were transfected with pHA‐FAM134B and subsequently infected with ASFV‐WT at an MOI of 1 at 24 hpt. Confocal imaging was employed to analyze the intracellular distribution of p72 at 12 hpi. The white arrowhead represents p72 aggregate particles in VFs. Scale bars, 20 µm for images, 5 µm for magnified images (C). The cells with p72 aggregate particles in VFs were quantified for each group (*n* = 60) (D). (E, F) FAM134B inhibits the oligomerization of p72. HEK293T cells were cotransfected with an increasing amount of pHA‐FAM134B (0.5, 1.0, and 1.5 µg) and pFlag‐p72. The cells were cross‐linked using disuccinimidyl suberate crosslinker (DSS) at 24 hpt. The formation of p72 oligomers was assessed by immunoblotting (E). WSL cells were transfected with pHA‐FAM134B. The cells were infected with ASFV‐WT at an MOI of 1 and then cross‐linked using DSS at 12 hpi. The formation of p72 oligomers was assessed by immunoblotting with the indicated antibodies (F). The data are presented as the mean ± SD from three independent experiments. Statistical significance of the data in (B, D) was analyzed two‐tailed unpaired *t*‐test (**p < 0.01; ***p < 0.001).

### 2.5 FAM134B Interacts With the ASFV Capsid Proteins p72 and pA137R

To elucidate the molecular mechanism underlying the inhibitory effect of FAM134B on ASFV replication, we examined the interaction between FAM134B and the two key viral capsid proteins. Co‐immunoprecipitation (Co‐IP) assay showed that FAM134B specifically interacted with both p72 and pA137R (Figure 4A,B). Consistently, the association between FAM134B and the two capsid proteins in ASFV‐infected cells was confirmed (Figure S5A). Furthermore, endogenous Co‐IP assay revealed that p72 and pA137R were specifically co‐precipitated with FAM134B (Figure 4C). To delineate the interaction domains of FAM134B, we constructed serial plasmids expressing FAM134B mutants lacking the N‐terminus, reticulon homology domain (RHD), or C‐terminus (Figure S5B). Co‐IP assay indicated that the C‐terminus of FAM134B interacted with p72 and pA137R (Figure S5C,D). Collectively, these data consistently demonstrate that FAM134B interacts with the ASFV capsid proteins p72 and pA137R.

**FIGURE 4:**
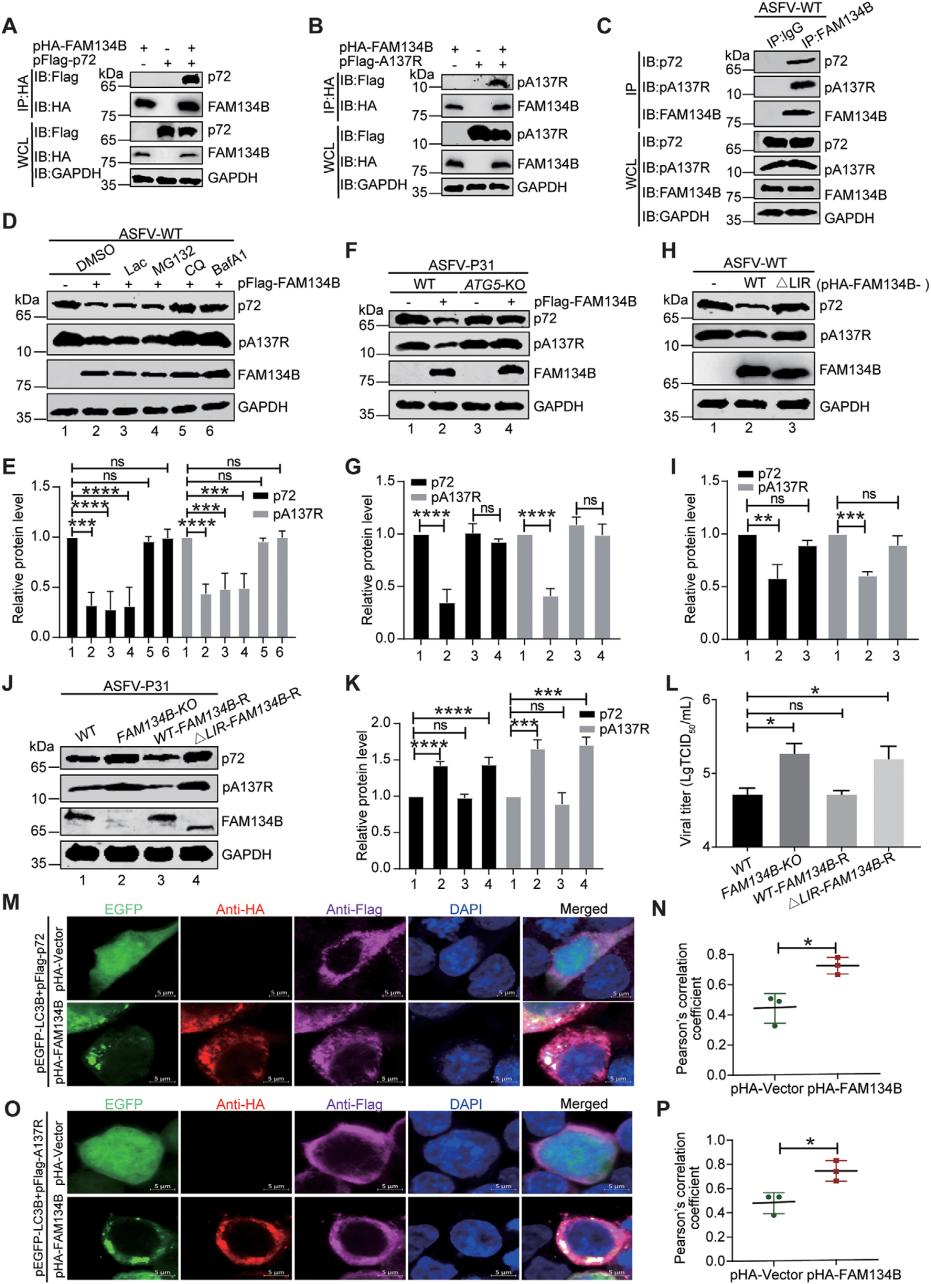
FAM134B targets the ASFV proteins p72 and pA137R for degradation via reticulophagy. (A–C) FAM134B interacts with p72, and pA137R. HEK293T cells were cotransfected with pFlag‐p72 (A) or pFlag‐A137R (B) together with pHA‐FAM134B. The cells were lysed and whole cell lysates (WCL) were immunoprecipitated with an anti‐HA monoclonal antibody (mAb) at 36 h post‐transfection (hpt). The immunoprecipitates were examined by immunoblotting (IB). WSL cells were infected with ASFV‐WT at a multiplicity of infection (MOI) of 1. At 24 h post‐infection (hpi), the cells were lysed for immunoprecipitation (IP) with anti‐FAM134B. Anti‐IgG antibodies were used as a negative control (C). (D–G) FAM134B degrades p72 and pA137R via the autophagy‐lysosomal pathway. WSL cells were transfected with pFlag‐FAM134B, followed by infection with ASFV‐WT at an MOI of 1 and treatment with Lac (20 µM), MG132 (20 µM), CQ (50 µM), or BafA1 (100 nM) for 8 h at 16 hpi. The cell lysates were analyzed by IB (D, E). The *ATG5*‐knockout (*ATG5‐*KO) or wild‐type (WT) HEK293T cells were transfected with pFlag‐FAM134B, followed by infection with ASFV‐P31 at an MOI of 1. At 24 hpi, the expression levels of p72, pA137R, and FAM134B were analyzed by IB (F, G). (H–L) The degradation of p72 and pA137R mediated by FAM134B depends on the LIR motif of FAM134B. WSL cells were transfected with pHA‐FAM134B or pHA‐FAM134B‐ΔLIR, followed by infection with ASFV‐WT at an MOI of 1. At 24 hpi, the expression levels of p72, pA137R, and FAM134B were analyzed by IB (H, I). Wild‐type HEK293T cells, along with *FAM134B*‐KO cells reconstituted with wild‐type *FAM134B* (WT‐*FAM134B*‐R) or the LIR mutant (ΔLIR‐*FAM134B*‐R), were infected with ASFV‐P31 at an MOI of 1. At 24 hpi, the expression levels of p72, pA137R, and FAM134B were analyzed by IB (J, K). Additionally, viral titers were quantified (L). (M–P) Co‐localization of FAM134B with LC3B and ASFV p72 (M, N) or pA137R (O, P). HEK293T cells were cotransfected with the indicated plasmids and protein colocalization was analyzed by confocal microscopy. The data are presented as the mean ± SD from three independent experiments. Statistical significance was determined by one‐way ANOVA for the data in panels (E, G, I, K, and L), and by two‐tailed unpaired *t*‐test for the data in panels (N) and (P) (ns, not significant; *p < 0.05; ***p < 0.001; ****p < 0.0001).

### 2.6 LIR‐Dependent Autophagic Degradation of p72 and pA137R by FAM134B Is Required for Its Antiviral Function

To elucidate the molecular mechanism underlying the FAM134B‐mediated degradation of the ASFV capsid proteins p72 and pA137R, pharmacological inhibition assay was performed. The results showed that autophagic flux inhibitors chloroquine (CQ) and bafilomycin A1 (BafA1) treatment significantly rescued the FAM134B‐mediated reduction of endogenous p72 and pA137R, whereas no such rescue effect was observed with ubiquitin‐proteasome inhibitors lactacystin (Lac) and carbobenzoxy‐Leu‐Leu‐Leucinal (MG132) treatment (Figure 4D,E). To clarify whether the autophagy pathway is required, we next examined the effects of FAM134B in *ATG5*‐KO and *ATG7*‐KO cells, since ATG5 and ATG7 are essential components of the autophagy conjugation machinery and are indispensable for autophagosome formation. The FAM134B‐mediated degradation of endogenous p72 and pA137R was abolished in *ATG5*‐KO (Figure 4F,G) or *ATG7*‐KO (Figure S5E,F) HEK293T cells, compared with wild‐type HEK293T cells. Taken together, these data indicate that the FAM134B‐mediated degradation of p72 and pA137R is dependent on the autophagy‐lysosome pathway.

Next, the Western blotting analysis revealed that reduction of both endogenous p72 and pA137R expression was induced by wild type FAM134B, whereas the LIR‐deleted mutant FAM134B lost the ability to mediate the degradation of these two viral proteins (Figure 4H,I). To further validate the LIR motif‐dependent of FAM134B‐mediated antiviral activity, a rescue assay was performed in *FAM134B*‐KO HEK293T cells reconstituted with wild‐type *FAM134B* (WT‐*FAM134B*‐R) or LIR‐deleted *FAM134B* (ΔLIR‐*FAM134B*‐R). As shown in Figure 4J,K, the expression levels of p72 and pA137R were comparable between wild‐type and WT‐*FAM134B*‐R cells. In contrast, a significant upregulation of p72 and pA137R expression was observed in *FAM134B‐*KO cells and ΔLIR‐*FAM134B*‐R cells. Consistently, as presented in Figure 4L, the replication levels of ASFV were similar in *FAM134B‐*KO cells and ΔLIR‐*FAM134B*‐R cells, which were significantly higher than those detected in wild‐type cells and WT‐*FAM134B*‐R cells. Altogether, these data demonstrate that reconstitution of FAM134B in *FAM134B*‐knockout cells inhibits ASFV replication.

Having confirmed that FAM134B mediates the degradation of p72 and pA137R in an LIR motif‐dependent manner via its reticulophagy receptor function, we performed confocal imaging to analyze the colocalization of FAM134B with the ASFV capsid proteins, paired with either the LC3B or the LAMP1, respectively. Confocal imaging revealed that the colocalization between p72 and LC3B was significantly increased in the presence of FAM134B (Figure 4M,N). Similarly, the colocalization between pA137R and LC3B was also significantly increased under the same condition (Figure 4O,P). Furthermore, we detected a significant increase in mCherry‐LAMP1‐positive lysosomal puncta colocalized with FAM134B and p72 (Figure S5G,H), as well as those colocalized with FAM134B and pA137R (Figure S5I,J). Collectively, these findings confirm that the FAM134B‐mediated reticulophagy degrades p72 and pA137R, thereby inhibiting the ASFV capsid assembly in VFs.

### 2.7 FAM134B Is Targeted by ASFV pMGF300‐2R, a Virulence‐Associated Factor

To investigate whether ASFV antagonizes the antiviral activity of FAM134B, the expression dynamics of FAM134B following ASFV infection were analyzed. It was found that ASFV‐WT infection induced a significant reduction in FAM134B protein levels at 12, 16, and 24 hpi (Figure 5A,B), and this downregulation of FAM134B presented in a dose‐dependent manner in PAMs (Figure 5C,D). Additionally, as shown in Figure 5E, FAM134B mRNA levels remained stable following ASFV infection, suggesting that the decrease in FAM134B protein levels was not a result of diminished gene transcription. We assumed that ASFV‐encoded enzymatically active proteins may down‐regulate the expression of FAM134B. Consequently, we prioritized the investigation of pI215L, which exhibits E2 ubiquitin ligase activity, and pS273R, an SUMO‐1‐specific cysteine protease [31, 32]. Unfortunately, the results indicated that the S273R and I215L proteins did not affect the expression of FAM134B (Figure S6A,B). To identify ASFV proteins that mediate the reduction of FAM134B, HEK293T cells were cotransfected with pHA‐FAM134B and each individual plasmid from an ASFV open reading frame (ORF) library encoding 61 viral proteins. Western blotting analysis demonstrated that only pMGF300‐2R, a critical virulence‐associated factor of ASFV [33], significantly reduced the expression of FAM134B (Figure 5F and Figure S6C–K) and in a dose‐dependent manner (Figure 5G,H). Furthermore, in contrast with ASFV‐WT infection, PAMs infected with *MGF300‐2R*‐deleted mutant ASFV (ASFV‐Del2R) demonstrated a significant increase in FAM134B expression (Figure 5I,J). Overall, these findings indicate that the ASFV virulence‐associated factor pMGF300‐2R can induce the reduction of FAM134B expression.

**FIGURE 5:**
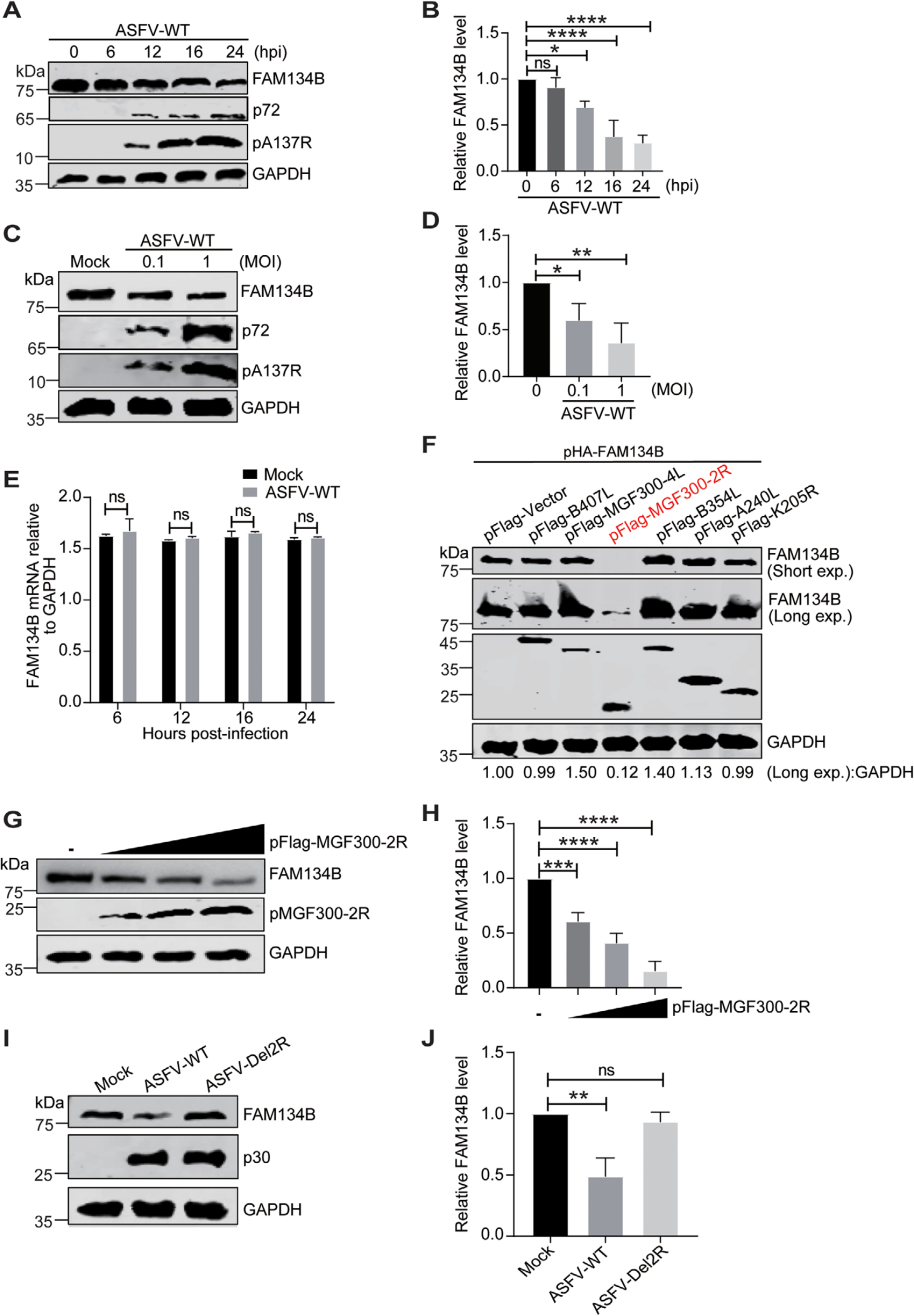
The degradation of FAM134B is mediated by the pMGF300‐2R. (A–D) ASFV reduces the expression of FAM134B. PAMs were infected with ASFV‐WT at a multiplicity of infection (MOI) of 1. The expression of FAM134B was analyzed by immunoblotting (IB) at 0, 6, 12, 16, and 24 h post‐infection (hpi) (A, B). PAMs were infected with ASFV‐WT at an MOI of 0.1 or 1. The cells were subjected to IB to assess FAM134B expression at 24 hpi (C, D). (E) ASFV does not influence the transcription of FAM134B. PAMs were infected with ASFV‐WT at an MOI of 1. FAM134B mRNA levels were analyzed by RT‐qPCR at 6, 12, 16, and 24 hpi. (F) The effects of ASFV‐encoded proteins on FAM134B expression. HEK293T cells were cotransfected with the plasmids expressing ASFV proteins (pB407L, pMGF300‐4L, pMGF300‐2R, pB354L, pA240L, and pK205R) along with pHA‐FAM134B. The cells were subjected to IB at 24 h post‐transfection (hpt) to evaluate the expression of FAM134B. (G, H) pMGF300‐2R reduced the expression of FAM134B in a dose‐dependent manner. WSL cells were cotransfected with an increasing amount of pFlag‐MGF300‐2R (0.5, 1.0, and 1.5 µg). At 24 hpt, FAM134B expression was analyzed by IB. (I) and (J) pMGF300‐2R reduces the expression of FAM134B and promotes ASFV replication. PAMs were infected with ASFV‐WT or ASFV mutant lacking the *MGF300‐2R* gene (ASFV‐Del2R). The cells were subjected to IB at 24 hpi to assess the expression of FAM134B. The data are presented as the mean ± SD from three independent experiments. Statistical significance was determined by one‐way ANOVA for the data in panels (B, D, H, and J), and by two‐tailed unpaired *t*‐test for the data in panel (E) (ns, not significant; *p < 0.05; **p < 0.01; ***p < 0.001; ****p < 0.0001).

### 2.8 pMGF300‐2R Facilitates FAM134B Degradation via the Autophagy‐Lysosomal Pathway

To gain insight into the molecular mechanism by which pMGF300‐2R reduces the expression of FAM134B, we analyzed the interaction between pMGF300‐2R and FAM134B. The Co‐IP assay indicated an interaction between pMGF300‐2R and FAM134B (Figure 6A). This interaction was further validated through a glutathione S‐transferase (GST) pulldown assay, in which His‐tagged pMGF300‐2R was pulled down by GST‐tagged FAM134B (Figure 6B). Furthermore, confocal imaging indicated the colocalization between pMGF300‐2R and FAM134B in the cytoplasm of the cells (Figure 6C). To determine whether this interaction occurred in the context of ASFV infection, HEK293T cells were cotransfected with the plasmid expressing EGFP‐tagged pMGF300‐2R under the control of the *MGF300‐2R* promoter and pHA‐FAM134B, followed by infection with ASFV‐P31. As expected, there was a colocalization between pMGF300‐2R and FAM134B (Figure 6D). Taken together, these data indicate that pMGF300‐2R interacts with FAM134B.

**FIGURE 6:**
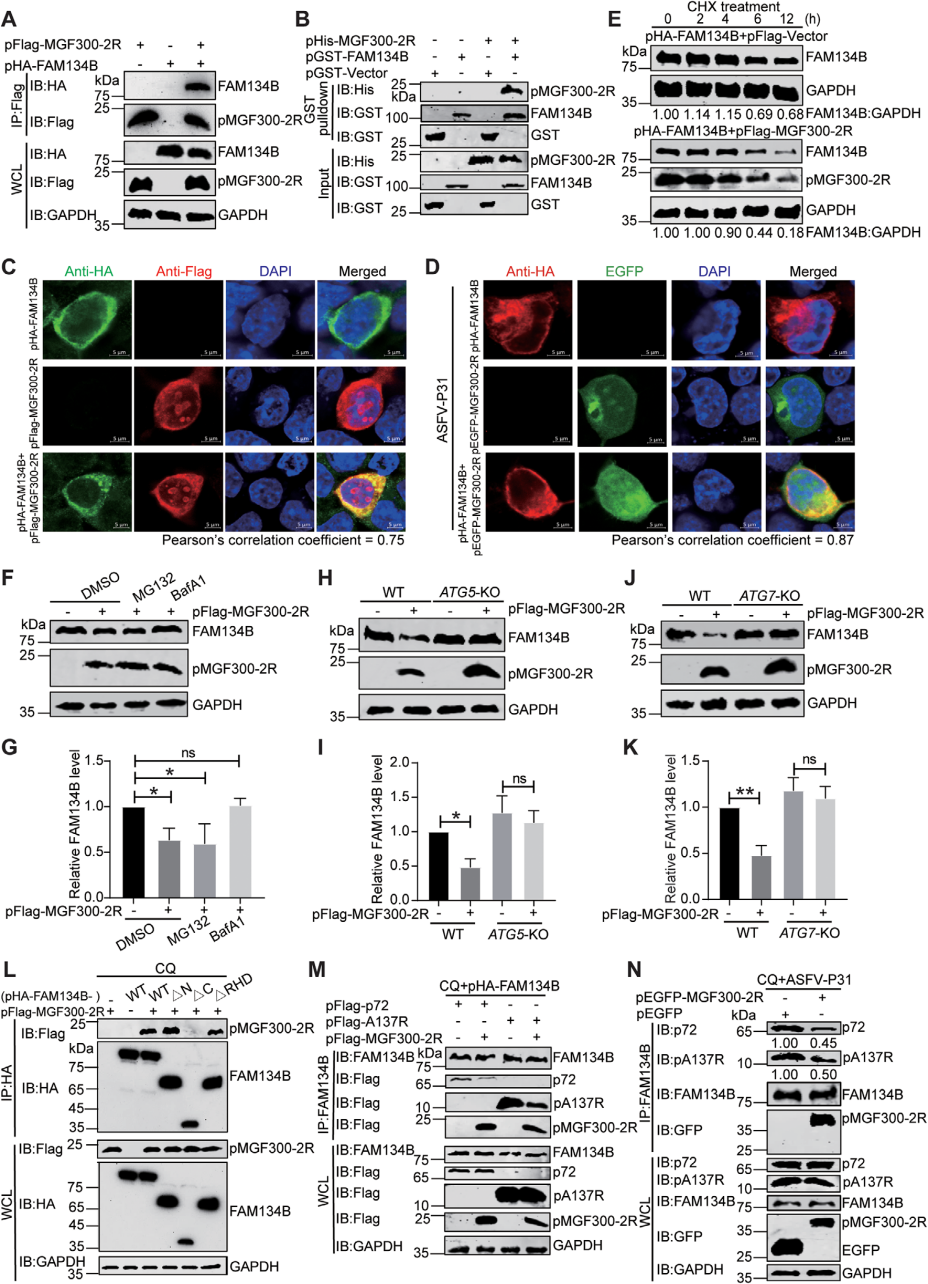
pMGF300‐2R targets FAM134B for degradation via the autophagy‐lysosomal pathway. (A–D) pMGF300‐2R interacts with FAM134B. HEK293T cells were cotransfected with pFlag‐MGF300‐2R and pHA‐FAM134B. The cells were lysed, and whole cell lysates (WCL) were immunoprecipitated with an anti‐Flag monoclonal antibody (mAb). The immunoprecipitates were examined by immunoblotting (IB) with the indicated antibodies (A). The purified GST and GST‐tagged FAM134B proteins were used to pulldown the purified His‐MGF300‐2R (B). HEK293T cells were cotransfected with pFlag‐MGF300‐2R and pHA‐FAM134B. At 24 h post‐transfection (hpt), colocalization was analyzed using laser confocal microscopy. Scale bars, 5 µm. (C). HEK293T cells were transfected with pEGFP‐MGF300‐2R and pHA‐FAM134B. At 24 hpt, the cells were infected with ASFV‐P31 for colocalization analysis. Scale bars, 5 µm (D). The colocalization of FAM134B and pMGF300‐2R was analyzed by the Coloc2 tool of ImageJ/FIJI and shown as Pearson's correlation coefficients (C, D). (E) Cycloheximide (CHX) chase assay. HEK293T cells cotransfected with either pFlag‐Vector or pFlag‐MGF300‐2R alongside pHA‐FAM134B. Following treatment with CHX (100 µg/mL), the cells were harvested at the indicated time points, and total cell lysates were subjected to IB analysis. (F–K) pMGF300‐2R targets FAM134B for degradation via autophagy pathway. WSL cells were transfected with pFlag‐MGF300‐2R. At 16 hpt, the cells were treated with MG132 (20 µM), or bafilomycin A1 (BafA1, 100 nM) for 8 h. Subsequently, the cell lysates were analyzed by IB (F, G). Wild‐type (WT), *ATG5*‐knockout (*ATG5*‐KO) (H, I), and *ATG7*‐knockout (*ATG7*‐KO) (J, K) HEK293T cells were transfected with pFlag‐MGF300‐2R. At 24 hpt, the expression levels of FAM134B and pMGF300‐2R were analyzed by IB. (L) The interaction between pMGF300‐2R and a series of truncated forms of FAM134B was analyzed by co‐immunoprecipitation assay. (M, N) pMGF300‐2R competes with p72 and pA137R for binding to FAM134B. HEK293T cells were cotransfected with pFlag‐MGF300‐2R, pHA‐FAM134B, pFlag‐p72, and pFlag‐A137R, as indicated. After treatment with CQ, the cell lysates were used for IP with anti‐FAM134B, followed by IB with the indicated antibodies (M). HEK293T cells were transfected with pEGFP‐MGF300‐2R or pEGFP and treated with CQ. At 24 hpt, the cells were infected with ASFV‐P31 at an MOI of 1. At 24 hpi, the cell lysates were subjected to IP assay with anti‐FAM134B antibodies, followed by IB with the indicated antibodies (N). The data are presented as the mean ± SD from three independent experiments. Statistical significance of the data in (G, I, and K) was analyzed by one‐way ANOVA (ns, not significant; *p < 0.05; **p < 0.01).

Next, we conducted a CHX chase assay to assess the degradation kinetics of FAM134B. The results indicated that pMGF300‐2R reduced the stability of FAM134B protein (Figure 6E). To elucidate the molecular basis by which pMGF300‐2R mediated FAM134B degradation, the results of inhibitor analysis showed that treatment with BafA1, but not with MG132, exhibited the most pronounced effects in counteracting the reduction of endogenous FAM134B (Figure 6F,G). Moreover, knockout of the *ATG5* or *ATG7* gene abolished the pMGF300‐2R‐mediated degradation of endogenously expressed FAM134B (Figure 6H–K). Collectively, these findings suggest that pMGF300‐2R facilitates the degradation of FAM134B via the autophagy‐lysosomal pathway.

Subsequently, we mapped the protein domain(s) involved in the interaction between pMGF300‐2R and FAM134B. The Co‐IP result indicated the interaction between pMGF300‐2R and FAM134B mediated by the C‐terminus of FAM134B (Figure 6L). To clarify whether pMGF300‐2R competes with p72 or pA137R for FAM134B binding, the FAM134B‐p72/A137R interactions were analyzed by Co‐IP assay in the presence of pMGF300‐2R. The results showed that pMGF300‐2R reduced the binding of p72 and pA137R to FAM134B (Figure 6M). We next validated this competitive binding effect under conditions of ASFV infection using an endogenous Co‐IP assay with anti‐FAM134B antibody. The results confirmed that pMGF300‐2R significantly impaired the interaction between endogenous FAM134B and ASFV encoded p72 and pA137R (Figure 6N). These data show that the interaction between pMGF300‐2R and FAM134B competitively inhibits the binding of the p72 and pA137R to FAM134B, thereby inhibiting the antiviral function of FAM134B.

In summary, these data suggest pMGF300‐2R binds to FAM134B, leading to autophagic degradation of FAM134B, thereby supporting that pMGF300‐2R antagonizes the antiviral function of FAM134B.

### 2.9 pMGF300‐2R Is an LIR Motif‐Containing Interactor of LC3B

To determine whether pMGF300‐2R degrades FAM134B via the same mechanism reported for IKK*α*/*β* degradation [33], the FAM134B expression in *TOLLIP*‐KO or *p62*‐KO cells was assessed. The immunoblotting analysis indicated that the degradation of FAM134B mediated by pMGF300‐2R was independent of both SARs (Figure S7A,B). This finding rules out TOLLIP and p62 involvement and suggests a novel mechanism through which pMGF300‐2R counteracts the antiviral activity mediated by FAM134B.

It has been reported that several RNA viruses, such as influenza A virus and Rift Valley fever virus, encode LIR motif‐containing proteins that act as molecular mimics of autophagy receptors to bind LC3B and subvert host immunity [34]. Considering that pMGF300‐2R interacts with FAM134B and targets it for autophagic degradation, we hypothesized that pMGF300‐2R itself might function as a viral autophagy receptor. Bioinformatics analysis predicted three canonical LIR motifs (i.e., LIR1, LIR2, and LIR3) in the C‐terminal region of pMGF300‐2R (Figure 7A). Since the LIR motif is important for the association between autophagy receptors and ATG8 family proteins, we examined whether pMGF300‐2R interacts with LC3B. Confocal imaging revealed that colocalization of pMGF300‐2R and LC3B occurred in the cytoplasm (Figure 7B). Furthermore, this interaction was further confirmed by Co‐IP assay in both exogenous (Figure 7C) and endogenous (Figure 7D) levels.

**FIGURE 7:**
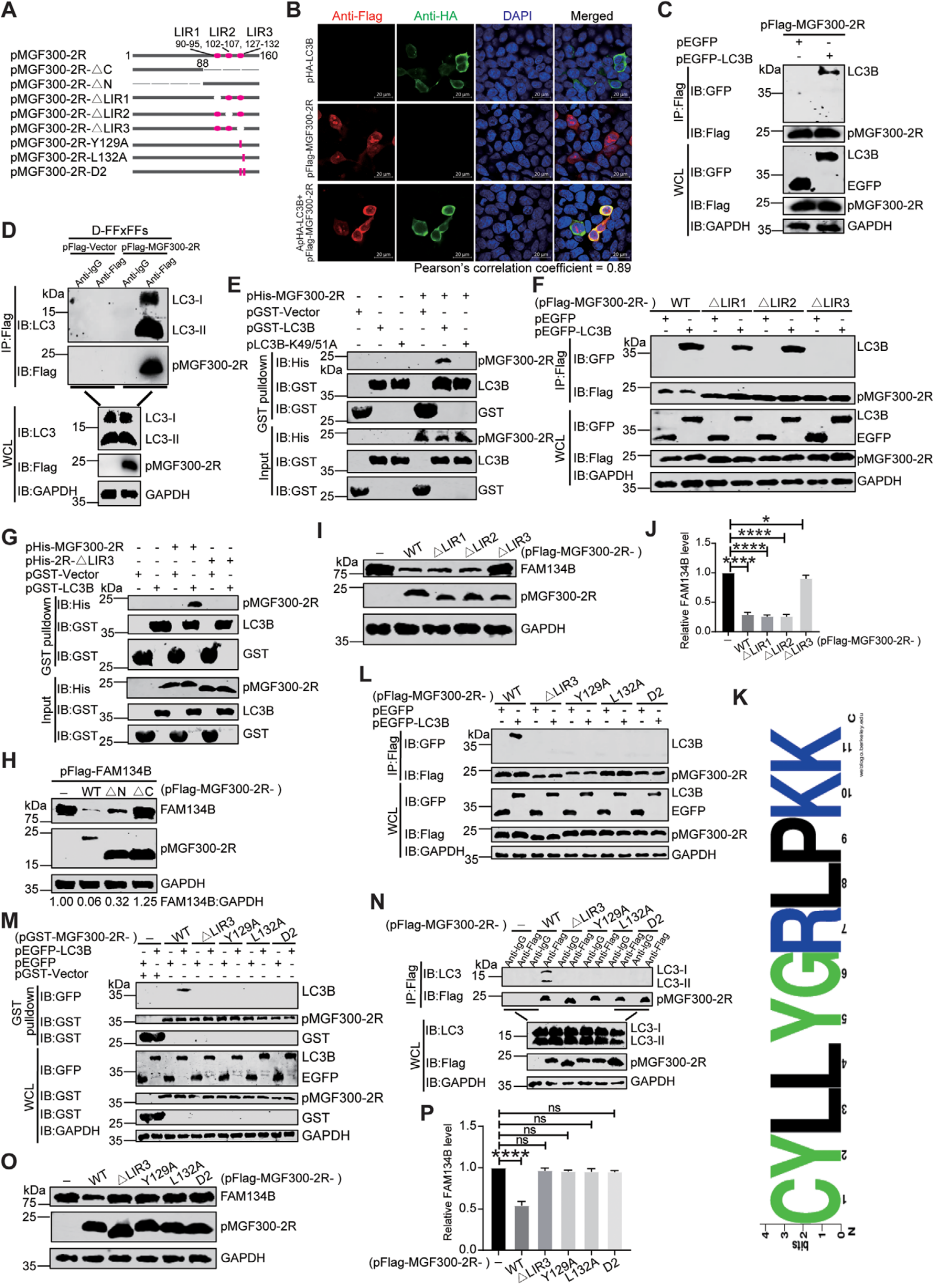
pMGF300‐2R binds to LC3B via its C‐terminal LIR3 motif and mediates the degradation of FAM134B. (A–E) pMGF300‐2R interacts with LC3B. Schematic representation of different pMGF300‐2R mutants (A). HEK293T cells were cotransfected with pFlag‐MGF300‐2R and pHA‐LC3B. At 24 h post‐transfection (hpt), the colocalization of pMGF300‐2R and LC3B was analyzed by confocal microscopy and the Coloc2 tool of ImageJ/FIJI and shown as Pearson's correlation coefficients. Scale bars, 20 µm (B). HEK293T cells were cotransfected with pFlag‐MGF300‐2R and pEGFP‐LC3B. The cells were lysed and whole cell lysates (WCL) were immunoprecipitated with an anti‐Flag monoclonal antibody. The immunoprecipitates were examined by immunoblotting (IB) (C). HEK293T cells were transfected with pFlag‐MGF300‐2R. At 24 hpt, the cells were treated with D‐FFxFFs for 12 h. At 36 hpt, the cells were lysed and WCL were immunoprecipitated with an anti‐Flag monoclonal antibody. The immunoprecipitates were examined by IB (D). The purified recombinant GST‐tagged LC3B (and its mutants) and His‐tagged pMGF300‐2R from *E. coli* were analyzed by in vitro GST pulldown assay (E). (F, G) pMGF300‐2R interacts with LC3B via its LIR3 motif. HEK293T cells were cotransfected with pFlag‐MGF300‐2R (WT, ΔLIR1, ΔLIR2, or ΔLIR3) and pEGFP‐LC3B. Co‐immunoprecipitation (Co‐IP) assay was conducted using anti‐Flag beads at 24 hpt (F). The purified recombinant His‐tagged pMGF300‐2R (WT and its mutants) and GST‐tagged LC3B from *E. coli* were analyzed by in vitro GST pulldown assay (G). (H–P) pMGF300‐2R degradation of FAM134B is dependent on its LIR3 motif. HEK293T cells were cotransfected with pFlag‐FAM134B and the plasmids expressing different pMGF300‐2R mutants (WT, ΔN, and ΔC). At 24 hpt, the expression levels of pMGF300‐2R and FAM134B were analyzed by IB (H). WSL cells were transfected with the plasmids expressing Flag‐tagged MGF300‐2R mutants (WT, ΔLIR1, ΔLIR2, and ΔLIR3). At 24 hpt, the cells were harvested and analyzed by IB (I and J). Analysis of amino acid conservation of LIR3 on pMGF300‐2R across different ASFV genotypes. The sequence logos were made using WebLogo 3.6.0 (K). HEK293T cells were cotransfected with pEGFP‐LC3B and the plasmids expressing Flag‐tagged MGF300‐2R mutants (WT, ΔLIR3, Y129A, L132A, and D2). At 36 hpt, the cell lysates were subjected to Co‐IP assay (L). Purified GST or GST‐MGF300‐2R proteins (WT, ΔLIR3, Y129A, L132A, and D2) were used for pulldown assay with the lysates from the pEGFP‐LC3B‐transfected HEK293T cells (M). HEK293T cells were transfected with pMGF300‐2R constructs (WT, ΔLIR3, Y129A, L132A, and D2). At 24 hpt, the cells were treated with D‐FFxFFs for 12 h. At 36 hpt, the cell lysates were subjected to Co‐IP assay (N). WSL cells were transfected the plasmids expressing Flag‐tagged with WT MGF300‐2R or mutants (ΔLIR3, Y129A, L132A, and D2). At 24 hpt, the cell lysates were analyzed by IB (O and P). The data are presented as the mean ± SD from three independent experiments. Statistical significance of the data in (J) and (P) was analyzed by one‐way ANOVA (ns, not significant; *p < 0.05; ***p < 0.001; ****p < 0.0001).

To further verify the interaction between pMGF300‐2R and LC3B, we purified the recombinant GST‐tagged LC3B and His‐tagged pMGF300‐2R from *Escherichia coli*, and performed in vitro GST pulldown assay. The results confirmed that pMGF300‐2R bound to LC3B (Figure 7E). To further validate that this interaction follows the canonical LIR‐ATG8 family proteins binding mode, we generated a K49/51A LC3B mutant, in which the two key lysine residues within the conserved LIR‐docking hydrophobic interface were mutated to alanine. In vitro GST pulldown assay showed that this mutant completely lost its binding capacity to pMGF300‐2R (Figure 7E), confirming that the pMGF300‐2R‐LC3B interaction relies on the LIR‐binding pocket of LC3B.

To identify which of the LIR motifs mediates the interaction between pMGF300‐2R and LC3B, we generated a series of pMGF300‐2R mutants with deletion of each LIR motif (designated pMGF300‐2R‐ΔLIR1, pMGF300‐2R‐ΔLIR2, and pMGF300‐2R‐ΔLIR3) (Figure 7A). Co‐IP assay revealed that LC3B interaction was disrupted by deletion of LIR3 but not LIR1 and LIR2 (Figure 7F). Likewise, the *LIR3*‐deleted pMGF300‐2R failed to pull down LC3B in vitro (Figure 7G). Therefore, the LIR3 motif of pMGF300‐2R specifically mediates its interaction with LC3B.

### 2.10 The LIR3 of pMGF300‐2R Is Required for the Autophagic Degradation of FAM134B

To map the LIR motif required for FAM134B degradation, we generated pMGF300‐2R mutants lacking either the N‐terminal or C‐terminal region (Figure 7A). Notably, the C‐terminally truncated mutant, which lacked the three LIR motifs, failed to degrade FAM134B compared with the wild type pMGF300‐2R (Figure 7H). Functional assay showed that only the pMGF300‐2R‐ΔLIR3 lost the ability to degrade endogenous FAM134B, while pMGF300‐2RΔLIR1 and pMGF300‐2R‐ΔLIR2 retained full degradation activity (Figure 7I,J). Furthermore, sequence analysis revealed that the LIR3 motif (^127^LLYGRL^132^) is highly conserved among genotypes I, II, and I/II recombinant ASFV strains circulating globally (Figure 7K; Table S1).

The LIR3 motif conforms to the canonical [W/F/Y]xx[L/I/V] pattern, with tyrosine at position 129 (Y^129^) and leucine at position 132 (L^132^) predicted to be the critical residues interacting with LC3B [35, 36]. To test this, we generated point mutants substituting Y^129^ and/or L^132^ with alanine. Co‐IP and GST pulldown assays demonstrated that both single (Y129A or L132A) and double (D2) mutations disrupted the pMGF300‐2R–LC3B interaction (Figure 7L–N). Consistently, these point mutations also abrogated the ability of pMGF300‐2R to mediate the degradation of endogenous FAM134B (Figure 7O,P). Collectively, these results demonstrate that LIR3, through its critical residues Y^129^ and L^132^, mediates the interaction between pMGF300‐2R and LC3B, thereby facilitating FAM134B degradation.

### 2.11 The Autophagic Degradation of FAM134B Mediated by pMGF300‐2R Antagonizes Its Antiviral Function, Thus Promoting ASFV Infection and Pathogenicity

To explore the antagonistic effects of pMGF300‐2R on antiviral function mediated by FAM134B, WSL cells were transfected with pFlag‐FAM134B and subsequently infected with ASFV‐WT or ASFV‐Del2R, followed by laser confocal microscopy analysis of reticulophagy. The confocal imaging revealed that the FAM134B‐mediated reticulophagy was significantly induced in WSL cells infected with ASFV‐Del2R compared to those infected with ASFV‐WT (Figure 8A,B). Furthermore, the results of the immunoblotting analysis indicated that reticulophagy was significantly activated in ASFV‐Del2R‐infected WSL compared with that in ASFV‐WT‐infected cells (Figure 8C). Moreover, pMGF300‐2R significantly inhibited the FAM134B‐mediated reticulophagy activation (Figure 8D). More importantly, immunoblotting results showed that FAM134B can degrade p72 and pA137R expressed by ASFV‐WT or ASFV‐Del2R (Figure 8E,F). Furthermore, compared with the degradation of p72 and pA137R expressed by ASFV‐WT, FAM134B exhibited more significant degradation effects on p72 and pA137R expressed by ASFV‐Del2R (Figure 8E,F). The inhibitory effects of FAM134B on ASFV‐Del2R were significantly greater than those on the replication of ASFV‐WT (Figure 8G). The above results show that pMGF300‐2R antagonizes the antiviral activity of the FAM134B‐mediated reticulophagy, thereby promoting ASFV replication.

**FIGURE 8:**
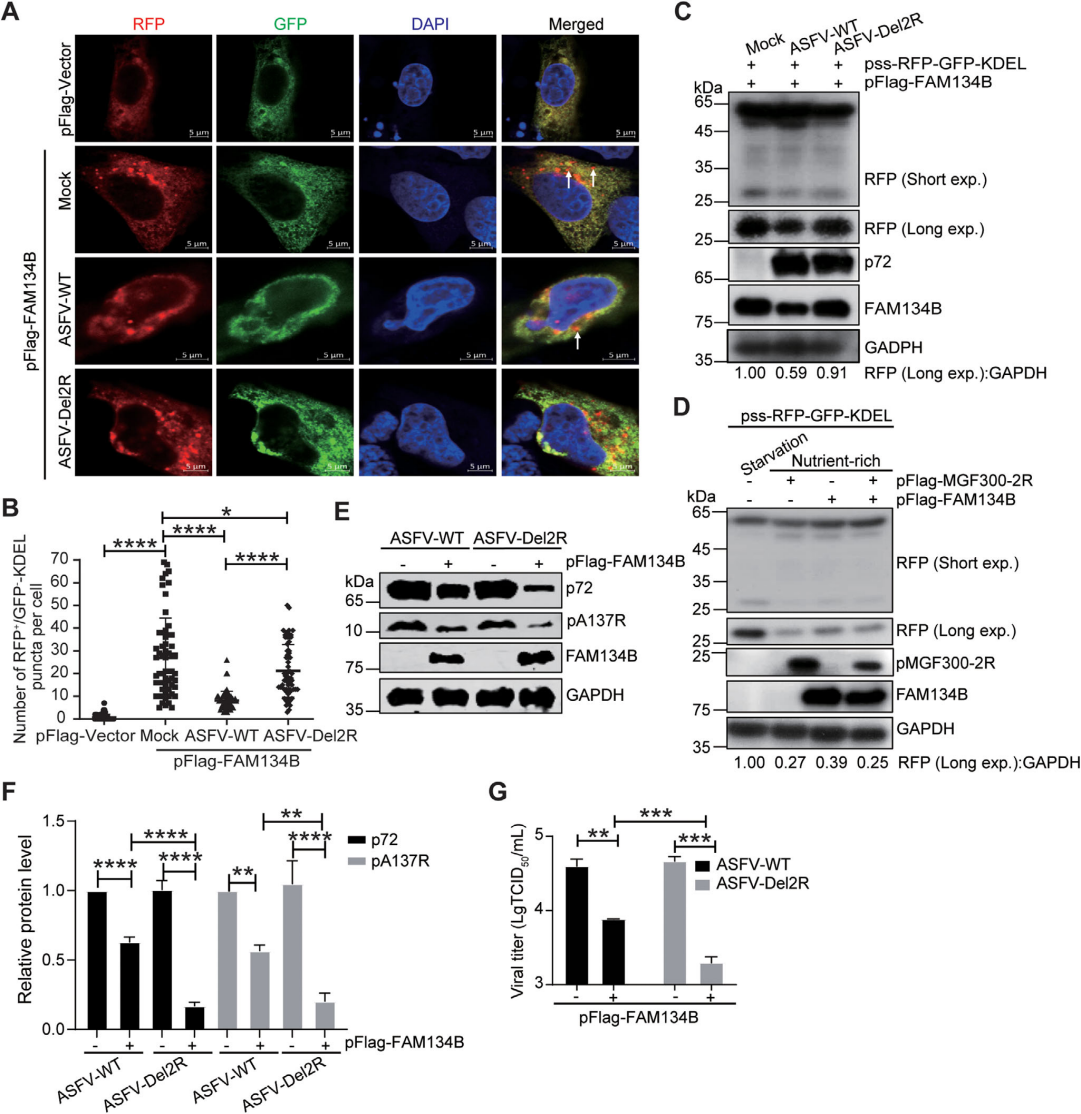
The antiviral effects of FAM134B are antagonized by pMGF300‐2R. (A–D) pMGF300‐2R inhibits the FAM134B‐mediated reticulophagy. WSL cells were cotransfected with pssRFP‐GFP‐KDEL and pFlag‐FAM134B. The cells were infected with either ASFV‐WT (MOI = 1) or ASFV‐Del2R (MOI = 5) at 24 h post‐transfection (hpt). The activation of reticulophagy was analyzed using laser confocal microscopy at 24 h post‐infection (hpi). The white arrowhead represents RFP^+^/GFP^−^ puncta. Scale bars, 5 µm (A). Sixty randomly selected cells were quantitatively analyzed for the number of RFP^+^/GFP^−^ puncta (B). WSL cells were cotransfected with pssRFP‐GFP‐KDEL and pFlag‐FAM134B. At 24 hpt, the cells were infected with ASFV‐WT (MOI = 1) or ASFV‐Del2R (MOI = 5), and immunoblotting analysis was performed at 24 hpi (C). HEK293T cells were cotransfected with pssRFP‐GFP‐KDEL, pFlag‐FAM134B and pFlag‐MGF300‐2R. A starvation treatment group was established as a positive control, and immunoblotting was performed at 24 hpt (D). (E–G) pMGF300‐2R antagonizes the antiviral activity of FAM134B to promote ASFV replication. WSL cells were transfected with pFlag‐FAM134B, followed by infection with either ASFV‐WT (MOI = 1) or ASFV‐Del2R (MOI = 5) at 24 hpt. The cells were subjected to immunoblotting to assess p72 and pA137R expression levels (E and F) and titration of viral titers (G) at 24 hpi. The data are presented as the mean ± SD from three independent experiments. Statistical significance of the data in (B, F, and G) was analyzed by one‐way ANOVA (*p < 0.05; **p < 0.01; ***p < 0.001; ****p < 0.0001).

Our previous study demonstrated that ASFV‐Del2R exhibits significantly reduced pathogenicity in pigs compared with ASFV‐WT [33]. We speculated that the attenuation of ASFV‐Del2R is mediated by the coordinated engagement of multiple host defense mechanisms. To validate this hypothesis, we analyzed the spleen and lung tissues from the uninfected (Mock), ASFV‐Del2R‐infected, and ASFV‐WT‐infected pigs. Hematoxylin and Eosin (H&E) staining confirmed intact splenic architecture with no pathological lesions in the mock and ASFV‐Del2R‐infected pigs, while ASFV‐WT infection induced severe splenic damage, including blurred white‐red pulp boundaries, white pulp atrophy, lymphocyte depletion, and red pulp congestion with hemorrhage (Figure 9A). Moreover, immunohistochemistry (IHC) analysis revealed that there were abundant FAM134B‐positive cells in the spleens of the mock and ASFV‐Del2R‐infected pigs, which were significantly reduced in the ASFV‐WT‐infected animals (Figure 9B,C). We further examined the expression of FAM134B in the spleens by immunoblotting and the results showed that FAM134B protein levels were significantly higher in ASFV‐Del2R‐infected pigs than those in the ASFV‐WT‐infected pigs (Figure 9D,E), which was consistent with the IHC data. These findings suggest that pMGF300‐2R mitigates the antiviral activity of FAM134B.

**FIGURE 9:**
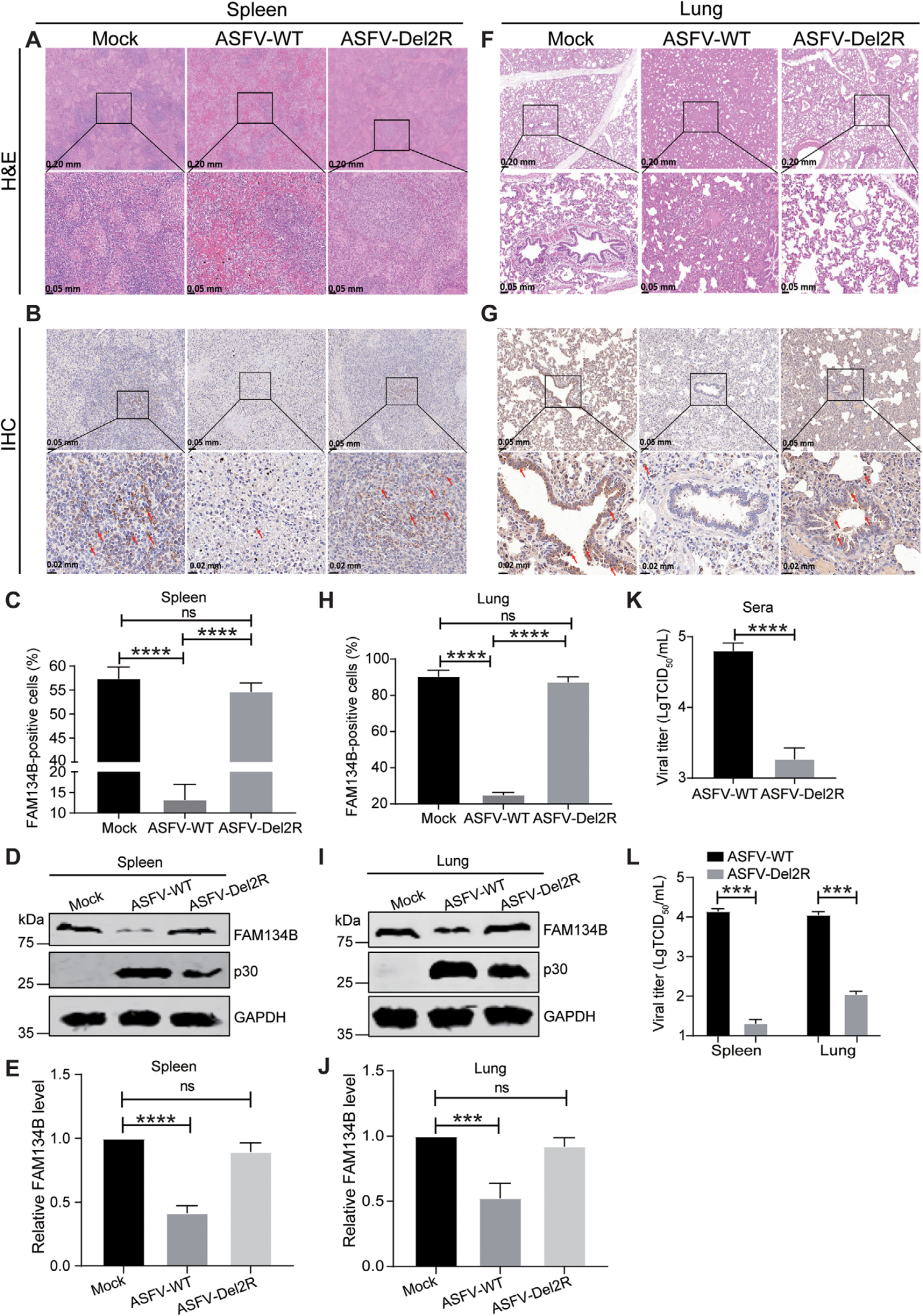
Degradation of FAM134B by pMGF300‐2R facilitates the infectivity and pathogenicity of ASFV. (A–E) Degradation of FAM134B by pMGF300‐2R in the spleens of the ASFV‐infected pigs. Representative images of hematoxylin and eosin (H&E) (A) and immunohistochemistry (IHC) (B) staining for FAM134B (brown) in the spleens from the mock‐, ASFV‐Del2R‐, or ASFV‐WT‐infected pigs. FAM134B‐positive cells in the spleens of the mock‐infected, ASFV‐Del2R‐infected, or ASFV‐WT‐infected pigs were quantified (C). The expression levels of FAM134B in the spleens of the pigs were analyzed using immunoblotting (IB) (D and E). (F–J) Degradation of FAM134B by pMGF300‐2R in the lungs of the ASFV‐infected pigs. Representative images of H&E (F) and IHC (G) staining for FAM134B (brown) in the lungs of the mock‐infected, ASFV‐Del2R‐infected, or ASFV‐WT‐infected pigs. The red arrows indicate cytoplasmic FAM134B‐positive cells. Scale bars: 200 µm for H&E images, 50 µm for magnified H&E images and IHC images, 20 µm for magnified IHC images. FAM134B‐positive cells in the lungs of the mock‐, ASFV‐Del2R‐, or ASFV‐WT‐infected pigs were quantified (H). The expression levels of FAM134B in the lungs of the infected pigs were analyzed using IB (I and J). (K, L) Viral titers in the sera (K), spleens, and lungs (L) were determined. The data are presented as the mean ± SD from three independent experiments. Statistical significance was determined by one‐way ANOVA for the data in panels (C, E, H, and J), and by two‐tailed unpaired *t*‐test for the data in panels (K) and (L) (ns, not significant; ***p < 0.001; ****p < 0.0001).

Next, H&E staining of lung tissues confirmed normal pulmonary histology in mock and ASFV‐Del2R‐infected pigs, whereas ASFV‐WT infection triggered severe diffuse alveolar congestion, edema, and exudative pathological damage (Figure 9F). Consistently, IHC and immunoblotting analyses consistently showed that FAM134B expression was maintained at high levels in the lungs of mock and ASFV‐Del2R‐infected pigs, but was significantly downregulated in ASFV‐WT‐infected animals (Figure 9G–J). Accordingly, viral titers in the sera, spleens, and lungs of the ASFV‐Del2R‐infected pigs were significantly lower than those of the ASFV‐WT‐infected pigs (Figure 9K,L). Furthermore, the RT‐qPCR results revealed that there was no significant difference in FAM134B mRNA levels between the ASFV‐Del2R‐ and ASFV‐WT‐infected pigs (Figure S8A–D). Altogether, these in vivo findings demonstrate that pMGF300‐2R antagonizes the antiviral activity of FAM134B, thereby promoting to the pathogenicity of ASFV.

## 3 Discussion

Reticulophagy, a selective autophagy pathway mediated by receptors such as FAM134B, is critical for maintaining ER homeostasis under various cellular stresses, including pathogen infection [7, 37, 38]. Although FAM134B‐mediated reticulophagy restricts the replication of several RNA viruses, its role in DNA virus infection is largely unexplored. Here, we show that FAM134B inhibits ASFV infection by promoting the degradation of the capsid proteins p72 and pA137R, thereby blocking virion assembly in VFs. In response, ASFV employs its virulence‐associated factor pMGF300‐2R to antagonize this host defense. The ASFV pMGF300‐2R binds FAM134B and, via its C‐terminal LIR3 motif, recruits LC3B to target the lysosomal degradation of FAM134B. Our results reveal a dynamic virus‐host arms race centered on reticulophagy and provide a potential target for antivirals against DNA viruses.

Distinct from herpesviruses, ASFV assembles its icosahedral capsid within VFs, where ER‐derived inner membranes serve as a key docking platform for viral assembly [39]. Although ER disruption has been implicated in ASFV immune evasion [40], the role of ER quality control mechanisms, such as reticulophagy, in DNA virus infections remain unclear. Here, we demonstrate that reticulophagy restricts ASFV replication by reducing p72 abundance (Figure 1H,I). Mechanistically, we identified FAM134B as the reticulophagy receptor responsible for this restriction, mediating the selective degradation of both p72 and pA137R (Figure 2A,B). We further confirmed that FAM134B functioned as an antiviral factor targeting viral capsid proteins, significantly inhibiting the replication of ASFV in HEK293T, WSL cells, and PAMs (Figure 2G–O). Furthermore, FAM134B inhibited viral capsid assembly by blocking p72 oligomerization and disrupting mature virion formation within VFs. Notably, a critical mechanistic question is raised by these observations. Whether p72 oligomerization serves as a potential viral evasion strategy to escape the FAM134B‐mediated reticulophagic clearance remains to be elucidated. It is postulated that p72 oligomeric assembly may sterically hinder the p72‐FAM134B interaction or mask the key motifs required for FAM134B recognition, thereby attenuating reticulophagy‐mediated p72 degradation. This question will be systematically investigated in our future studies. Collectively, these findings establish the FAM134B‐mediated reticulophagy as an intrinsic antiviral defense against ASFV, expanding our understanding of reticulophagy in DNA virus infection.

SARs exist in two distinct forms: soluble SARs, which are recruited to cargo as needed, and transmembrane SARs, which are stably embedded in the membranes of their target organelles [41]. Since FAM134B is classified as a transmembrane SAR, we speculated that its degradation of p72 and pA137R might depend on the reticulophagy it mediates. Co‐IP assay confirmed that FAM134B interacted with both p72 and pA137R (Figure 4A–C). Using inhibitors targeting the proteasomal and autophagy‐lysosomal pathways along with *ATG5*‐KO or *ATG7*‐KO cells, we validated that this degradation was dependent on lysosomal activity (Figure 4D–G). Furthermore, confocal imaging revealed that FAM134B is colocalized not only with p72 and pA137R but also with LC3B and LAMP1 (Figure 4M–P and Figure S5G–J), suggesting that FAM134B facilitates the delivery of these viral proteins to lysosomes for degradation. Recent studies revealed that pA137R interacts with VPS39, an HOPS complex subunit essential for autophagosome‐lysosome fusion. As the FAM134B‐mediated reticulophagy ultimately relies on autophagosome‐lysosome fusion to complete degradation, it remains to be determined whether VPS39 is functionally involved in the FAM134B‐mediated clearance of pA137R [42, 43]. Given that ASFV infection induces ER stress and activates the unfolded protein response [44, 45], a process in which viral proteins, such as pEP152R, pB117L, and pD117L, are implicated [45, 46, 47], we hypothesized that ER stress is a key trigger for FAM134B‐mediated viral protein clearance. We presumed that overexpression of p72 and pA137R might trigger ER stress, thereby marking them for recognition and clearance by FAM134B. Elucidating the precise molecular determinants by which FAM134B selectively recognizes p72 and pA137R remains an important direction for future investigation. While p72 and pA137R are targets, other ASFV proteins may also be degraded by FAM134B‐mediated reticulophagy; future mass spectrometry‐based proteomic comparisons will define the full spectrum of viral substrates and clarify whether this pathway restricts replication beyond virion maturation.

Interestingly, FAM134B protein levels decreased during the late stage of ASFV infection (Figure 5A–D), prompting us to investigate whether viral proteins antagonize the antiviral function of this reticulophagy receptor. Screening of the 61 ASFV‐encoded proteins identified pMGF300‐2R as the sole candidate capable of reducing FAM134B expression (Figure 5F). The pMGF300‐2R, an MGF300 member previously characterized as an ASFV virulence‐associated factor [33, 48, 49], mediates the degradation of IKK*α*/*β* via the autophagy pathway to suppress the NF‐*κ*B signaling pathway [33, 50]. This raised the question of whether a similar mechanism underlies FAM134B downregulation. We found that pMGF300‐2R degraded FAM134B through a lysosome‐dependent pathway (Figure 6F–I). However, unlike IKK*α*/*β* degradation, which requires TOLLIP [33, 50], FAM134B degradation occurred independently of both TOLLIP and p62 (Figure S7A,B), suggesting a distinct molecular mechanism. Recent research indicates that proteins encoded by RNA viruses mimic host short linear protein‐protein interaction motifs, such as LIR, *Ω*XaV, and PPxY motifs, which play a crucial role in viral infection and pathogenicity [34, 35, 51, 52, 53]. We next investigated whether pMGF300‐2R harbors a similar motif to facilitate FAM134B degradation. Interaction analysis indicated that pMGF300‐2R interacted with LC3B (Figure 7B–E), suggesting that pMGF300‐2R may contain an LIR motif. Subsequently, bioinformatics analysis revealed the presence of a canonical LIR motif on the C‐terminus of pMGF300‐2R. Collectively, we discovered that the LIR3 motif of pMGF300‐2R is essential for its interaction with LC3B and the degradation of FAM134B. Furthermore, the use of the *MGF300‐2R* gene‐deficient ASFV mutant confirmed that the autophagic degradation of FAM134B, mediated by pMGF300‐2R, antagonized its antiviral function (Figure 8E–G), thereby facilitating ASFV infection and enhancing pathogenicity. An interesting question that arises from these findings is whether pMGF300‐2R recruits an E3 ubiquitin ligase to ubiquitinate FAM134B, thereby promoting its autophagic degradation. Given that pMGF300‐2R‐mediated FAM134B degradation occurs independently of TOLLIP and p62, it is plausible that an alternative E3 ligase and/or autophagy adaptor is involved. Collectively, this work expands our understanding of viral subversion of selective autophagy and highlights pMGF300‐2R as a multifunctional virulence‐associated factor.

This study has several limitations. First, the exact sites and mechanisms by which FAM134B recognizes p72 and pA137R remain uncharacterized, though we presumed that unfolded capsid proteins accumulated in VFs may serve as recognition signals. Second, while pMGF300‐2R appears to act as a viral autophagy receptor, whether other autophagy adaptors or host molecules are involved remains unknown. Third, although only pMGF300‐2R was identified among 61 ASFV proteins screened, we cannot rule out the possibility that other uncharacterized viral proteins may exert comparable functions.

In summary, our study identifies the FAM134B‐mediated reticulophagy as an intrinsic antiviral defense against ASFV, which restricts viral replication via degrading the capsid proteins p72 and pA137R. We further reveal that ASFV pMGF300‐2R antagonizes this antiviral effect by inducing autophagic degradation of FAM134B, which unveils the complex and dynamic interplay between ASFV and reticulophagy. Collectively, these findings establish a mechanistic framework and identify potential molecular targets for the development of reticulophagy‐oriented anti‐ASFV strategies (Figure 10).

**FIGURE 10:**
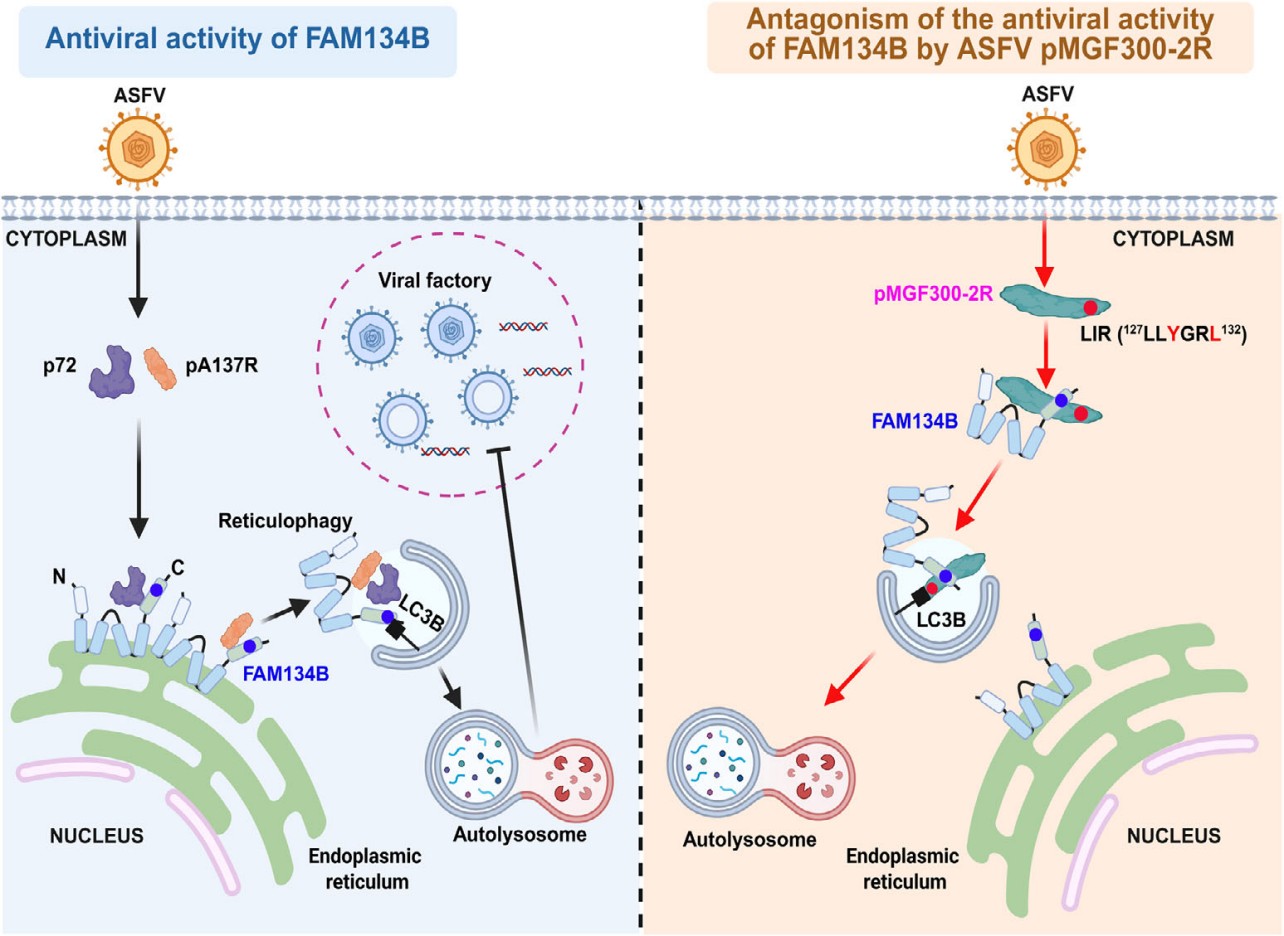
A working model depicting how the ASFV pMGF300‐2R counteracts the FAM134B‐mediated antiviral response. FAM134B inhibits the ASFV capsid assembly by promoting the degradation of the capsid proteins p72 and pA137R via reticulophagy (left side). In contrast, the antiviral activity of FAM134B is antagonized by the ASFV virulence‐associated protein pMGF300‐2R (right side).

## 4 Experimental Section

### 4.1 Ethics Statements

All experiments involving live ASFV manipulation in this study were carried out at the biosafety level 3 (BSL‐3) facilities of the Harbin Veterinary Research Institute (HVRI), under the auspices of the Chinese Academy of Agricultural Sciences (CAAS). These experiments were conducted in compliance with strict safety protocols and regulations and received approval from the Ministry of Agriculture and Rural Affairs of China.

### 4.2 Cells and Viruses

Human embryonic kidney 293T (HEK293T, RRID: CVCL_0063) cells were obtained from the American Type Culture Collection (ATCC, Manassas, VA, USA). Wild boar lung (WSL) cells were generously provided by Professor Jun Han at China Agricultural University [54]. Primary porcine alveolar macrophages (PAMs) were isolated from the lung lavage fluid of 28‐day‐old healthy specific‐pathogen‐free (SPF) piglets. *ATG5*‐KO, *ATG7*‐KO, *p62*‐KO, *TOLLIP*‐KO, or *FAM134B*‐KO HEK293T cells were previously reported [11, 33]. All cells used in this study were authenticated using short‐tandem‐repeat (STR) profiling and confirmed to be mycoplasma‐free using a cell‐staining assay kit (Beyotime, C0296). HEK293T cells were cultured in Dulbecco's Modified Eagle's Medium (DMEM) (Gibco, C11995500BT) supplemented with 10% fetal bovine serum (FBS, Gibco, A5669701). PAMs and WSL cells were maintained in RPMI‐1640 medium with L‐glutamine (Thermo Fisher Scientific, C11875500BT) supplemented with 10% (v/v) FBS and antibiotics (100 U/mL penicillin and 100 µg/mL streptomycin). *FAM134B*‐KO WSL cells were generated using the CRISPR/Cas9 system with sgRNA (Table S2) inserted into the pX458 vector, as described previously [55]. All cells were maintained in an incubator with 5% CO_2_ at 37°C.

The virulent ASFV HLJ/18 strain (ASFV‐WT) (GenBank accession no. MK333180.1) was isolated as described previously [33]. The HEK293T cell‐adapted ASFV strain (ASFV‐P31) and MGF300‐2R‐deleted ASFV mutant (ASFV‐Del2R) were generated as described previously [14, 33].

### 4.3 Antibodies and Reagents

Mouse anti‐His (AE003), rabbit anti‐*β*‐tubulin (A12289), mouse anti‐GST (AE001), anti‐Flag (AE005), anti‐HA (AE008), anti‐ glyceraldehyde‐3‐phosphate dehydrogenase (GAPDH) (AC033), and anti‐GFP (AE012) and rabbit anti‐HA (AE105) antibodies were purchased from ABclonal Biotechnology Co., Ltd. Rabbit anti‐RFP (BGT‐ANT‐45233) antibodies were purchased from Biogradetech. Rabbit anti‐FAM134B (21537‐1‐AP), anti‐LC3 (14600‐1‐AP), and anti‐Vimentin (60330‐1‐Ig) antibodies were purchased from Proteintech. Rabbit anti‐GSDMD (ab219800) and anti‐cleaved N‐terminal GSDMD (ab215203) were purchased from Abcam. Horseradish peroxidase (HRP)‐conjugated goat anti‐rabbit IgG (111‐035‐003) and goat anti‐mouse IgG (115‐035‐003) antibodies were purchased from Jackson ImmunoResearch. Alexa Fluor 488‐conjugated goat anti‐rabbit IgG (H+L) (A11008) and anti‐mouse IgG (H+L) (A11029), Alexa Fluor 647‐conjugated donkey anti‐rabbit IgG (H+L) (A31573) and anti‐mouse IgG (H+L) (A31571), Li‐Cor IRDye 800CW donkey anti‐mouse IgG (H+L) (926‐32212) and goat anti‐rabbit IgG (H+L) (926‐32211) antibodies were purchased from Thermo Fisher Scientific. Alexa Fluor 594 donkey anti‐rabbit IgG (minimal x‐reactivity) antibody (406418) was purchased from Biolegend. The anti‐HA agarose (A2095) and anti‐Flag(R) M2 magnetic beads (M8823) were purchased from Sigma‐Aldrich. The anti‐A137R, ‐p72, ‐p30, and ‐p54 polyclonal antibodies were prepared as described previously [33].4’,6‐Diamidino‐2‐phenylindole (DAPI) (C006) was purchased from Solarbio. Bafilomycin A1 (BafA1) (sc‐201550A) was purchased from Santa Cruz Biotechnology. Lactacystin (L6785), MG132 (474790), and Earle's balanced salt solution (EBSS) (E2888) were purchased from Sigma‐Aldrich. CQ (HY‐17589A), Protein A/G Magnetic Beads (HY‐K0202), and Ac‐YVAD‐cmk (HY‐16990) were purchased from MedChemExpress. The high glucose D‐MEM with sodium pyruvate, without amino acids was purchased from Wako (048‐33575). Disuccinimidyl suberate crosslinker (DSS) was purchased from Thermo Fisher Scientific (A39267).

### 4.4 Plasmid Construction and Transfection

The ssRFP‐GFP‐KDEL sequence of the reticulophagy reporter was synthesized by AZENTA (Suzhou, China) and cloned into the pCMV‐HA vector (pssRFP‐GFP‐KDEL). The porcine *FAM134B* gene (GenBank accession no. XM_003483804) was synthesized by AZENTA and cloned into the pCMV‐HA, pCMV‐Flag, or pGEX‐6P‐1 vector, resulting in pHA‐FAM134B, pFlag‐FAM134B, and pGST‐FAM134B, respectively. The plasmids, including pFlag‐RTN3, pFlag‐ATL3, pFlag‐CCPG1, pFlag‐SEC62, pHA‐MGF300‐2R, pFlag‐MGF300‐2R, pEGFP‐MGF300‐2R, pHis‐MGF300‐2R, pEGFP‐LC3B, and pmCherry‐LAMP1, were described previously [33, 55]. The FAM134B‐, MGF300‐2R‐, and LC3B‐derived mutants were generated by site‐directed mutagenesis on the basis of their respective wild‐type constructs. The primers used in this study are listed in Table S2. The genes encoding ASFV proteins (Table S3) were cloned into the pCAGGS vector. All the plasmids used in this study were verified by sequencing.

Polyethyleneimine (PEI) (Polysciences, 23966‐2) was used to transfect HEK293T cells. In brief, the transfection mixture contained 3 µg of the specified plasmid and 9 µL of PEI (1 mg/mL) stock solution diluted in 200 µL of Opti‐MEM (Invitrogen, 31985070). After a 30‐min incubation at room temperature, the transfection mixture was added to the HEK293T cells, which were then incubated for 24 to 36 h. PAMs and WSL cells were transfected using the X‐tremeGENE HP reagent (Roche, 6366546001) following the manufacturer's instructions.

### 4.5 Viral Infection

PAMs, WSL, or HEK293T cells were infected with ASFV at the indicated MOI. Virus samples were harvested and titrated at the designated time points as described previously [33]. For the Western blotting assay, the cell lysates were harvested at the indicated time points for determination.

### 4.6 Lentiviral Transduction

The ORFs of *FAM134B* and *FAM134B‐ΔLIR* were cloned into the lentiviral vector pLVX‐IRES‐ZsGreen1 (pLVX‐FAM134B or (pLVX‐FAM134B‐ΔLIR). Lentiviruses were packaged by cotransfection of HEK293T cells with pLVX‐FAM134B (or pLVX‐FAM134B‐ΔLIR), pMD2.G, and psPAX2. *FAM134*‐KO HEK293T cells were transduced with the resulting recombinant lentivirus at a MOI of 0.3, and ZsGreen‐positive cells were selected by flow cytometry at three days post‐transduction.

### 4.7 Laser Confocal Microscopy

PAMs, HEK293T, and WSL cells were cultivated in glass‐bottom culture dishes (NEST, 801001) and transfected with the indicated expression plasmids using X‐tremeGENE HP and then infected with ASFV. Subsequently, the cells were fixed with paraformaldehyde solution (4%) at room temperature for 30 min, and then washed three times with phosphate‐buffered saline (PBS). Then, the transfected cells were subjected to a 30‐min permeabilization with 0.1% triton X‐100 (Sigma‐Aldrich, V900502) in PBS. Following a block with 5% bovine serum albumin (BSA), the cells were incubated with the specific primary antibodies at 37°C for 2 h. After five washes with PBS, the cells were stained with fluorescence‐conjugated secondary antibodies at 37°C for 1 h. Finally, the cells were treated with DAPI at room temperature for 10 min and then observed using a laser confocal microscope (Zeiss, LSM880).

### 4.8 Immunoprecipitation‐Mass Spectrometry (IP‐MS)

WSL cells were transfected with either pHA‐FAM134B or pHA‐Vector. At 24 h post‐transfection (hpt), the cells were infected with ASFV‐WT, and the lysates were immunoprecipitated with anti‐HA magnetic beads at 24 hpi. The eluates were subjected to IP‐MS analysis at BGI Group (Shenzhen). The peptide mixtures were analyzed using a Q Exactive mass spectrometer (Thermo Fisher). The resulting RAW files were processed with the Proteome Discoverer software, and protein identification was performed by searching against the ASFV protein database (GenBank accession no. MK333180.1). Proteins were identified based on at least three unique peptides, with a protein score threshold of ≥20, and the confidence thresholds for protein and peptide identification were set to a 1% false discovery rate. The mass spectrometry‐based proteomics data have been deposited in the ProteomeXchange Consortium (https://proteomecentral.proteomexchange.org) via the iProX partner repository [56, 57] with the dataset identifier PXD074277.

### 4.9 RNA Interference (RNAi) Assay

Small interference RNAs (siRNAs) specifically targeting the *FAM134B* gene and a scrambled siRNA control were designed and synthesized by GenePharma (Shanghai, China) (Table S2). For siRNA transfection, 100 pmol of the siRNAs were transfected into PAMs using the Lipofectamine RNAiMAX transfection reagent (Thermo Fisher Scientific, 13778030) according to the manufacturer's instructions. The protein expression was analyzed by Western blotting.

### 4.10 Transmission Electron Microscopy (TEM)

WSL cells were transfected with pHA‐FAM134B and subsequently infected with ASFV‐WT at an MOI of 5. At 12 hpi, the cells were fixed with 2% glutaraldehyde and prepared for TEM following the established protocols [33].

### 4.11 Western Blotting

The transfected or infected cells were lysed in pre‐cooled RIPA lysis buffer (Thermo Fisher Scientific, 89901) supplemented with a protease inhibitor (Solarbio, A8260‐5 mg). Subsequently, the resulting cell lysates were subjected to centrifugation at 12,000 × *g* for 10 min at 4°C. The total proteins from the cell extracts were separated using sodium dodecyl sulfate‐polyacrylamide gel electrophoresis (SDS‐PAGE) and subsequently transferred to polyvinylidene difluoride (PVDF) membranes. After successive incubation with primary and secondary antibodies, the membranes were visualized using an Odyssey two‐color infrared fluorescence imaging system (Li‐Cor). For chemiluminescence detection, the membranes were visualized using an enhanced chemiluminescence (ECL) substrate (Thermo Fisher Scientific, 32106).

Gray value analyses were conducted using the ImageJ software (https://imagej.nih.gov/ij/). Brightness and contrast adjustments were applied uniformly to all the images within the same panel. The levels of target proteins were normalized to those of GAPDH or β‐tubulin, and the results were presented as relative values, with the control vector set to 1.

### 4.12 Co‐Immunoprecipitation (Co‐IP) Assay

The transfected or infected cells in 60‐mm dishes were treated with 1 mL of RIPA lysis buffer for 30 min at 4°C. The lysates were harvested, 150 µL of which was used for analysis by Western blotting, and the remaining 850 µL of the lysates were then incubated with the corresponding primary antibody. Thereafter, anti‐HA agarose, anti‐Flag M2, or anti‐IgG magnetic beads were added, and the mixture was gently rotated overnight at 4°C. To isolate the HA‐ or Flag‐tagged target proteins, the lysates were incubated with anti‐HA agarose or anti‐Flag M2 magnetic beads and rotated overnight. The agarose or beads were harvested and washed five times with pre‐cooled RIPA lysis buffer. Subsequently, the proteins were extracted from the beads by boiling in a mixture of 80 µL of RIPA lysis buffer and 20 µL of 5 × sample loading buffer, followed by Western blotting analysis.

### 4.13 GST Pulldown Assay

The expression and purification of GST empty vector (GST), GST‐FAM134B, GST‐MGF300‐2R, GST‐MGF300‐2R‐ΔLIR3, GST‐MGF300‐2R^Y129A^, GST‐MGF300‐2R^L132A^, GST‐MGF300‐2R‐D2, GST‐LC3B, GST‐LC3B^K49A/K51A^, His‐MGF300‐2R and His‐MGF300‐2R‐ΔLIR3 proteins were performed using *E. coli* BL21(DE3) competent cells (Weidi Biotechnology, EC2113). For the in vitro GST pulldown assay, GST (serving as a negative control), GST‐FAM134B, GST‐LC3B or GST‐LC3B^K49A/K51A^ was mixed with prepared glutathione‐sepharose beads (GE Healthcare, 17‐0756‐01) and incubated for 12 h at 4°C, followed by 5 washes with PBS. The bead‐protein complexes were incubated with His‐MGF300‐2R protein at 4°C for 12 h, followed by 10 washes with PBS. The bead‐protein complexes conjugated with GST or GST‐LC3B were incubated with His‐MGF300‐2R or His‐MGF300‐2R‐ΔLIR3 protein at 4°C for 12 h, followed by 10 washes with PBS. Subsequently, the obtained complexes were heated in SDS loading buffer for 10 min, followed by separation via SDS‐PAGE and immunoblotting analysis.

For the semi‐in vitro GST pulldown assay, GST empty vector, GST‐MGF300‐2R, GST‐MGF300‐2R‐ΔLIR3, GST‐MGF300‐2R^Y129A^, GST‐MGF300‐2R^L132A^, or GST‐MGF300‐2R‐D2 was individually mixed with the prepared glutathione‐sepharose beads, followed by incubation for 12 h at 4°C and 10 washes with PBS. Next, the bead‐protein complexes were incubated with the whole‐cell lysates prepared from HEK293T cells overexpressing LC3B‐EGFP. The subsequent experimental procedures were performed as described above for the GST pulldown assay.

### 4.14 RNA Isolation and Quantitative Reverse Transcription‐PCR (RT‐qPCR)

Total RNAs from PAMs were isolated using a cell total RNA isolation kit (BioFlux, BSC52M1), and cDNAs were synthesized with FastKing gDNA Dispelling RT SuperMix (Tiangen, KR118‐02). The resulting cDNAs served as the template for RT‐qPCR using SYBR Premix Ex Taq (TaKaRa, RR390B) to assess the expression levels of the *CP204L* (p30) and *FAM134B* genes. *GAPDH* was used as a reference for normalization. The relative abundance of each gene was calculated using the comparative cycle threshold (2^−ΔΔCT^) method [33].

### 4.15 Animal Experimentation

Pig inoculation experiments were conducted as described previously [33]. In the present study, the spleens and lungs from the ASFV‐WT (10^3.0^ TCID_50_/piglet, *n* = 3), ASFV‐Del2R (10^3.0^ TCID_50_/piglet, *n* = 4), and mock (*n* = 3) groups were used for H&E staining and IHC analysis, as these are target organs of ASFV with high viral loads. The animal experimental protocols were approved by the Institutional Animal Care and Use Committee (IACUC) of HVRI (Authorization number: 221128‐01‐GJ).

### 4.16 H&E Staining and IHC

The spleens and lungs were fixed in 4% (w/v) polyformaldehyde, embedded in paraffin, and sectioned into 5‐µm‐thick slices. For H&E staining, the paraffin sections were subjected to deparaffinization and rehydration prior to H&E staining. For IHC analysis, deparaffinized and rehydrated sections were subjected to antigen retrieval in 10 mM citrate buffer (pH 6.0) via microwave treatment for 10 min. Endogenous peroxidase activity was blocked with 3% hydrogen peroxide (H_2_O_2_), followed by rinsing with PBS. After blocking with 3% BSA at room temperature for 30 min, the sections were incubated overnight at 4°C with primary antibodies targeting FAM134B. Subsequent to PBS washing, the sections were incubated with HRP‐conjugated secondary antibodies for 1 h at room temperature and then rinsed again with PBS. Finally, the sections were stained with 3,3’‐diaminobenzidine (DAB), counterstained with hematoxylin, and quantitative analysis was performed using the Saiviewer software.

### 4.17 Cell Viability Assay

The cells were seeded in 96‐well plates at a density of 2000 cells per well in 100 µL of medium. Subsequently, cell viability was assessed using the Promega CellTiter‐Glo luminescent cell viability assay kit (Promega, G7572).

### 4.18 In Silico Identification of Functional LIRs

The iLIR@viral (https://ilir.warwick.ac.uk/search.php) with default parameters [58] was used to predict the LIR motifs on pMGF300‐2R.

### 4.19 Statistical Analysis

All the data were presented as the mean ± standard deviation (SD) and analyzed using the GraphPad Prism software (Version 9.5.1). The two‐tailed unpaired Student's *t*‐test was applied to the data of two groups, and one‐way ANOVA analyses were used for comparisons of the data of more than two groups. Sample sizes were indicated in the figure legends. A *p*‐value < 0.05 was considered statistically significant.

## Author Contributions

R.L., J.Z., and T.W. conceived the study conception and design. R.L., J.Z., R.H., J.L., Z.L., X.Z., L.F.L., and T.W. performed material preparation, data collection, and analysis. Y.S., T.W., and H.J.Q. contributed to the supervision and project administration of the study. Y.S., J.Z., T.W., and H.J.Q. contributed to the funding acquisition and resources of the study. R.L., J.Z., and T.W. wrote the manuscript draft. All authors read and approved the final manuscript.

## Conflicts of Interest

The authors declare no conflicts of interest.

## Supporting information




**Supporting File**: advs77741‐sup‐0001‐SuppMat.pdf.

## Data Availability

The data that supports the findings of this study are available in the Supporting Information of this article.
