# Supplementary material for: FAM134B Restricts African Swine Fever Virus Capsid Assembly via Reticulophagy and Its Antiviral Activity is Antagonized by the Viral Virulence‐Associated Factor pMGF300‐2R
Source: Adv Sci (Weinh). 2026 Sep 16;13(58):e77741. doi: 10.1002/advs.77741 (PMC13583106; doi:10.1002/advs.77741)

FAM134B Restricts African Swine Fever Virus Capsid Assembly via Reticulophagy and Its Antiviral Activity Is Antagonized by the Viral Virulence-Associated Factor pMGF300-2R

Rui Luo[#], *Jing Zhang*[#], *Ruojia Huang*[#], *Xianfeng Zhang*[#], *Lian-Feng Li*, *Jing Lan*, *Zhanhao Lu*, *Yuan Sun**, *Tao Wang**, *Hua-Ji Qiu**

State Key Laboratory of Animal Disease Control and Prevention, Professional Laboratory for African Swine Fever (Harbin), National High Containment Facilities for Animal Disease Control and Prevention, Harbin Veterinary Research Institute, Chinese Academy of Agricultural Sciences, Harbin, China

[#]Rui Luo, Jing Zhang, Ruojia Huang, and Xianfeng Zhang contributed equally to this work.

***Contact**: qiuhuaji@caas.cn (Hua-Ji Qiu); wangtao07@caas.cn (Tao Wang), and sunyuan@caas.cn (Yuan Sun)

Fig. S1

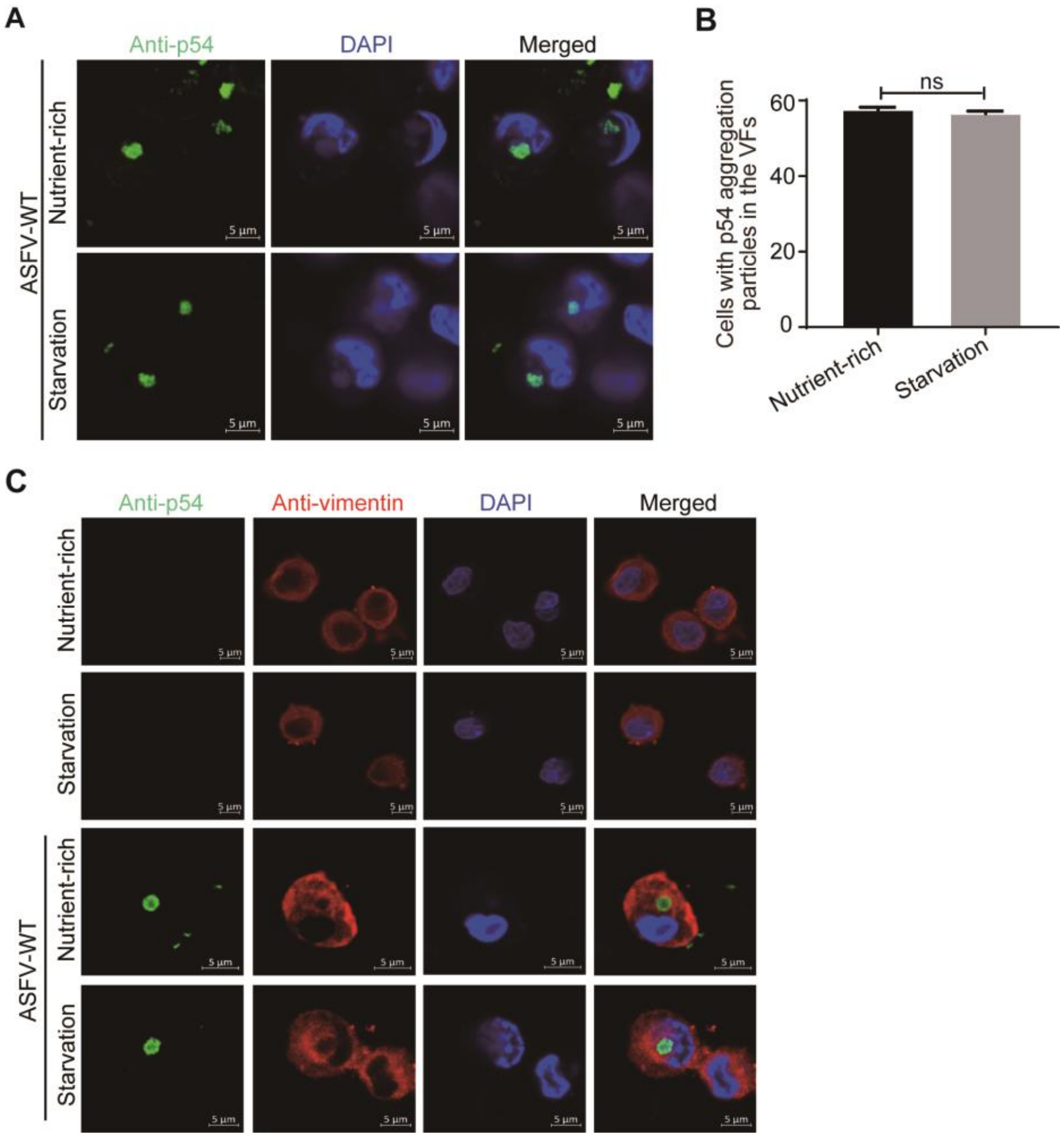

Figure S1. The distribution of p54 and vimentin in ASFV-infected primary porcine alveolar macrophages (PAMs). PAMs were subjected to a 12-h starvation using high-glucose D-MEM medium, which contained sodium salts and lacked amino acids. Subsequently, the cells were infected with ASFV-WT at a multiplicity of infection (MOI) of 1. At 12 hours postinfection, the cells were labeled with anti-p54 (**A** and **B**) or anti-vimentin (**C**) antibodies. Scale bars, 5 μm. The cells with p54 aggregate particles in viral factories (VFs) were quantified for each group (*n* = 60 cells per group) (**B**). The data are presented as the mean ± SD from three independent experiments. Statistical significance was analyzed by two-tailed unpaired *t* test (ns, not significant).

Fig. S2

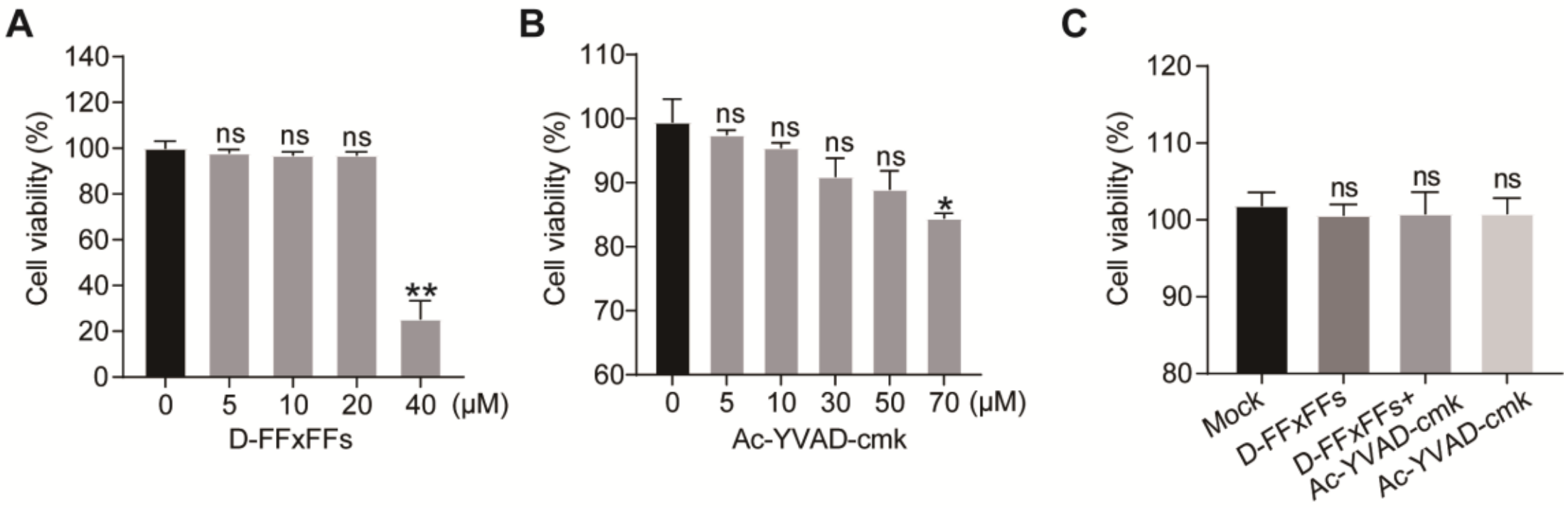

Figure S2. Cell viability of primary porcine alveolar macrophages (PAMs) treated with D-FFxFFs or Ac-YVAD-cmk. Following a 24-h treatment of PAMs with varying concentrations of short peptides (D-FFxFFs) (**A**) or Ac-YVAD-cmk (**B** and **C**), the cell viability was assessed by CellTiter-Glo luminescent cell viability assay kit. The data are presented as the mean ± SD from three independent experiments. Statistical significance was analyzed by one-way ANOVA (ns, not significant; *P < 0.05; **P < 0.01).

Fig. S3

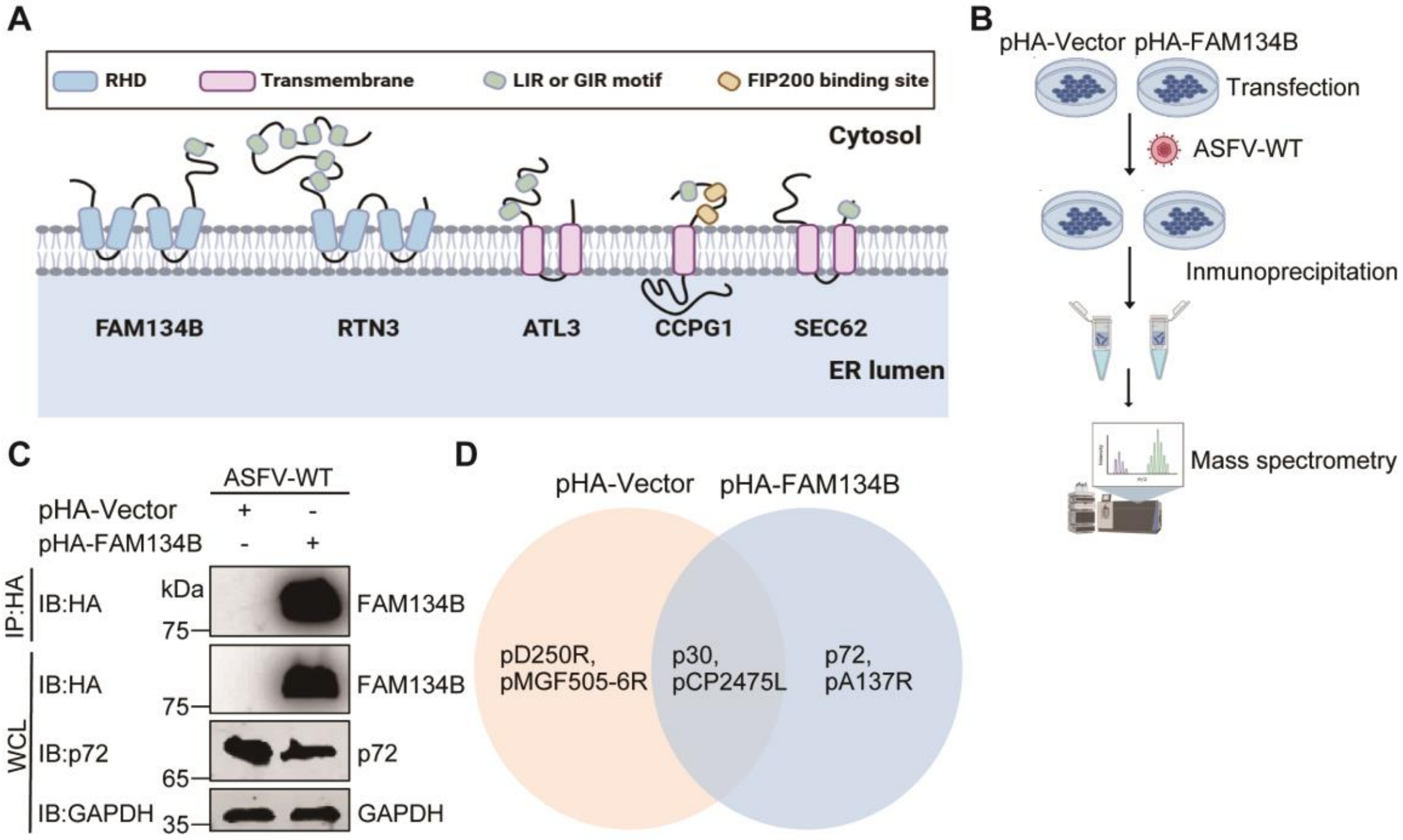

Figure S3. Identification of ASFV proteins interacting with FAM134B. Schematic of the reticulophagy receptors (**A**). Schematic of the experimental strategy (**B**). WSL cells were transfected with pHA-FAM134B and infected with ASFV-WT at a multiplicity of infection (MOI) of 1 at 24 hours posttransfection. At 24 hours postinfection, the samples were subjected to co-immunoprecipitation assay followed by immunoblotting analysis (**C**). The Venn diagram illustrates the viral proteins interacting with FAM134B (**D**).

Fig. S4

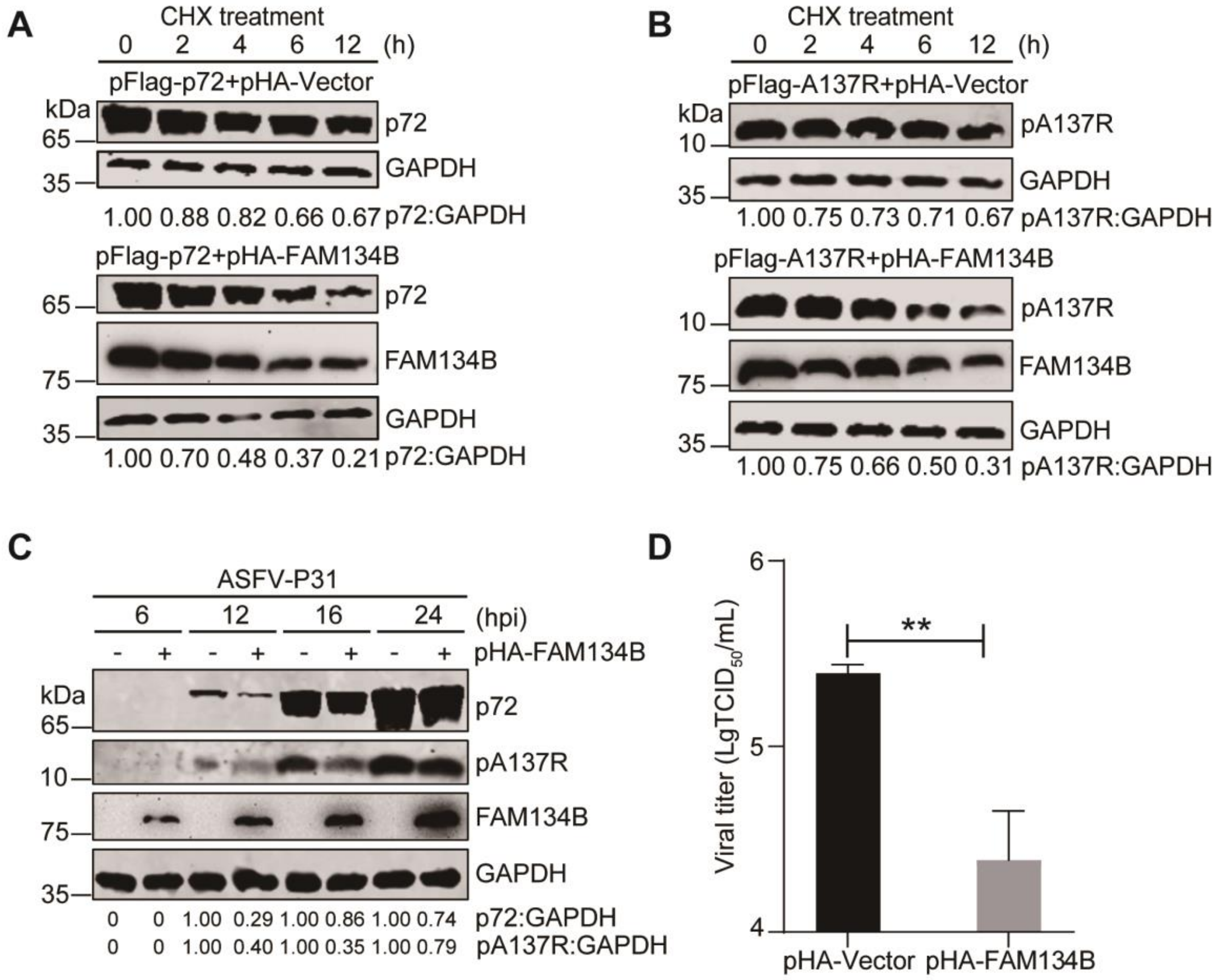

Figure S4. Degradation kinetics analysis of ASFV p72 and pA137R. HEK293T cells were cotransfected with either pHA-Vector or pHA-FAM134B, along with pFlag-p72 (**A**) or pFlag-A137R (**B**). Following transfection, the cells were treated with 100 µg/mL of CHX and harvested at the indicated time points. The cell lysates were analyzed by immunoblotting (IB). HEK293T cells were transfected with the pHA-FAM134B. The cells were then infected with ASFV-P31 at a multiplicity of infection (MOI) of 1. The samples were subjected to IB at the indicated time points to assess the expression levels of p72 and pA137R (**C**). Concurrently, viral titers were quantified at 24 hours postinfection (**D**). The data are presented as the mean ± SD from three independent experiments. Statistical significance was performed using two-tailed unpaired *t* test (***P* < 0.01).

Fig. S5

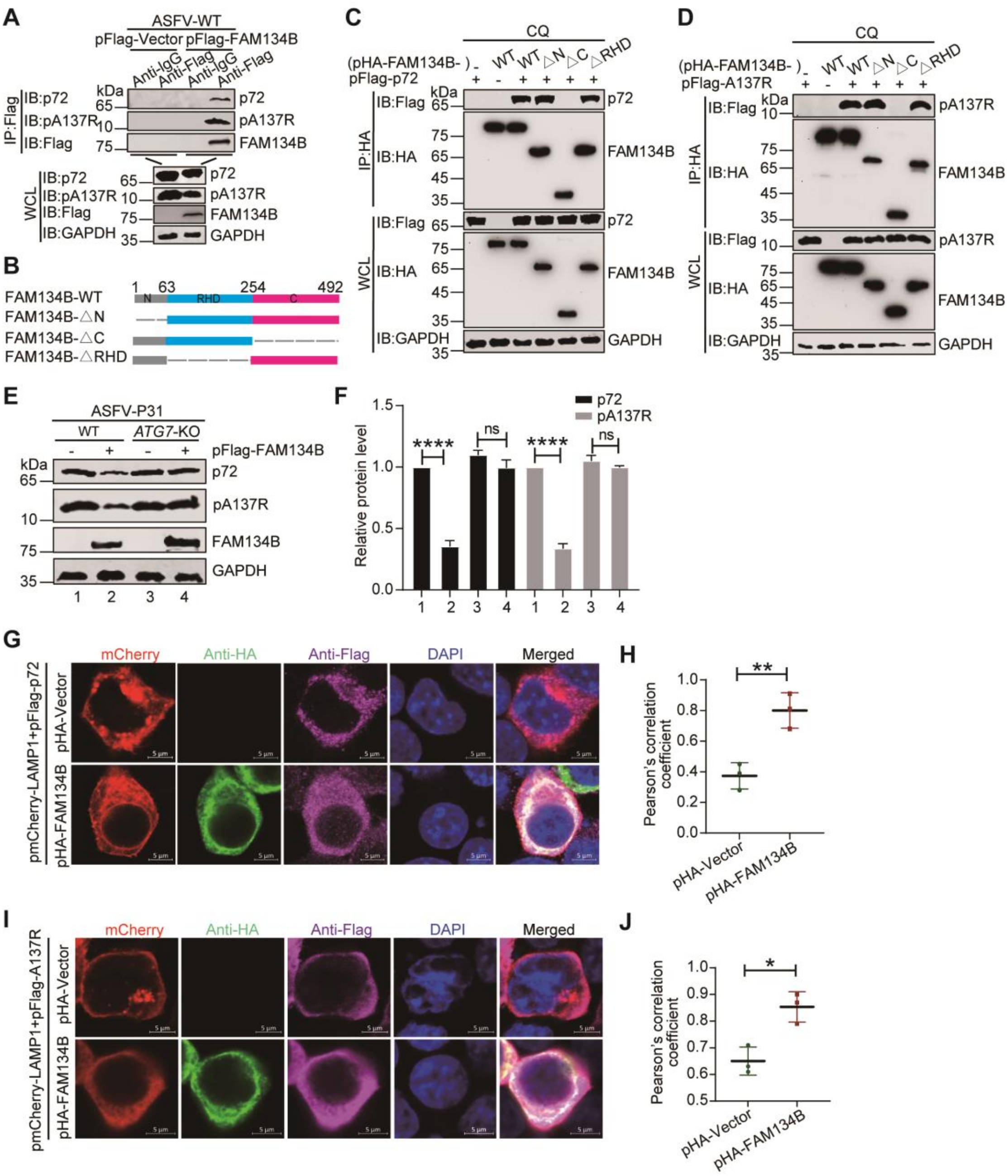

Figure S5. The autophagic degradation of ASFV capsid proteins p72 and pA137R was mediated by FAM134B. WSL cells were either transfected with pFlag-FAM134B, followed by infection with ASFV-WT at a multiplicity of infection (MOI) of 1. At 24 hours postinfection (hpi), cells were lysed for immunoprecipitation (IP) with anti-Flag antibodies. Anti-IgG antibodies were used as a negative control (**A**). Schematic representation of truncated FAM134B mutants (**B**). The interactions between various truncated forms of FAM134B and p72 (**C**) or pA137R (**D**) were analyzed by

co-immunoprecipitation (Co-IP) assay. The *ATG7*-knockout (*ATG7*-KO), or wild-type (WT) HEK293T cells were transfected with pFlag-FAM134B, followed by infection with ASFV-P31 at an MOI of 1. At 24 hpi, the expression levels of p72, pA137R, and FAM134B were analyzed by IB (**E** and **F**). (**G** to **J**) p72 or pA137R is colocalized with FAM134B and LAMP1. HEK293T cells were cotransfected with either pHA-Vector or pHA-FAM134B, along with pmCherry-LAMP1 and pFlag-p72 (**G** and **H**), or cotransfected with either pHA-Vector or pHA-FAM134B, along with pmCherry-LAMP1 and pFlag-A137R (**I** and **J**). Colocalization of FAM134B, p72, pA137R, and LAMP1 were analyzed by laser confocal microscopy. Scale bars, 5 μm. The data are presented as the mean ± SD from three independent experiments. Statistical significance was determined by one-way ANOVA for the data in panel **F**, and by two-tailed unpaired *t* test for the data in panels **H** and **J** (ns, not significant; *$P < 0.05$; **$P < 0.01$; ****$P < 0.0001$).

Fig. S6

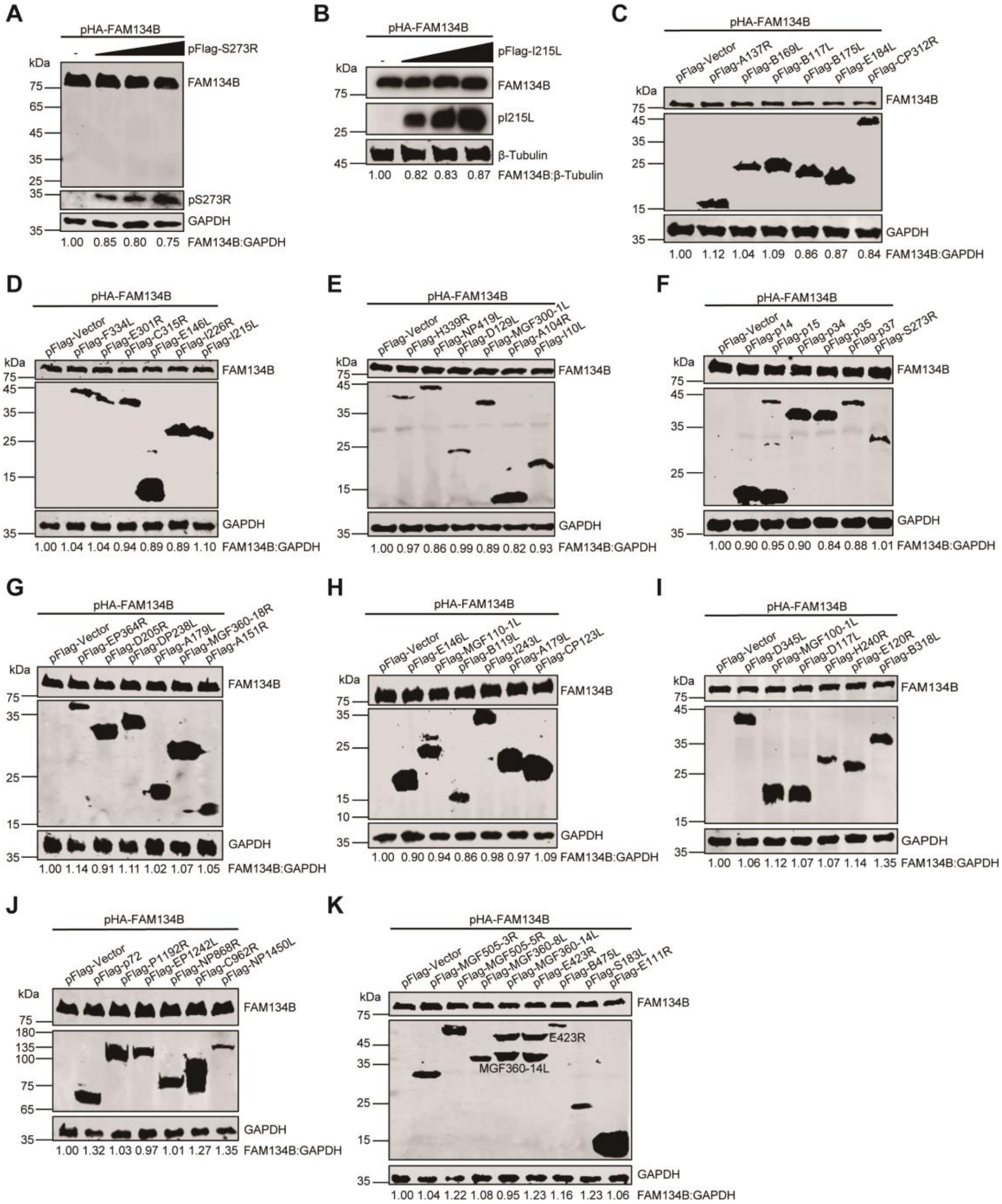

Figure S6. Identification of viral proteins regulating FAM134B expression using the ASFV ORF library. HEK293T cells were cotransfected with an increasing amount (0.5, 1.0, and 1.5 µg) of pFlag-S273R (**A**) or pFlag-I215L (**B**) and pHA-FAM134B. At 24 hours posttransfection (hpt), FAM134B expression was analyzed by immunoblotting. HEK293T cells were cotransfected with the plasmids encoding ASFV proteins and FAM134B. At 24 hpt, FAM134B expression was analyzed by immunoblotting (**C** to **K**).

Fig. S7

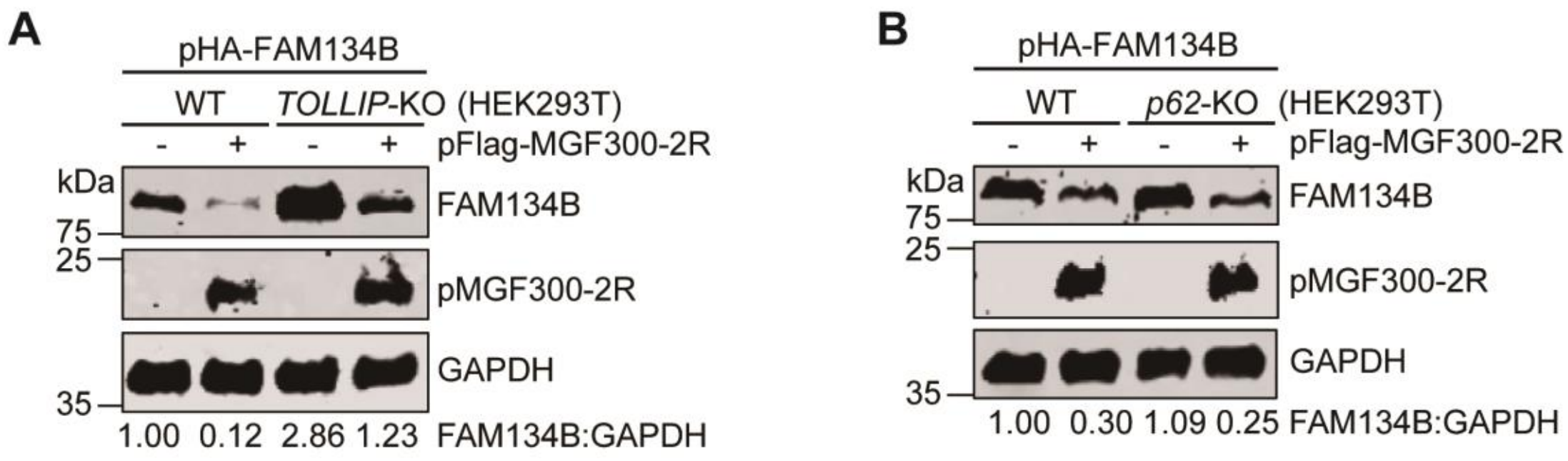

Figure S7. Knockout of *TOLLIP* and *p62* does not affect the degradation of FAM134B by pMGF300-2R. *TOLLIP*- (**A**) or *p62*- (**B**) knockout HEK293T cells were cotransfected with pHA-FAM134B and pFlag-MGF300-2R. At 24 hours posttransfection, the expression of FAM134B was examined by immunoblotting.

Fig. S8

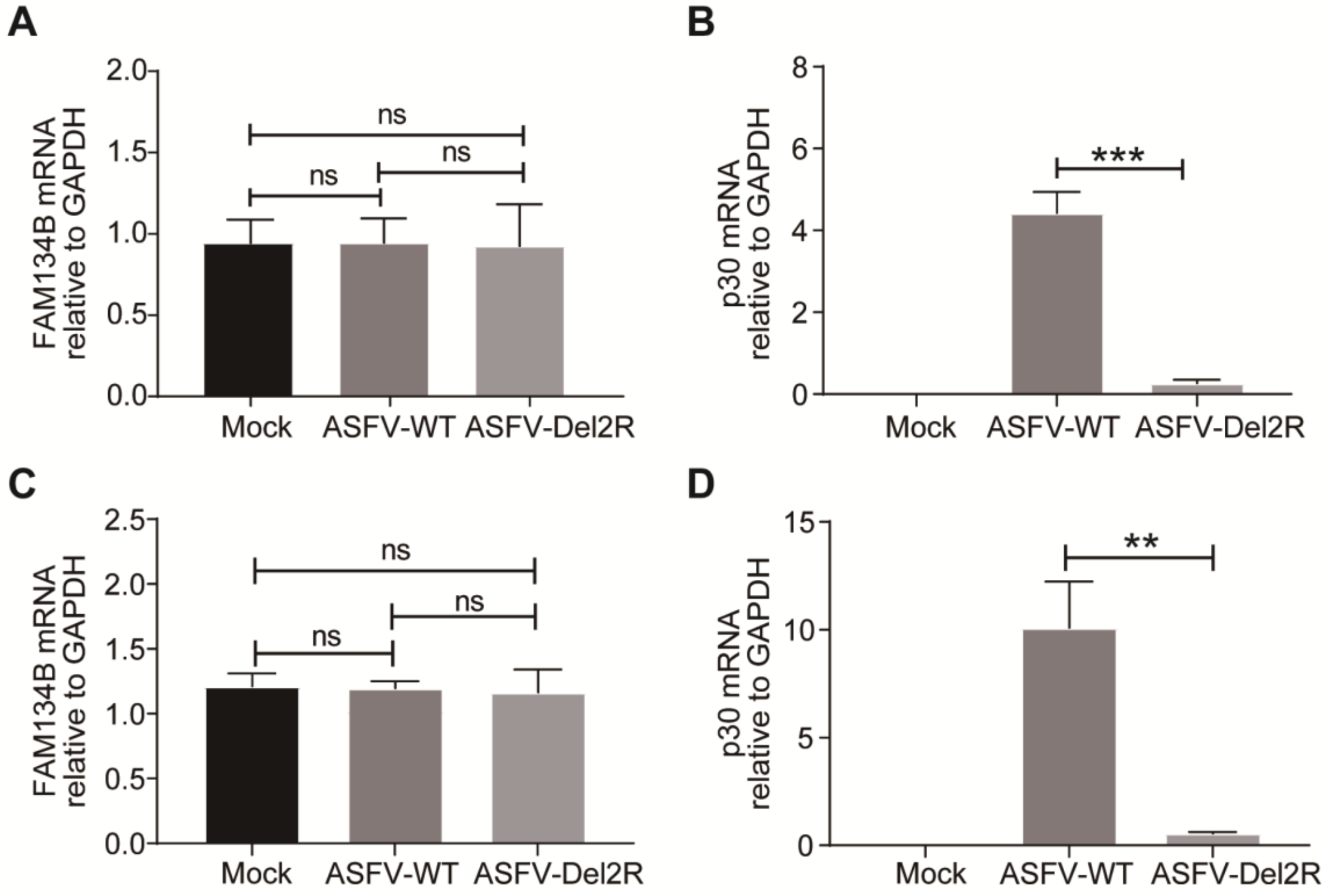

Figure S8. *FAM134B* expression levels in the spleens and lungs of the ASFV-WT- or ASFV-Del2R-infected pigs. RT-qPCR was employed to quantify the expression of *FAM134B* in the spleens (**A**) and lungs (**C**) and that of p30 in the spleens (**B**) and lungs (**D**) from the pigs infected with ASFV-WT or ASFV-Del2R. The data are presented as the mean ± SD from three independent experiments. Statistical significance was analyzed by one-way ANOVA (ns, not significant; $**P < 0.01$; $***P < 0.001$).

Table S1. Amino acid residues of pMGF300-2R from various ASFV strains

Nos.	ASFV strains	Accession numbers	Genotypes	Amino acid sequences
1	HLJ/18	MK333180.1	II	MITLYEAAIKTLITHRKQILKHPDSREILLALGLY WDKTHILVKCRECGNMSLTGKHSTKCININCLL ILAIKKKNKRIVDTLIGMGADVTYIHLLKNKIKLS YNQLSMLKSNSQISLKELHAICYLLYGRLPKKIK QGMRLCKTMAGLCGELLCAFLAP
2	wbBS01	MK645909.1	II	MITLYEAAIKTLITHRKQILKHPDSREILLALGLY WDKTHILVKCRECGKMSLTGKHSTKCININCLL ILAIKKKNKRMVDTLIRMGADVTYIHLLKNKIKLS YNQLSMLKSNSQISLKELHAICYLLYGRLPKKIK QGMQLCKTMAGLCGELLCAFLAP
3	Kashino 04/13	KJ747406. 1	II	MITLYEAAIKTLITHRKQILKHPDSREILLALGLY WDKTHILVKCRECGNMSLTGKHSTKCININCLL ILAIKKKNKRIVDTLIGMGADVTYIHLLKNKIKLS YNQLSMLKSNSQISLKELHAICYLLYGRLPKKIK QGMRLCKTMAGLCGELLCAFLAP
4	Pol16-20 186-o7	MG939583. 1	II	MITLYEAAIKTLITHRKQILKHPDSREILLALGLY WDKTHILVKCRECGNMSLTGKHSTKCININCLL ILAIKKKNKRIVDTLIGMGADVTYIHLLKNKIKLS YNQLSMLKSNSQISLKELHAICYLLYGRLPKKIK QGMRLCKTMAGLCGELLCAFLAP
5	Georgia 2007/1	FR682468.2	II	MITLYEAAIKTLITHRKQILKHPDSREILLALGLY WDKTHILVKCRECGNMSLTGKHSTKCININCLL ILAIKKKNKRIVDTLIGMGADVTYIHLLKNKIKLS YNQLSMLKSNSQISLKELHAICYLLYGRLPKKIK QGMRLCKTMAGLCGELLCAFLAP
6	Belgium 2018/1	LR536725. 1	II	MITLYEAAIKTLITHRKQILKHPDSREILLALGLY WDKTHILVKCRECGNMSLTGKHSTKCININCLL ILAIKKKNKRIVDTLIGMGADVTYIHLLKNKIKLS YNQLSMLKSNSQISLKELHAICYLLYGRLPKKIK QGMRLCKTMAGLCGELLCAFLAP
7	ASFV/LT 14/1490	MK628478. 1	II	MITLYEAAIKTLITHRKQILKHPDSREILLALGLY WDKTHILVKCRECGNMSLTGKHSTKCININCLL ILAIKKKNKRIVDTLIGMGADVTYIHLLKNKIKLS YNQLSMLKSNSQISLKELHAICYLLYGRLPKKIK QGMRLCKTMAGLCGELLCAFLAP
8	Estonia 2014	LS478113. 1	II	MITLYEAAIKTLITHRKQILKHPDSREILLALGLY WDKTHILVKCRECGNMSLTGKHSTKCININCLL ILAIKKKNKRIVDTLIGMGADVTYIHLLKNKIKLS YNQLSMLKSNSQISLKELHAICYLLYGRLPKKIK QGMRLCKTMAGLCGELLCAFLAP

9	CzechRepublic2017/1	LR722600. 1	II	MITLYEAAIKTLITHRKQILKHPDSREILLALGLY<br>WDKTHILVKCRECGNMSLTGKHSTKCININCLL<br>ILAIKKKNKRIVDTLIGMGADVTYIHLLKNKIKLS<br>YNQLSMLKSNSQISLKELHAICYLLYGRLPKKIK<br>QGMRLCKTMAGLCGELLCAFLAP
10	Moldova 2017/1	LR722599. 1	II	MITLYEAAIKTLITHRKQILKHPDSREILLALGLY<br>WDKTHILVKCRECGNMSLTGKHSTKCININCLL<br>ILAIKKKNKRIVDTLIGMGADVTYIHLLKNKIKLS<br>YNQLSMLKSNSQISLKELHAICYLLYGRLPKKIK<br>QGMRLCKTMAGLCGELLCAFLAP
11	SD/DY-I/21	MZ945537	I	MITLYEAAIKTLITHRKQILKHPDSREILLALGLY<br>WDKTHILVKCRECGKMSLTGKHSTKCININCLL<br>ILAIKKKNKRMVDTLIRMGADVTYIHLLKNKIKLS<br>YNQLSMLKSNSQISLKELHAICYLLYGRLPKKIK<br>QGMQLCKTMAGLCGELLCAFLAP
12	HeN/ZZ-P1/21	MZ945536	I	MITLYEAAIKTLITHRKQILKHPDSREILLALGLY<br>WDKTHILVKCRECGKMSLTGKHSTKCININCLL<br>ILAIKKKNKRMVDTLIRMGADVTYIHLLKNKIKLS<br>YNQLSMLKSNSQISLKELHAICYLLYGRLPKKIK<br>QGMQLCKTMAGLCGELLCAFLAP
13	Benin 97/1	AM712239. 1	I	MITLYEAAIKTLITHRKQILKHPDSREILLALGLY<br>WDKTHILVKCRECGKMSLTGKHSTKCININCLL<br>ILAIKKKNKRMVDTLIRMGADVTYIHLLKNKIKLS<br>YNQLSMLKSNSQISLKELHAICYLLYGRLPKKIK<br>QGMQLCKTMAGLCGELLCAFLAP
14	47/Ss/2008	KX354450. 1	I	MITLYEAAIKTLITHRKQILKHPDSREILLALGLY<br>WDKTHILVKCRECGKMSLTGKHSTKCININCLL<br>ILAIKKKNKRMVDTLIRMGADVTYIHLLKNKIKLS<br>YNQLSMLKSNSQISLKELHAICYLLYGRLPKKIK<br>QGMQLCKTMAGLCGELLCAFLAP
15	L60	KM262844. 1	I	MITLYEAAIKTLITHRKQILKHPDSREILLALGLY<br>WDKTHILVKCRECGKMSLTGKHSTKCININCLL<br>ILAIKKKNKRMVDTLIRMGADVTYIHLLKNKIKLS<br>YNQLSMLKSNSQISLKELHAICYLLYGRLPKKIK<br>QGMQLCKTMAGLCGELLCAFLAP
16	NHV	KM262845. 1	I	MITLYEAAIKTLITHRKQILKHPDSREILLALGLY<br>WDKTHILVKCRECGKMSLTGKHSTKCININCLL<br>ILAIKKKNKRMVDTLIRMGADVTYIHLLKNKIKLS<br>YNQLSMLKSNSQISLKELHAICYLLYGRLPKKIK<br>QGMQLCKTMAGLCGELLCAFLAP
17	OURT 88/3	AM712240. 1	I	MITLYEAAIKTLITHRKQILKHPDSREILLALGLY<br>WDKTHILVKCRECGKMSLTGKHSTKCININCLL<br>ILAIKKKNKRMVDTLIRMGADVTYIHLLKNKIKLS<br>YNQLSMLKSNSQISLKELHAICYLLYGRLPKKIK

				QGMQLCKTMAGLCGELLCAFLAP
18	E75	FN557520. 1	I	MITLYEAAIKTLITHRKQILKHPDSREILLALGLY<br>WDKTHILVKCRECGKMSLTGKHSTKCININCLL<br>ILAIKKKNKRMVDTLIRMGADVTYIHLLKNKIKLS<br>YNQLSMLKSNSQISLKELHAICYLLYGRLPKKIK<br>QGMQLCKTMAGLCGELLCAFLAP
19	BA71	KP055815. 1	I	MITLYEAAIKTLITHRKQILKHPDSREILLALGLY<br>WDKTHILVKCRECGKMSLTGKHSTKCININCLL<br>ILAIKKKNKRMVDTLIRMGADVTYIHLLKNKIKLS<br>YNQLSMLKSNSQISLKELHAICYLLYGRLPKKIK<br>QGMQLCKTMAGLCGELLCAFLAP
20	BA71V	U18466. 2	I	MITLYEAAIKTLITHRKQILKHPDSREILLALGLY<br>WDKTHILVKCRECGKMSLTGKHSTKCININCLL<br>ILAIKKKNKRMVDTLIRMGADVTYIHLLKNKIKLS<br>YNQLSMLKSNSQISLKELHAICYLLYGRLPKKIK<br>QGMQLCKTMAGLCGELL
21	HeN/123<br>014/22	OQ504954	I and II<br>recombinat<br>ion	MITLYEAAIKTLITHRKQILKHPDSREILLALGLY<br>WDKTHILVKCRECGKMSLTGKHSTKCININCLL<br>ILAIKKKNKRMVDTLIRMGADVTYIHLLKNKIKLS<br>YNQLSMLKSNSQISLKELHAICYLLYGRLPKKIK<br>QGMQLCKTMAGLCGELLCAFLAP
22	IM/DQD<br>M/22	OQ504955	I and II<br>recombinat<br>ion	MITLYEAAIKTLITHRKQILKHPDSREILLALGLY<br>WDKTHILVKCRECGKMSLTGKHSTKCININCLL<br>ILAIKKKNKRMVDTLIRMGADVTYIHLLKNKIKLS<br>YNQLSMLKSNSQISLKELHAICYLLYGRLPKKIK<br>QGMQLCKTMAGLCGELLCAFLAP
23	JS/LG/21	OQ504956	I and II<br>recombinat<br>ion	MITLYEAAIKTLITHRKQILKHPDSREILLALGLY<br>WDKTHILVKCRECGKMSLTGKHSTKCININCLL<br>ILAIKKKNKRMVDTLIRMGADVTYIHLLKNKIKLS<br>YNQLSMLKSNSQISLKELHAICYLLYGRLPKKIK<br>QGMQLCKTMAGLCGELLCAFLAP
24	Primorsk<br>y 2023<br>DP-4560	PP348677	I and II<br>recombinat<br>ion	MITLYEAAIKTLITHRKQILKHPDSREILLALGLY<br>WDKTHILVKCRECGKMSLTGKHSTKCININCLL<br>ILAIKKKNKRMVDTLIRMGADVTYIHLLKNKIKLS<br>YNQLSMLKSNSQISLKELHAICYLLYGRLPKKIK<br>QGMQLCKTMAGLCGELLCAFLAP
25	VNUA/rA<br>SFV/BG2<br>/2023	PP810979	I and II<br>recombinat<br>ion	MITLYEAAIKTLITHRKQILKHPDSREILLALGLY<br>WDKTHILVKCRECGKMSLTGKHSTKCININCLL<br>ILAIKKKNKRMVDTLIRMGADVTYIHLLKNKIKLS<br>YNQLSMLKSNSQISLKELHAICYLLYGRLPKKIK<br>QGMQLCKTMAGLCGELLCAFLAP

Table S2. The primers, sgRNAs, and siRNAs used in this study

Names	Sequences (5'-3')	Descriptions
GADPH-F	ACATGGCCTCCAAGGAGTAAGA	For quantification of *GADPH*
GADPH-R	GATCGAGTTGGGGCTGTGACT	
FAM134B-F	CTTGCCTTCATCGGGGCCAA	For quantification of *FAM134B*
FAM134B-R	ACGGAAATCAGGTGATACACTCT	
P30-F	ATGAGGGCTCTTGCTCAAAC	For quantification of *p30*
P30-R	AAAAATGATAATGAAACCAATGAATG	
sgRNA1-FAM134B-F	AGAAGGAAGCGCCAGAGGAA	For construction of *FAM134B*-knockout cells
sgRNA1-FAM134B-R	TTCCTCTGGCGCTTCCTTCT	
sgRNA2-FAM134B-F	GCAGCCTGCTTGCCTTCATC	For construction of *FAM134B*-knockout cells
sgRNA2-FAM134B-R	GATGAAGGCAAGCAGGCTGC	
siRNA1-FAM134B-F	GUUAUACUCAGCUAUCUACTT	For silencing *FAM134B*
siRNA1-FAM134B-R	GUAGAUAGCUGAGUAUAACTT	
siRNA2-FAM134B-F	GAUGACAGUGAAUUAGACUTT	For silencing *FAM134B*
siRNA2-FAM134B-R	AGUCUAAUUCACUGUCAUCTT	
Scrambling siRNA-F	UUCUCCGAACGUGUCACGUTT	For RNAi control
Scrambling siRNA-R	ACGUGACACGUUCGGAGAATT	
Flag-MGF300-2R-ΔN-F	GGACGTGACCTACATCCACCTGCTGAAGAACAA	For construct the pFlag-MGF300-2R-ΔN plasmid
Flag-MGF300-2R-ΔN-R	TGGATGTAGGTCACGTCCGCCGCCGCCTTATCGTC	
Flag-MGF300-2R-ΔC-F	ATGGGCGCCTAAGAATTCGGTCGACCGAGATCT	For construct the pFlag-MGF300-2R-ΔC plasmid
Flag-MGF300-2R-ΔC-R	GAATTCTTAGGCGCCCATGCCGATCAGGGTGTC	
Flag-MGF300-2R-ΔLIR1-F	CCGACCTGAAGAACAAGATCAAGCTGAGCTACA	For construct the pFlag-MGF300-2R-ΔLIR1 plasmid
Flag-MGF300-2R-ΔLIR1-R	TCTTGTTCTTCAGGTCGGCGCCCATGCCGATCA	
Flag-MGF300-2R-ΔLIR2-F	CAAGAGCATGCTGAAGAGCAACAGCCAAATCAG	For construct the pFlag-MGF300-2R-ΔLIR2 plasmid
Flag-MGF300-2R-ΔLIR2-R	CTCTTCAGCATGCTCTTGATCTTGTTCTTCAGCAGGTG	
Flag-MGF300-2R-ΔLIR3-F	ATCTGCTACCCCAAGAAGATCAAGCAAGGCATG	For construct the pFlag-MGF300-2R-ΔLIR3 plasmid
Flag-MGF300-2R-ΔLIR3-R	CTTCTTGGGGTAGCAGATGGCGTGCAGCTCCTT	
Flag-MGF300-2R^{Y129A}-F	TCTGCTACCTGCTGGCCGGCAGACTGCCCAAGAAGATC	For construct the pFlag-MGF300-2R^{Y129A} plasmid
Flag-MGF300-2R^{Y129A}-R	GGCCAGCAGGTAGCAGATGGCGTGCAGCTCCT	
Flag-MGF300-2R^{L132A}-F	AGAGCCCCCAAGAAGATCAAGCAAGGCATGAG	For construct the pFlag-MGF300-2R^{L132A} plasmid
Flag-MGF300-2R^{L132A}-R	ATCTTCTTGGGGGCTCTGCCGTACAGCAGGTAGCA	
Flag-MGF300-2R-D2-F	TGGCCGGCAGAGCCCCCAAGAAGATCAAGCAAGGC	For construct the pFlag-MGF300-2R-D2 plasmid
Flag-MGF300-2R-D2-R	TGGGGGCTCTGCCGGCCAGCAGGTAGCAGATGGCGT	
GST-MGF300-2R-ΔLIR3-F	CATGCGATTTGCTATCCGAAAAAAATTAAACAAGGCAT	For construct the pGST-MGF300-2R-ΔLIR3 plasmid
GST-MGF300-2R-ΔLIR3-R	CGGATAGCAAATCGCATGCAGTTCTTTCAGGCT	
GST-MGF300-2R^{Y129A}-F	TTTGCTATCTGCTGGCGGGCCGCCTGCCGAAAAAA	For construct the pGST-MGF300-2R^{Y129A} plasmid
GST-MGF300-2R^{Y129A}-R	CGCCAGCAGATAGCAAATCGCATGCAGTTCTT	
GST-MGF300-2R-D2-F	GCGGGCCGCGCGCCGAAAAAAATTAAACAAGGCAT	For construct the pGST-MGF300-2R-D2 plasmid
GST-MGF300-2R-D2-R	TTTCGGCGCGCGGCCCGCCAGCAGATAGCAAATCGCATGC	
GST-MGF300-2R^{L132A}-F	CGCGCGCCGAAAAAAATTAAACAAGGCATGCG	For construct the pGST-MGF300-2R^{L132A} plasmid
GST-MGF300-2R^{L132A}-R	ATTTTTTTCGGCGCGCGGCCATACAGCAGATAGC	

His-MGF300-2R-ΔLIR3-F	CATGCGATTTGCTATCCGAAAAAAATTAAACAAGGCAT	For construct the pHis-MGF300-2R-ΔLIR3
His-MGF300-2R-ΔLIR3-R	CGGATAGCAAATCGCATGCAGTTCTTTCAGGCT	
GST-LC3B-F	CCCCTGGGATCCCCGGAATTCATGCCGTCCGAGAAAACCTT	For construct the pGST-LC3B
GST-LC3B-R	CTCGAGTCGACCCGGGAATTCTTACACAGACAACTTCATTCCGAAA	
GST-LC3B-K49/51A-F	TGGATGCAACGGCATTCCTGGTGCCTGATCATGTC	For construct the pGST-LC3B-K49/51A
GST-LC3B-K49/51A-R	GAATGCCGTTGCATCCAGGACTGGAAGCTGCTT	
LVX-FAM134B-DelLIR-F	GGATCTATTTCCGGTGAATTCGCCACCATGGCGAGCCCG	For construct the pLVX-FAM134B-DelLIR
LVX-FAM134B-DelLIR-R	AGAACTAGTCTCGAGGAATTCTCACTTATCGTCGTCATCCTTGTAA	
LVX-FAM134B-F	GGATCTATTTCCGGTGAATTCATGTACCCATACGATGTTCCAGATT	For construct the pLVX-FAM134B
LVX-FAM134B-R	AGAACTAGTCTCGAGGAATTCTCACTTGTCGTCATCGTCTTTGTA	
HA-FAM134B-ΔLIR-F	GAAGAGGGCGATCAGAGCGAGCTGGACCAAATC	For construct the pHA-FAM134B-ΔLIR
HA-FAM134B-ΔLIR-R	GCTCTGATCGCCCTCTTCGGTGTCGGTGTCCTC	

Table S3. The protein expression library of ASFV used in this study

Nos.	Proteins	Plasmids
1	pA137R	pFlag-A137R
2	pB169L	pFlag-B169L
3	pB117L	pFlag-B117L
4	pB175L	pFlag-B175L
5	pE184L	pFlag-E184L
6	pCP312R	pFlag-CP312R
7	pD345L	pFlag-D345L
8	pMGF100-1L	pFlag-MGF100-1L
9	pD117L	pFlag-D117L
10	pH240R	pFlag-H240R
11	pE120R	pFlag-E120R
12	pB318L	pFlag-B318L
13	pH339R	pFlag-H339R
14	pNP419L	pFlag-NP419L
15	pD129L	pFlag-D129L
16	pMGF300-1L	pFlag-MGF300-1L
17	pA104R	pFlag-A104R
18	pI10L	pFlag-I10L
19	p14	pFlag-p14
20	p15	pFlag-p15
21	p34	pFlag-p34
22	p35	pFlag-p35
23	p37	pFlag-p37
24	pS273R	pFlag-S273R
25	pEP364R	pFlag-EP364R
26	pD205R	pFlag-D205R
27	pDP238L	pFlag-DP238L
28	pA179L	pFlag-A179L
29	pMGF360-18R	pFlag-MGF360-18R
30	pA151R	pFlag-A151R
31	pE146L	pFlag-E146L
32	pMGF110-1L	pFlag-MGF110-1L
33	pB119L	pFlag-B119L
34	pI243L	pFlag-I243L
35	pCP123L	pFlag-CP123L
36	pMGF505-3R	pFlag-MGF505-3R
37	pMGF505-5R	pFlag-MGF505-5R
38	pMGF360-8L	pFlag-MGF360-8L
39	pMGF360-14L	pFlag-MGF360-14L
40	pE423R	pFlag-E423R
41	pB475L	pFlag-B475L

42	pS183L	pFlag-S183L
43	pE111R	pFlag-E111R
44	pF334L	pFlag-F334L
45	pE301R	pFlag-E301R
46	pC315R	pFlag-C315R
47	pE146L	pFlag-E146L
48	pI226R	pFlag-I226R
49	pI215L	pFlag-I215L
50	p72	pFlag-p72
51	pP1192R	pFlag-P1192R
52	pEP1242L	pFlag-EP1242L
53	pNP868R	pFlag-NP868R
54	pC962R	pFlag-C962R
55	pNP1450L	pFlag-NP1450L
56	pB407L	pFlag-B407L
57	pMGF300-4L	pFlag-MGF300-4L
58	pMGF300-2R	pFlag-MGF300-2R
59	pB354L	pFlag-B354L
60	pA240L	pFlag-A240L
61	pK205R	pFlag-K205R

Figure S9. Unprocessed images of the immunoblotting experiments. Cropped images for figures are indicated by red boxes.

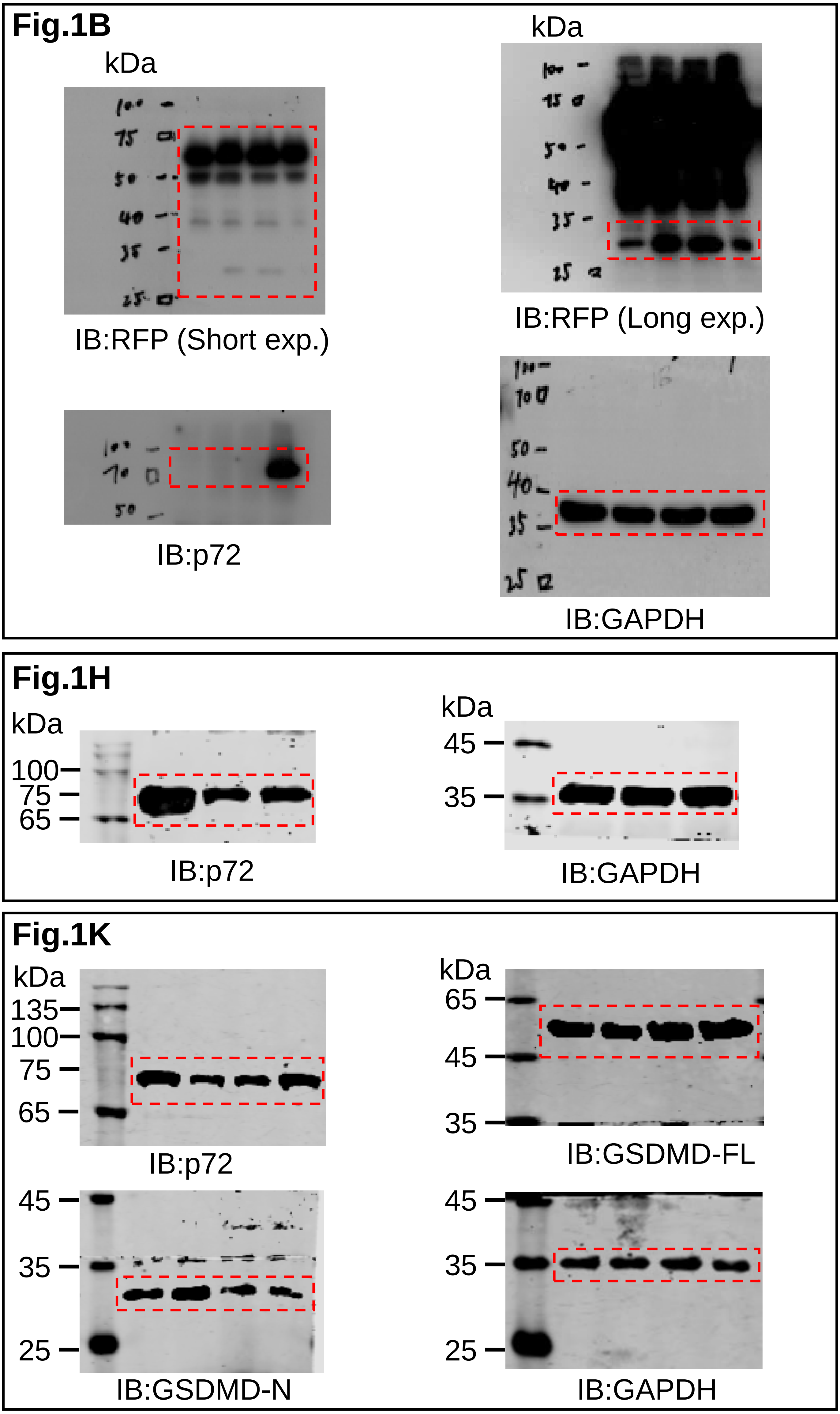

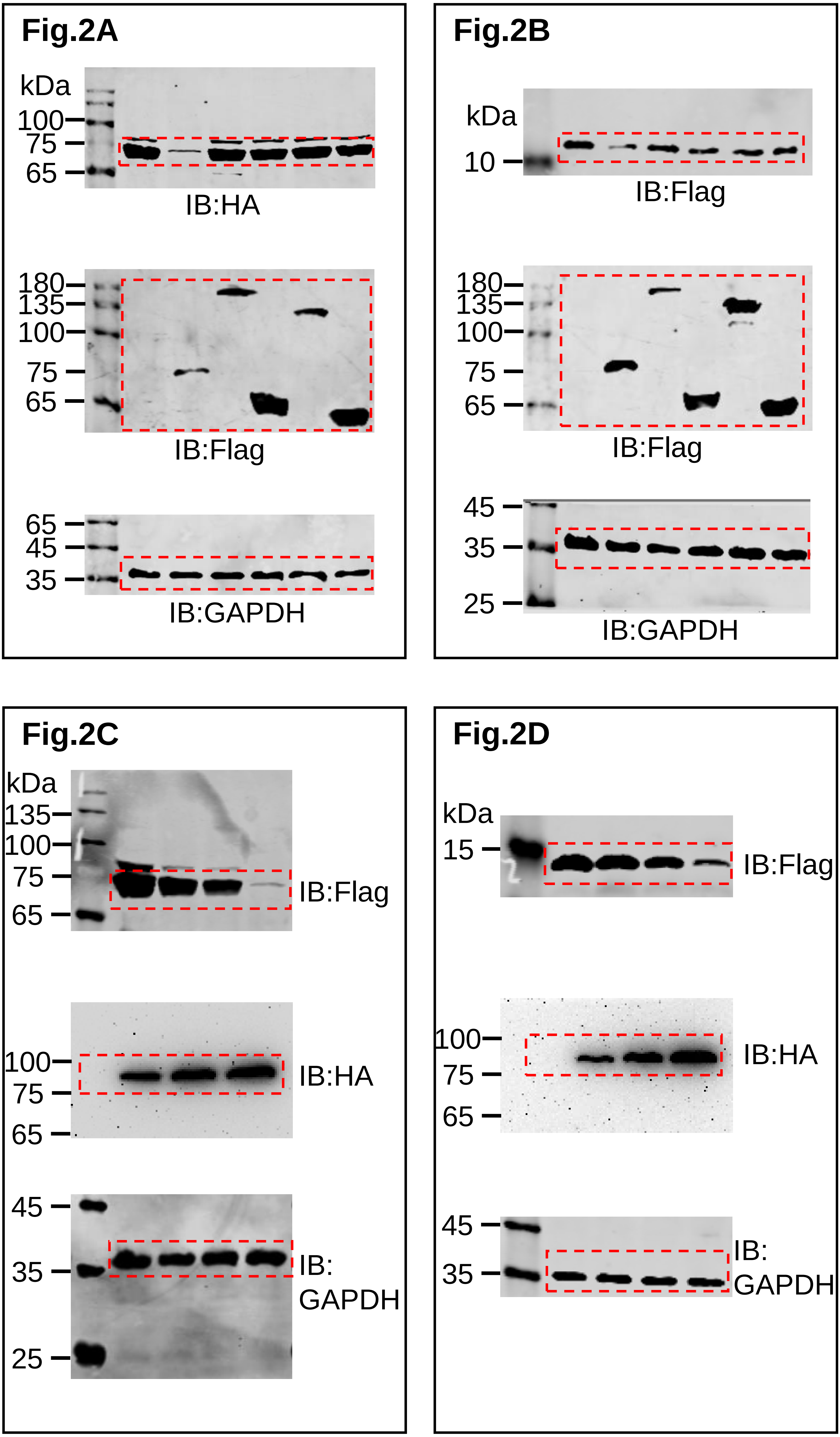

Fig.2A
kDa
100
75
65
IB:HA
180
135
100
75
65
IB:Flag
65
45
35
IB:GAPDH
Fig.2B
kDa
10
IB:Flag
180
135
100
75
65
IB:Flag
45
35
25
IB:GAPDH
Fig.2C
kDa
135
100
75
65
IB:Flag
100
75
65
IB:HA
45
35
25
IB:
GAPDH
Fig.2D
kDa
15
IB:Flag
100
75
65
IB:HA
45
35
IB:
GAPDH

Fig.2E

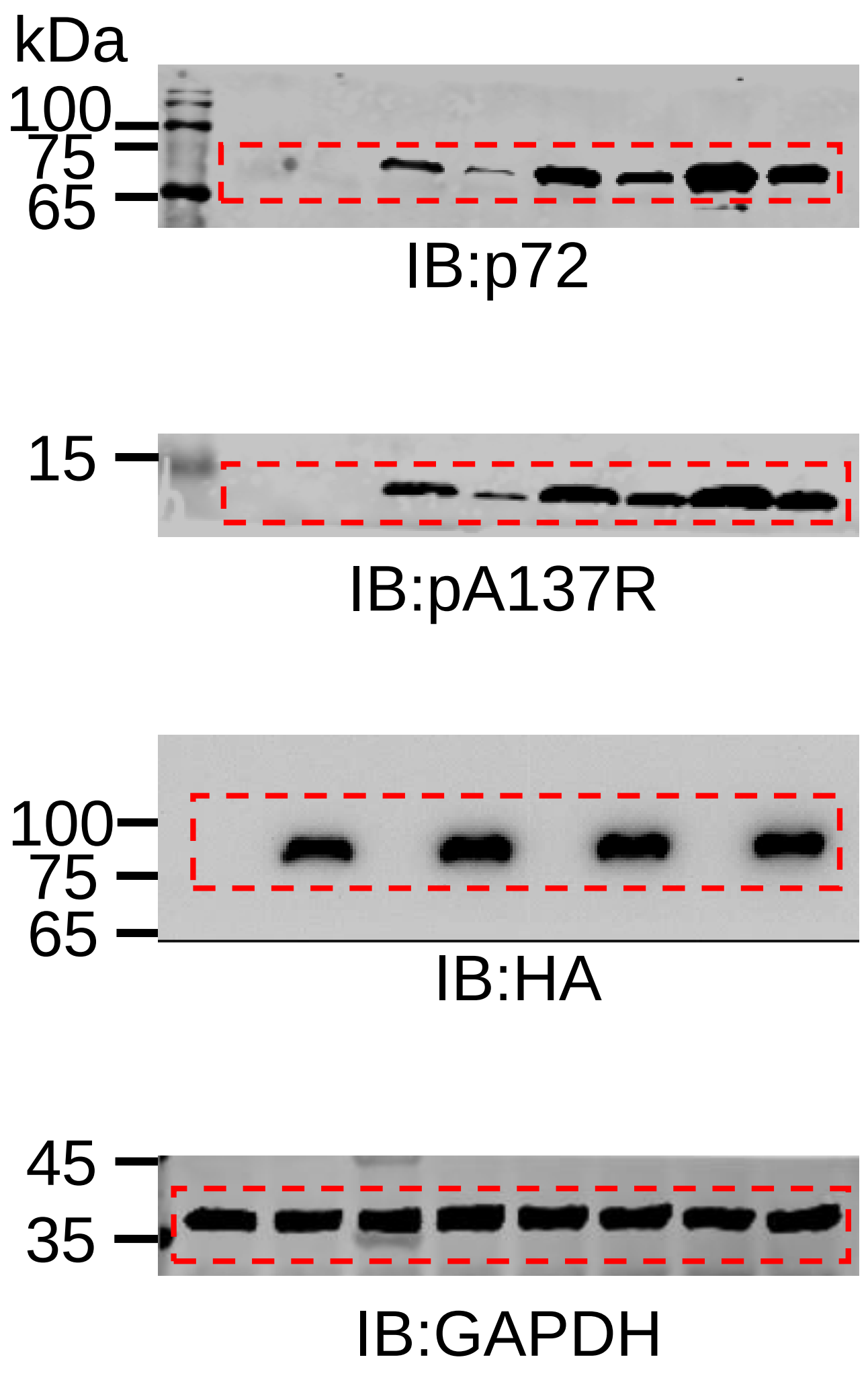

Fig.2G

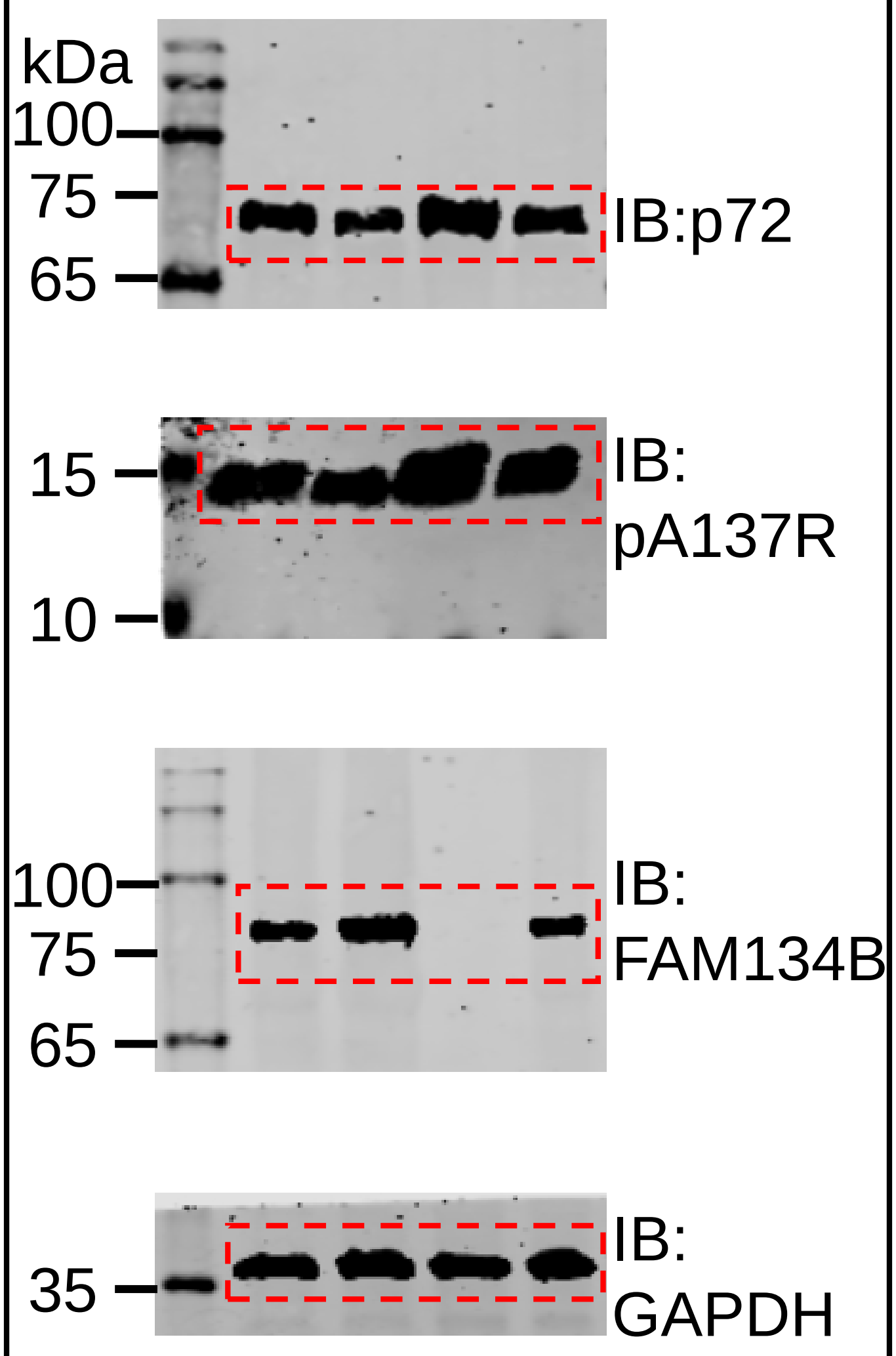

Fig.2J

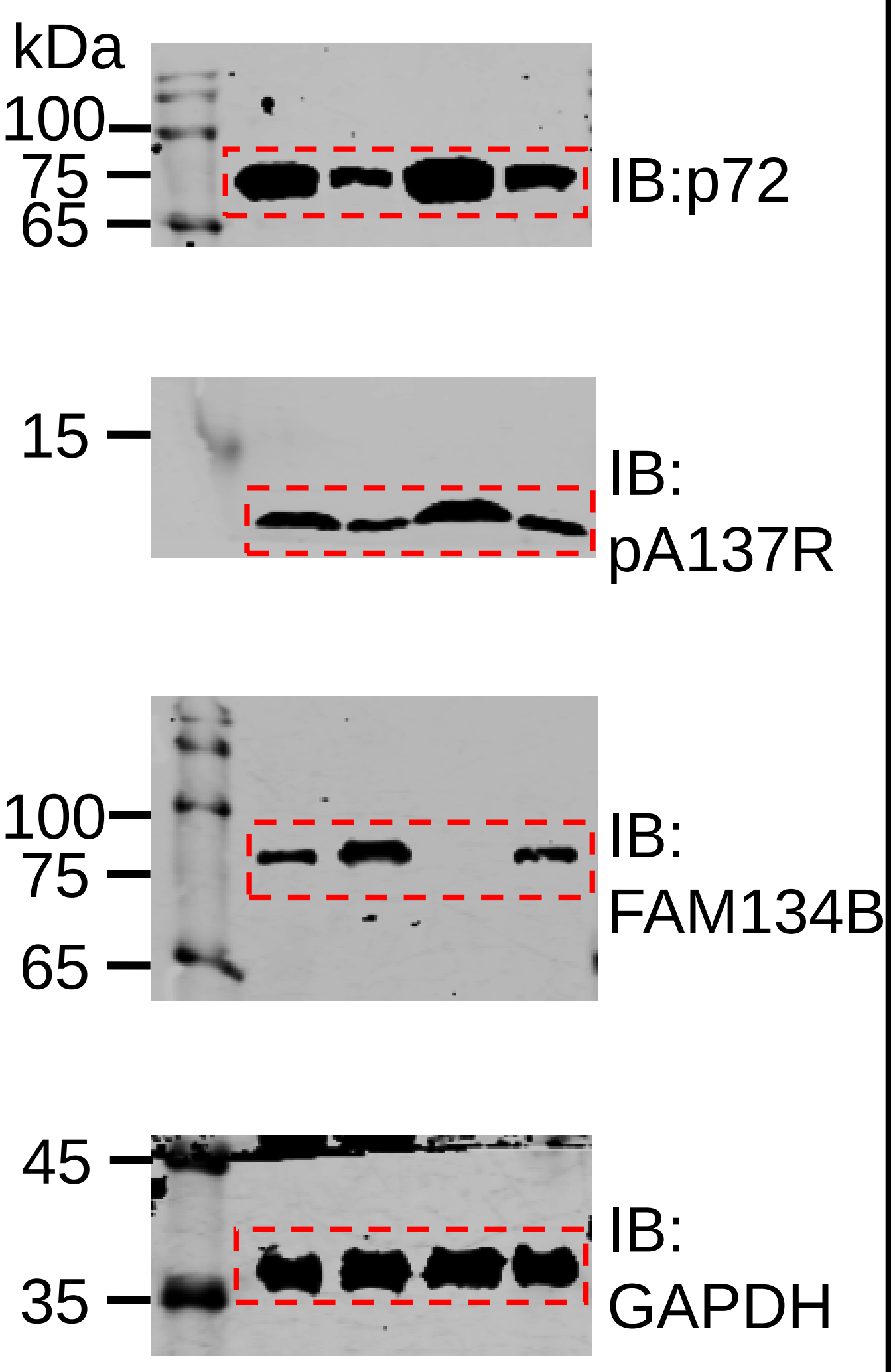

Fig.2M

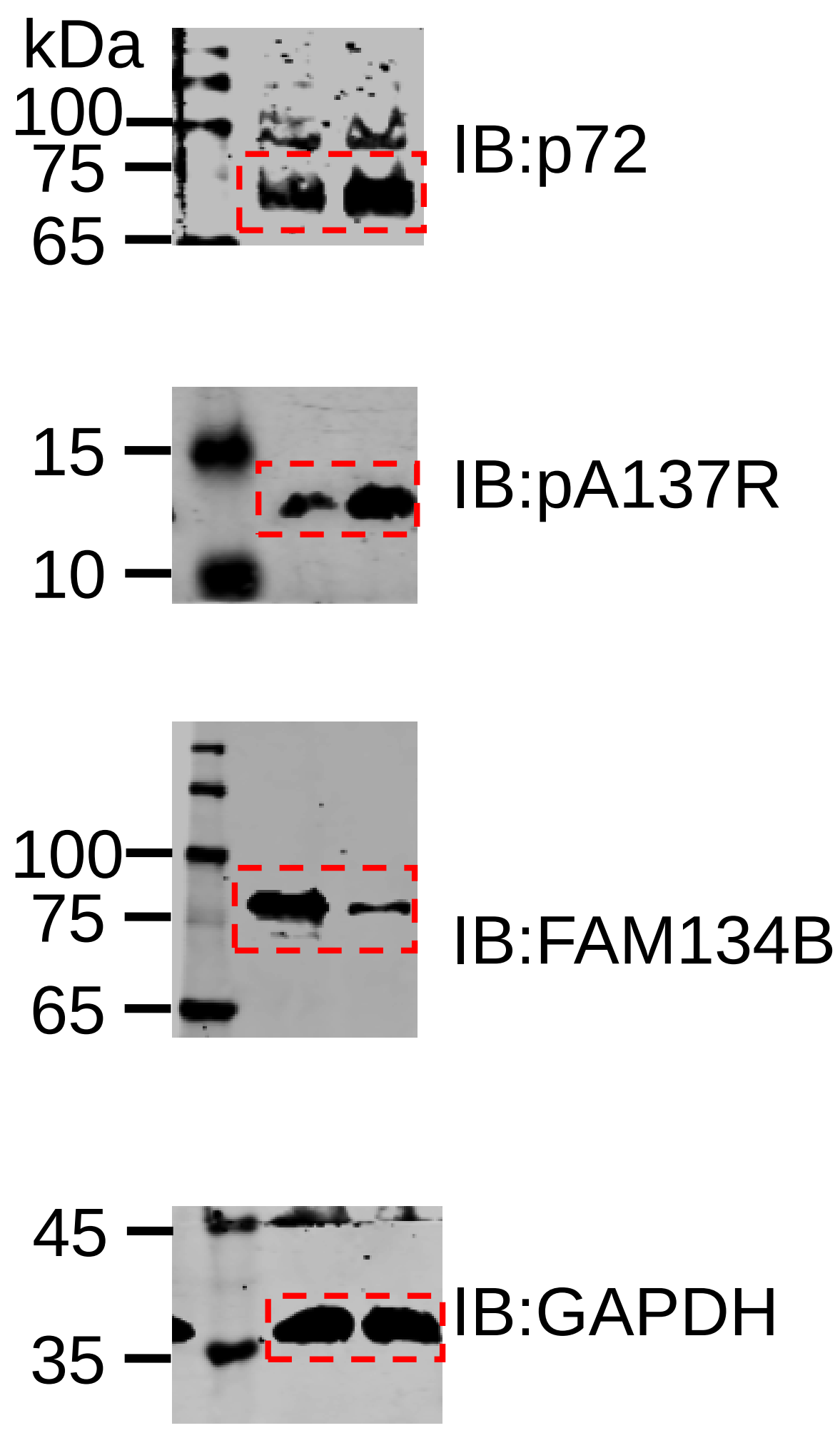

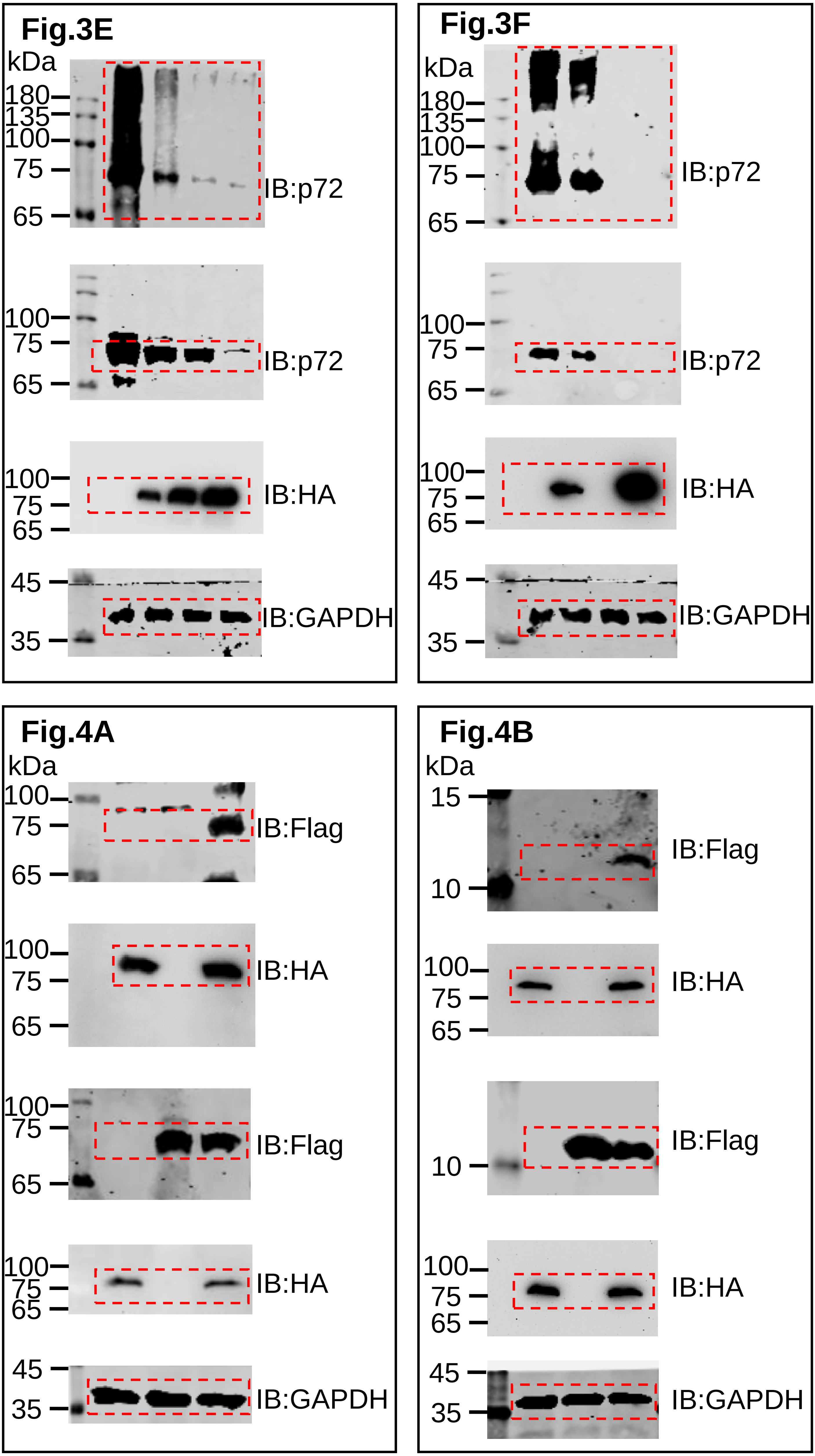

Fig.3E
kDa
180
135
100
75
65
IB:p72
100
75
65
IB:p72
100
75
65
IB:HA
45
35
IB:GAPDH
Fig.3F
kDa
180
135
100
75
65
IB:p72
100
75
65
IB:p72
100
75
65
IB:HA
45
35
IB:GAPDH
Fig.4A
kDa
100
75
65
IB:Flag
100
75
65
IB:HA
100
75
65
IB:Flag
100
75
65
IB:HA
45
35
IB:GAPDH
Fig.4B
kDa
15
10
IB:Flag
100
75
65
IB:HA
10
IB:Flag
100
75
65
IB:HA
45
35
IB:GAPDH

Fig.4C

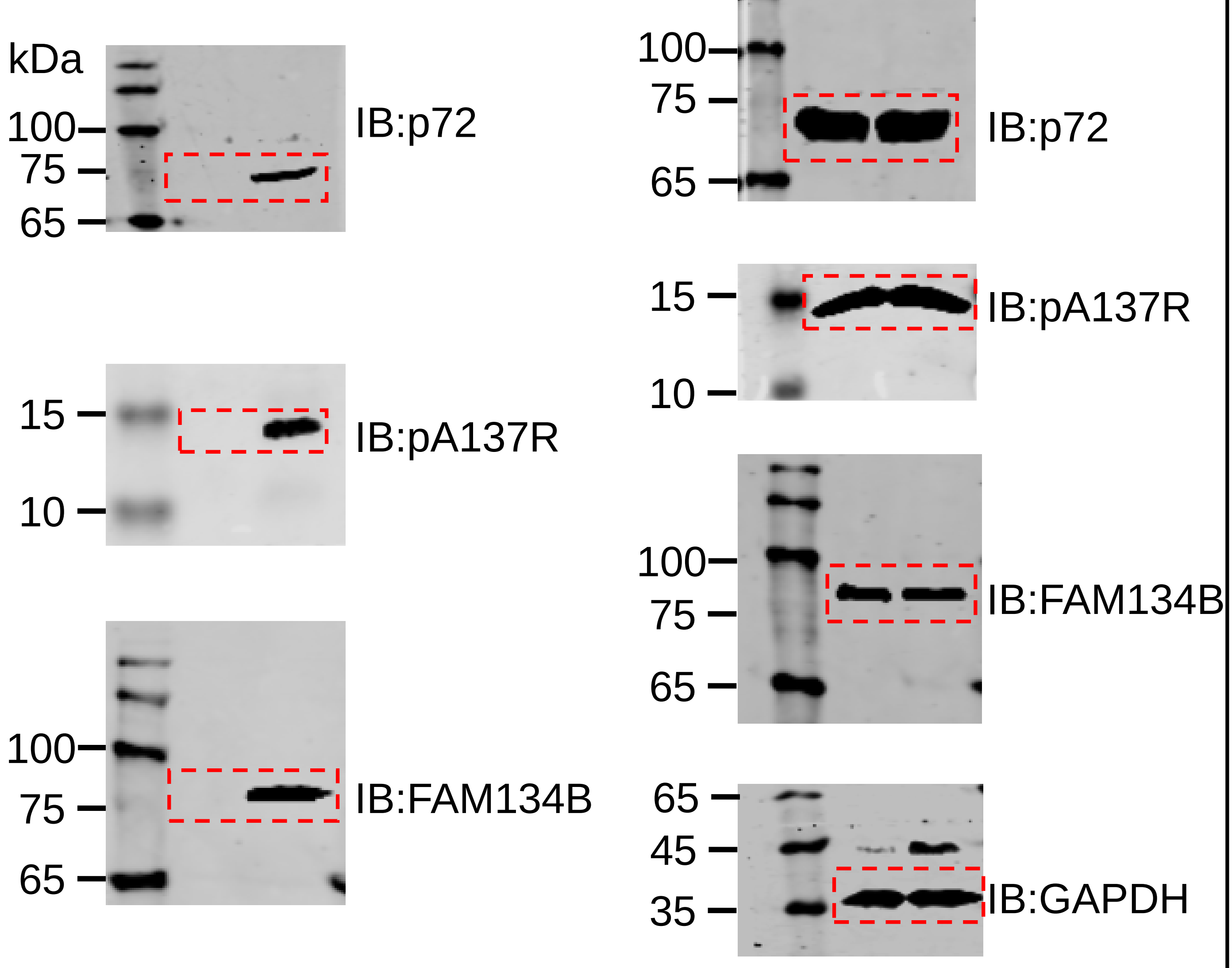

Fig.4D

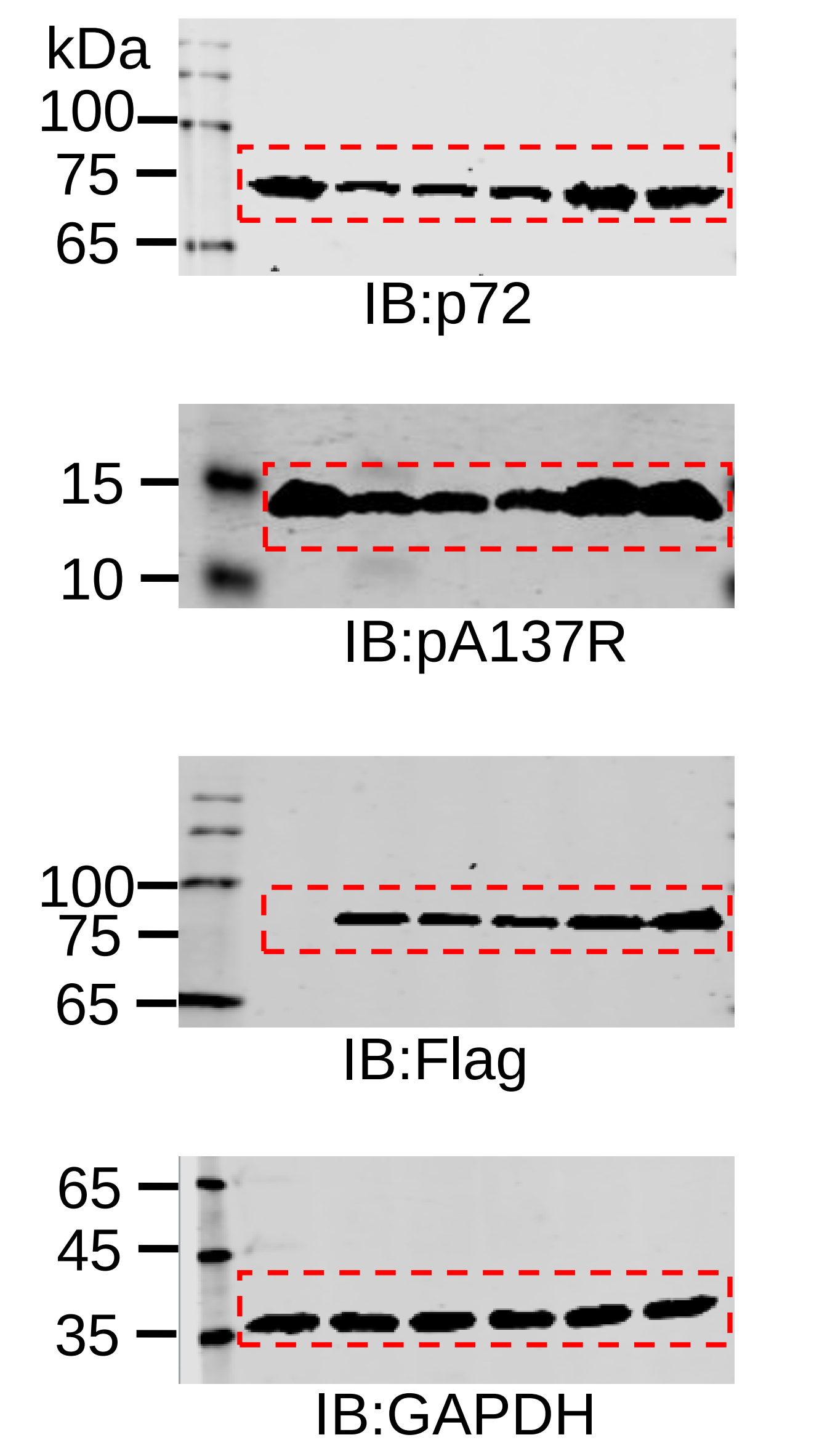

Fig.4F

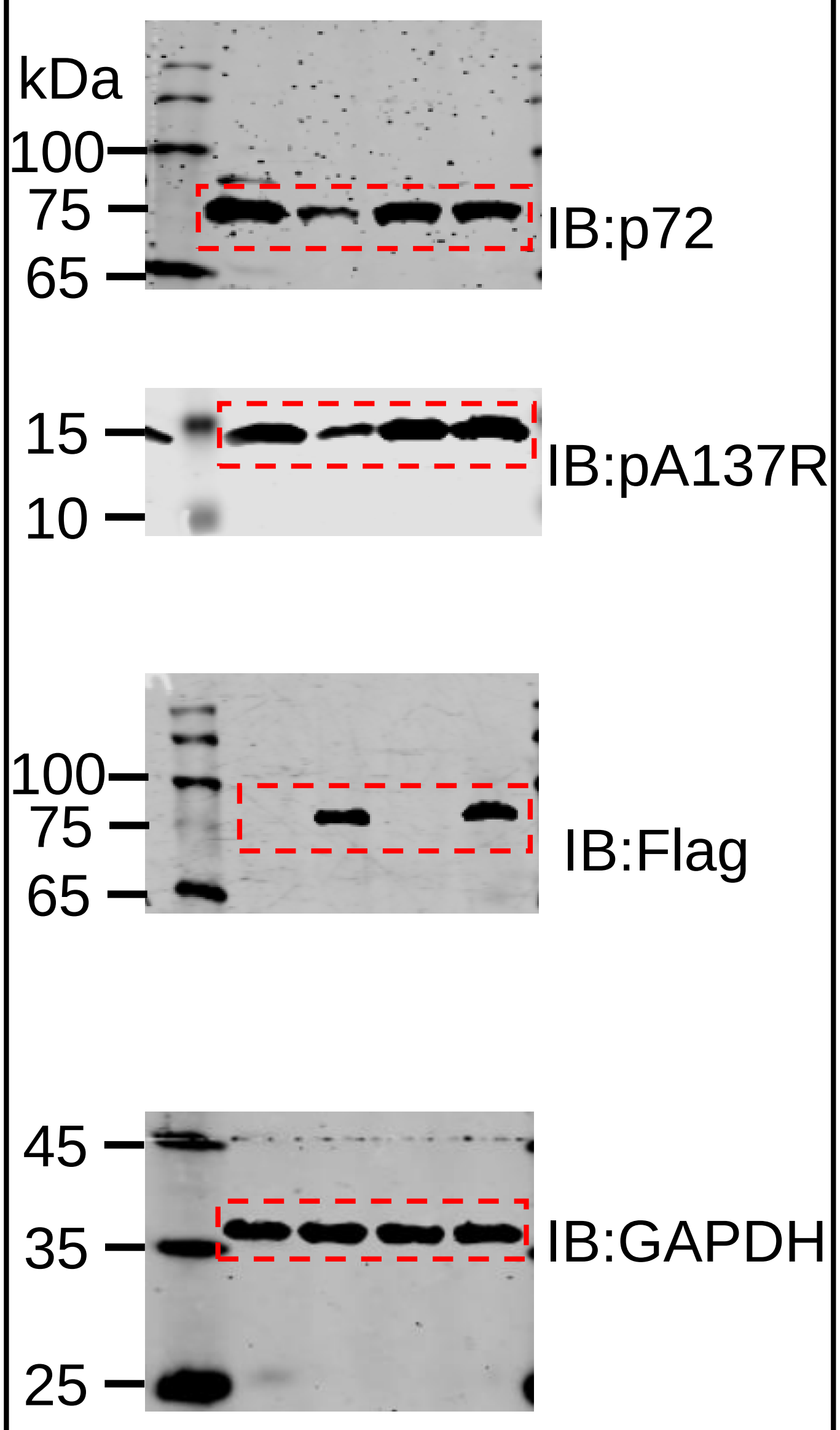

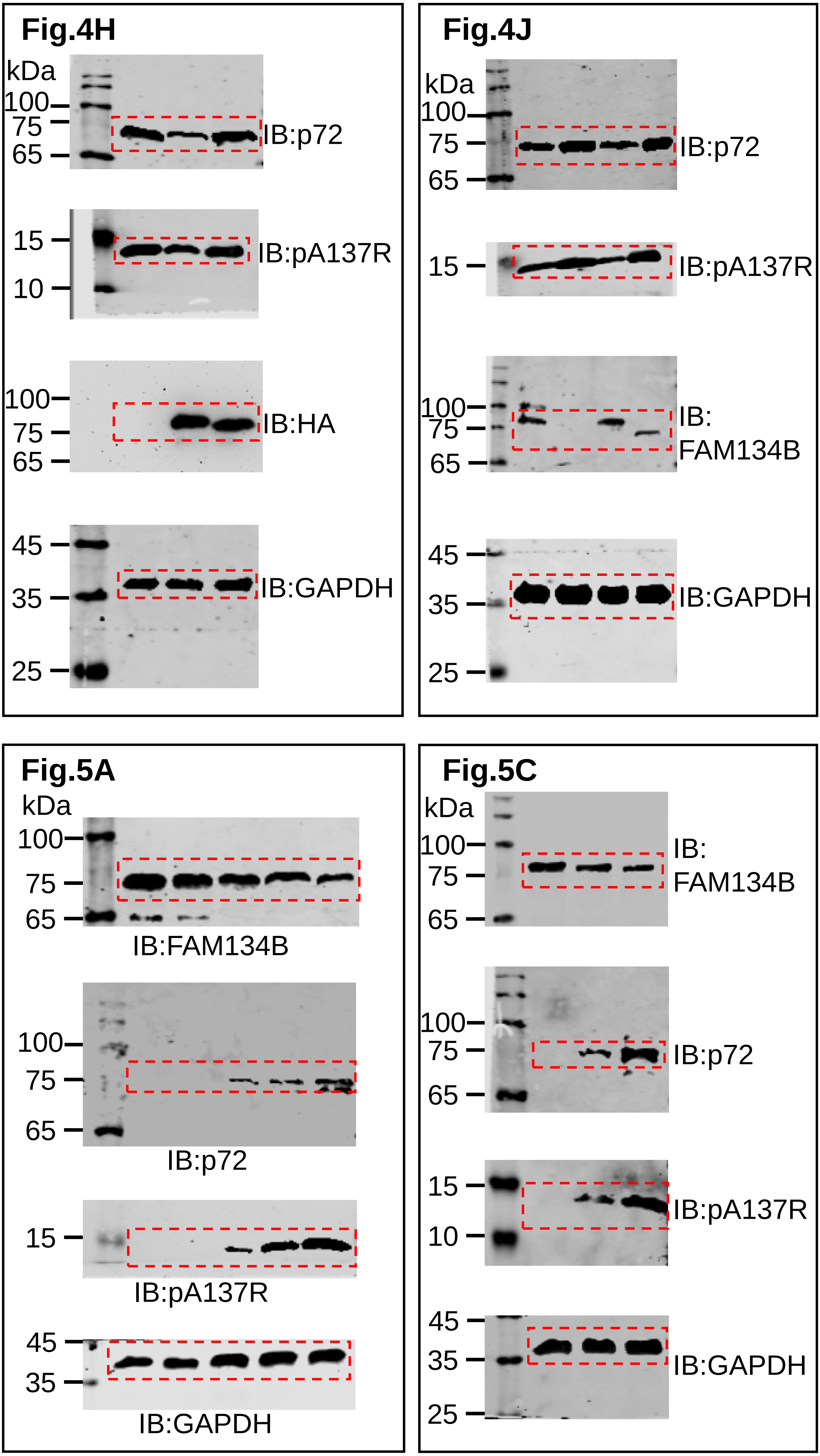

Fig.4H
kDa
100
75
65
IB:p72
15
10
IB:pA137R
100
75
65
IB:HA
45
35
25
IB:GAPDH
Fig.4J
kDa
100
75
65
IB:p72
15
IB:pA137R
100
75
65
IB:
FAM134B
45
35
25
IB:GAPDH
Fig.5A
kDa
100
75
65
IB:FAM134B
100
75
65
IB:p72
15
IB:pA137R
45
35
IB:GAPDH
Fig.5C
kDa
100
75
65
IB:
FAM134B
100
75
65
IB:p72
15
10
IB:pA137R
45
35
25
IB:GAPDH

Fig.5F

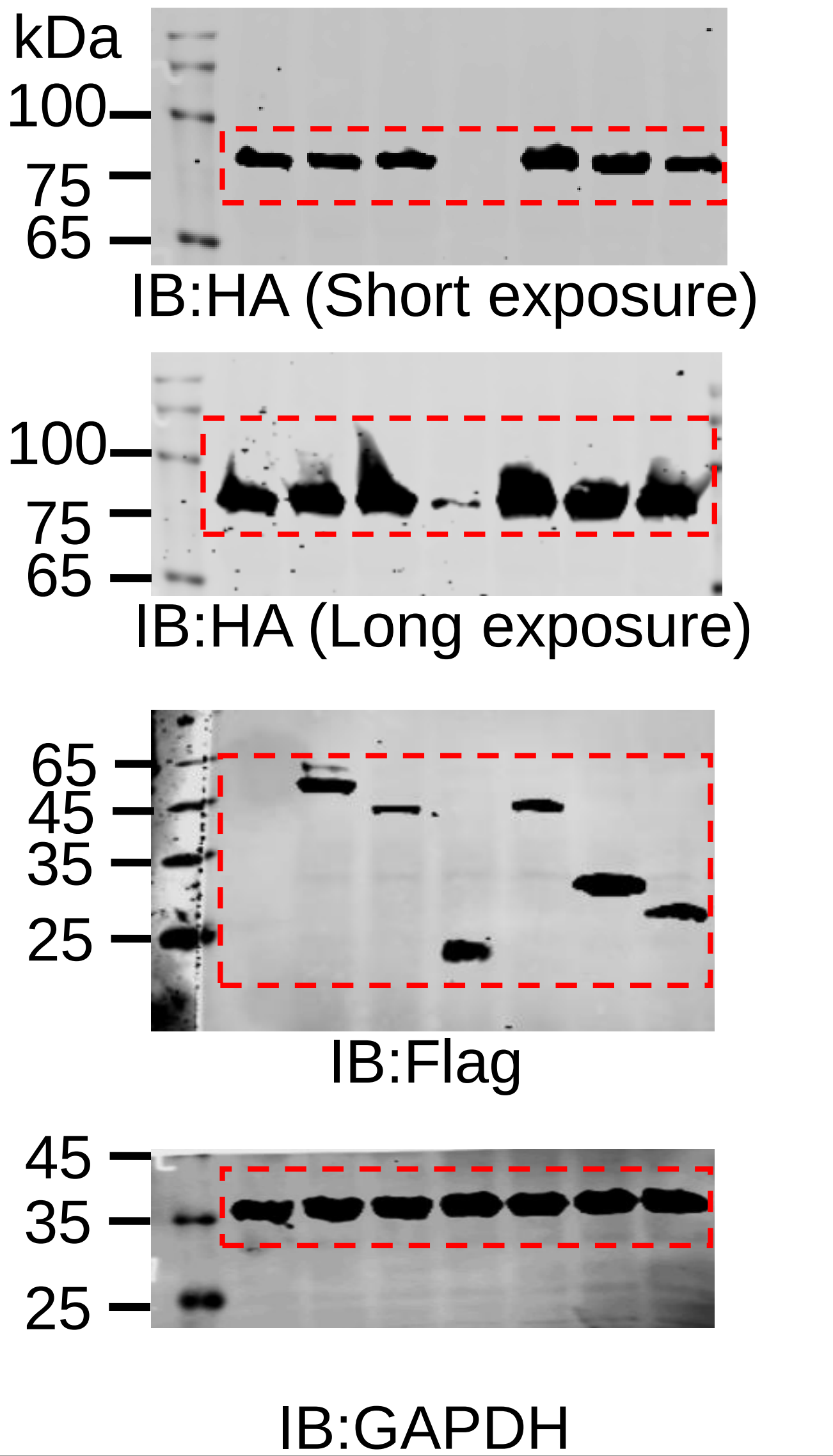

Fig.5G

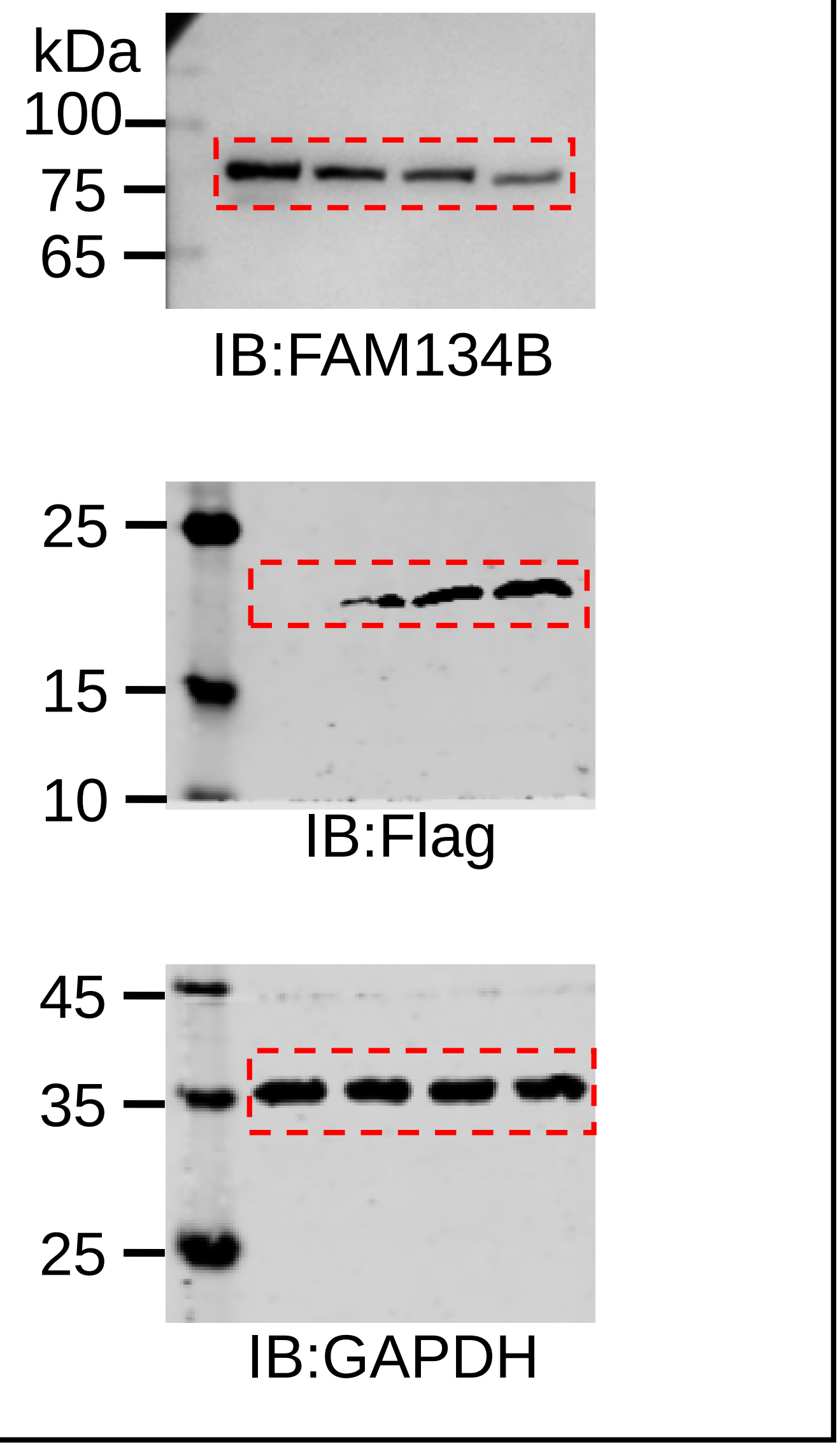

Fig.5I

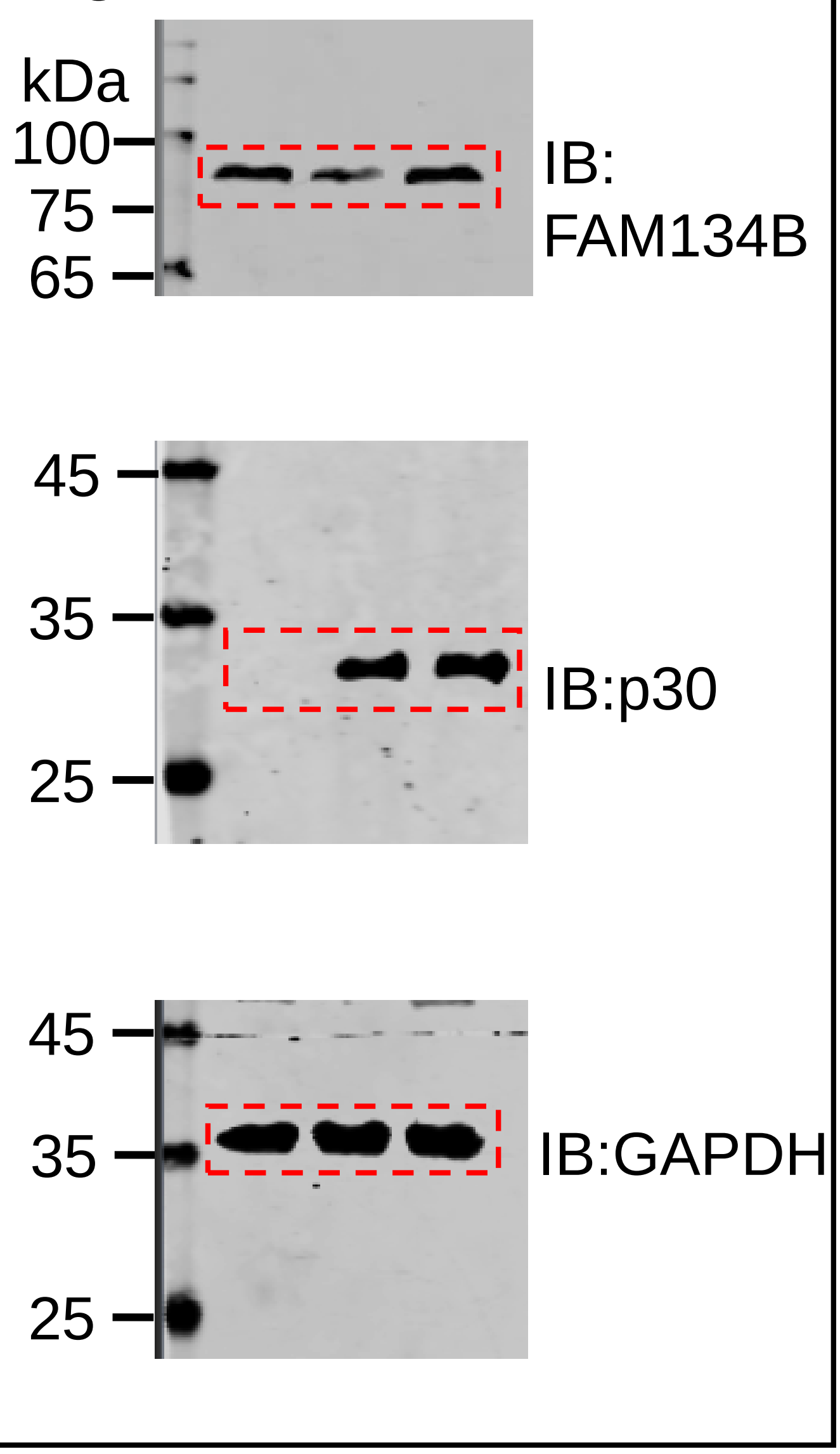

Fig.6A

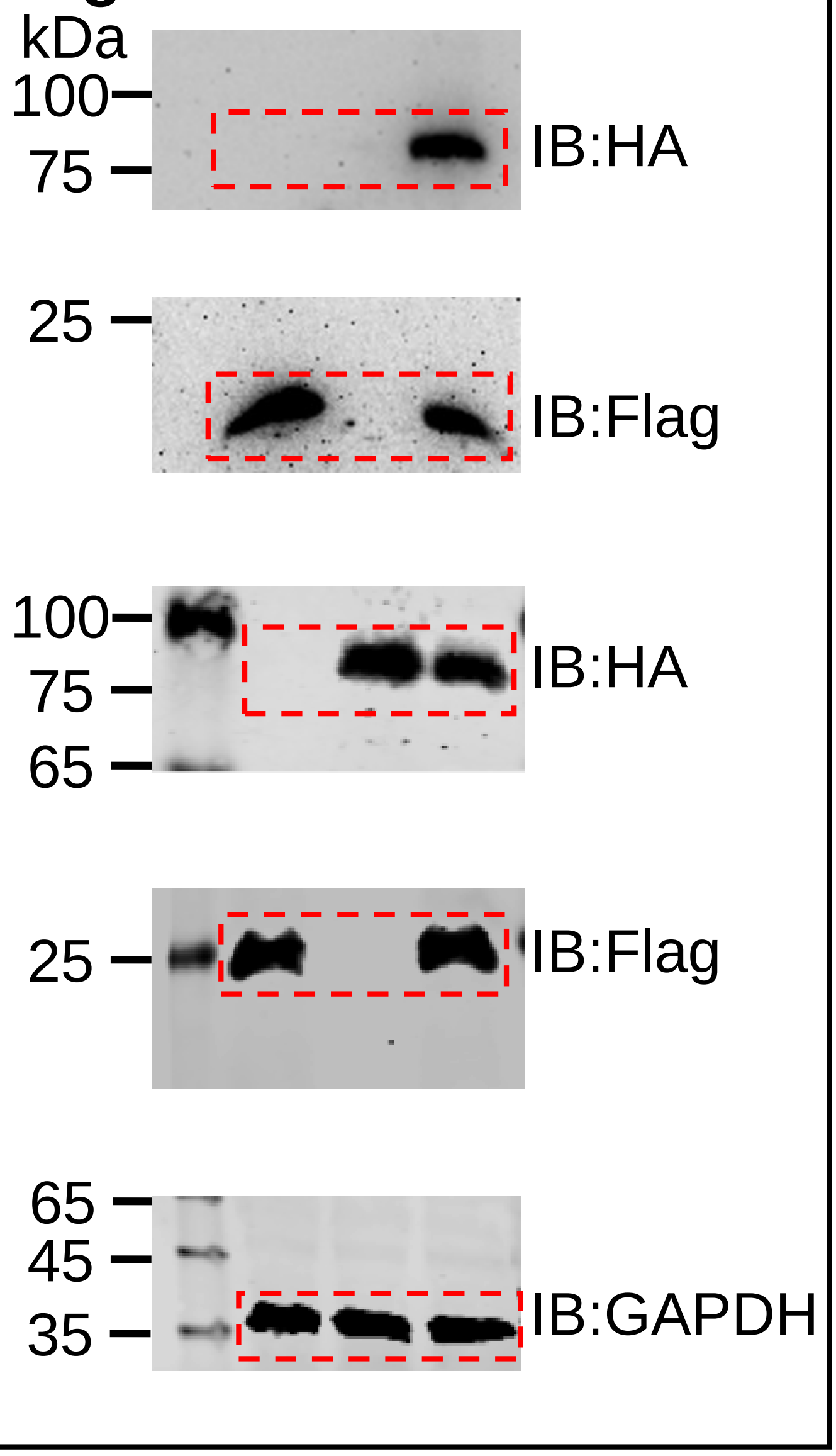

Fig.6B

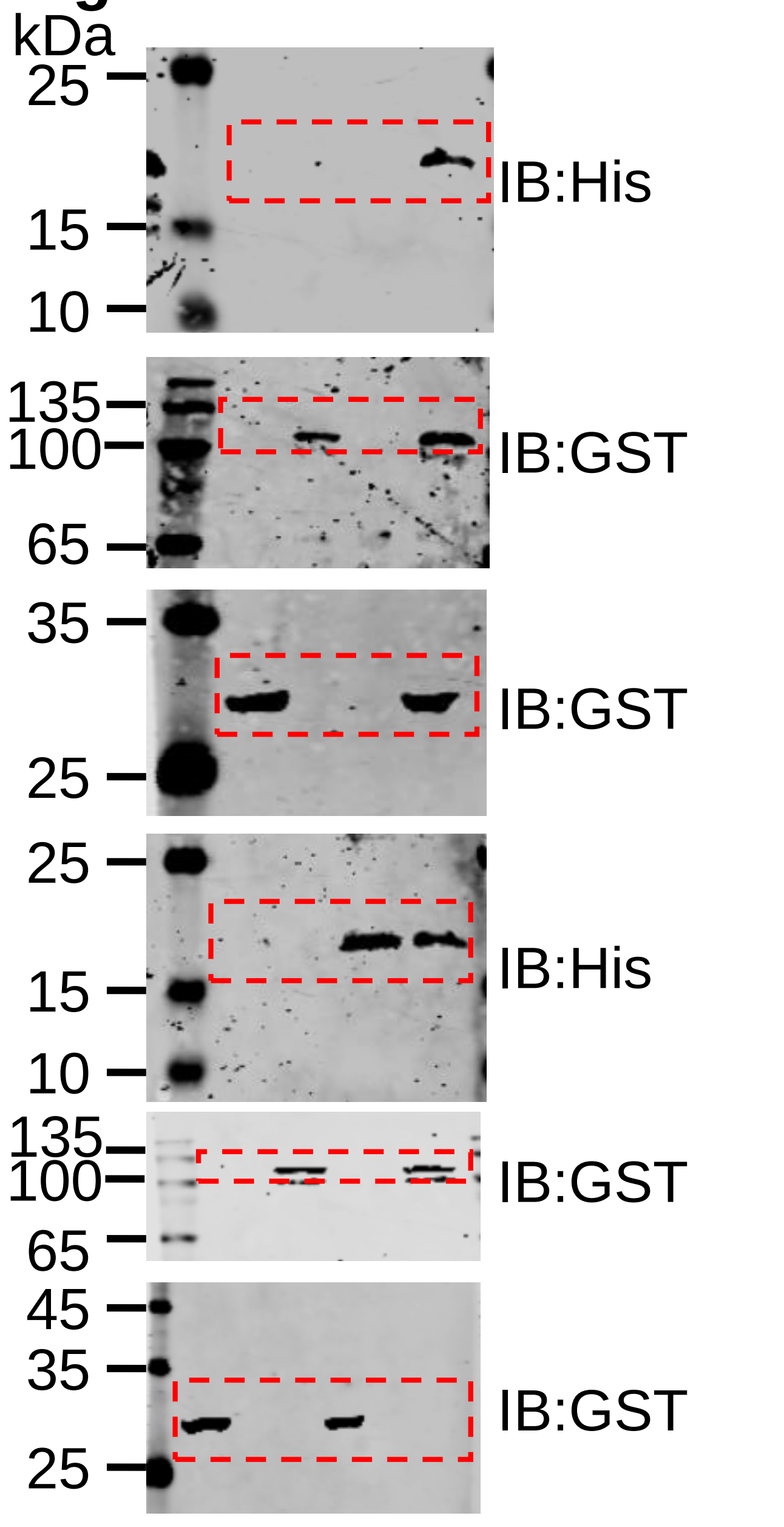

Fig.6E

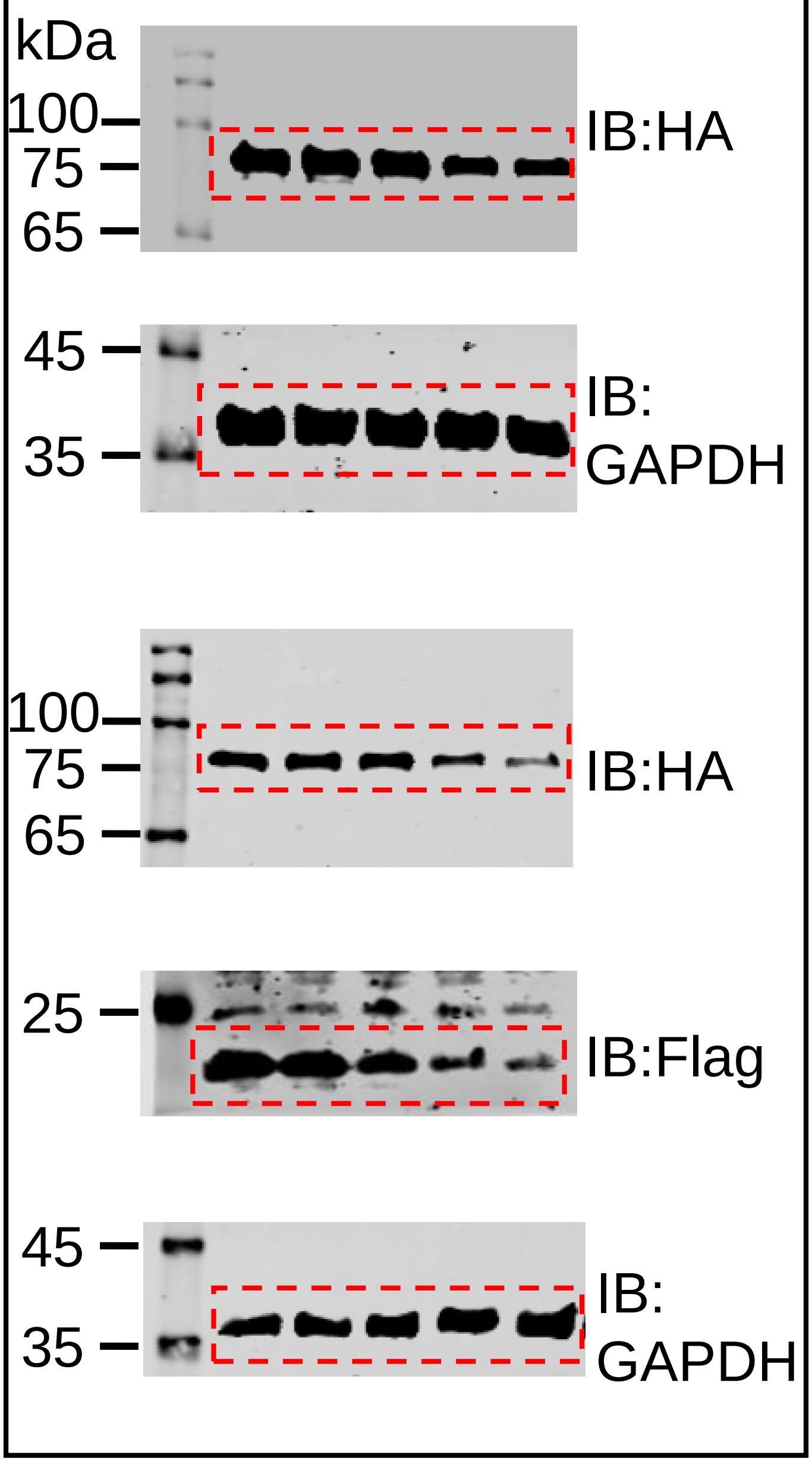

Fig.6F

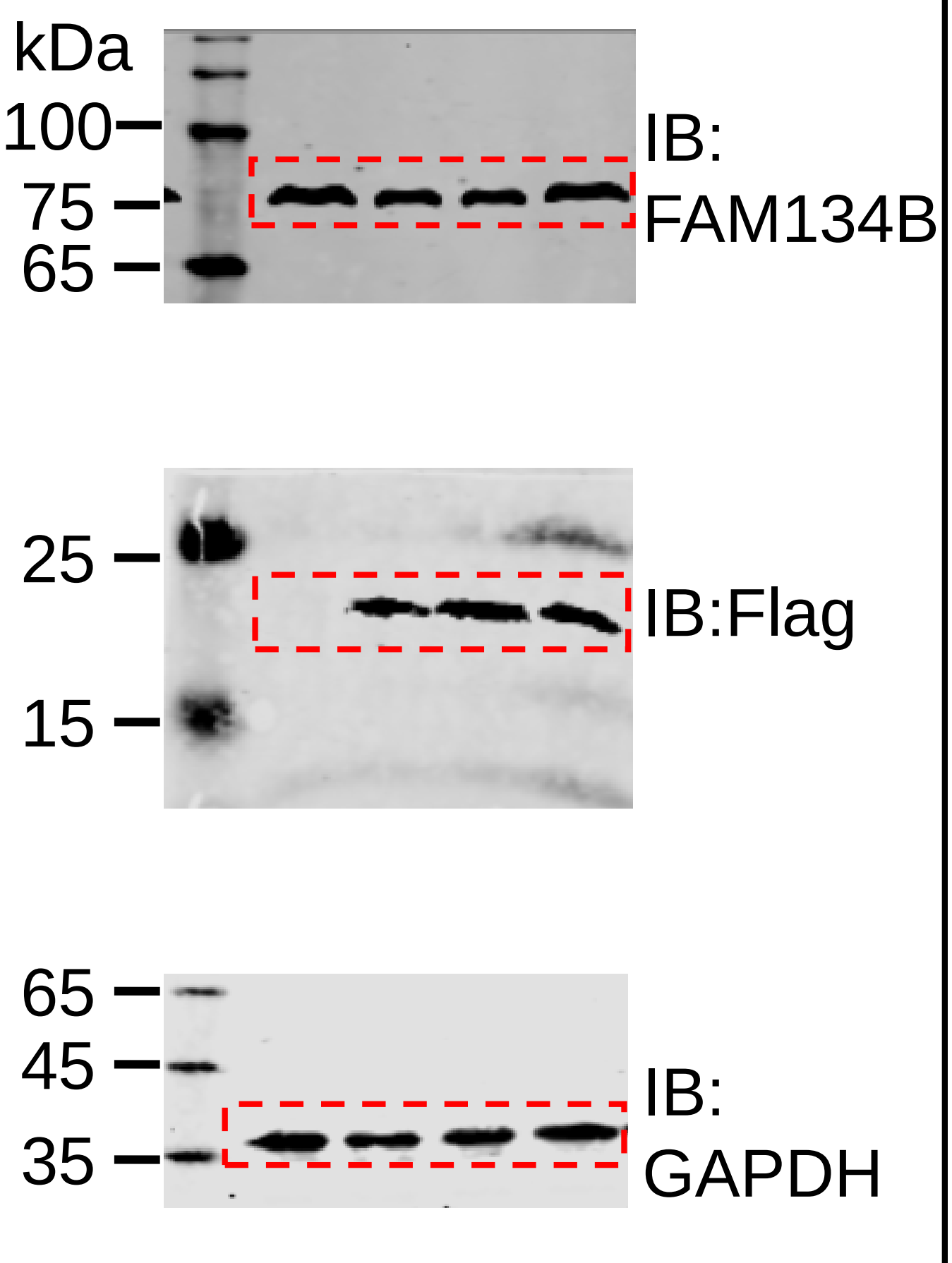

Fig.6H

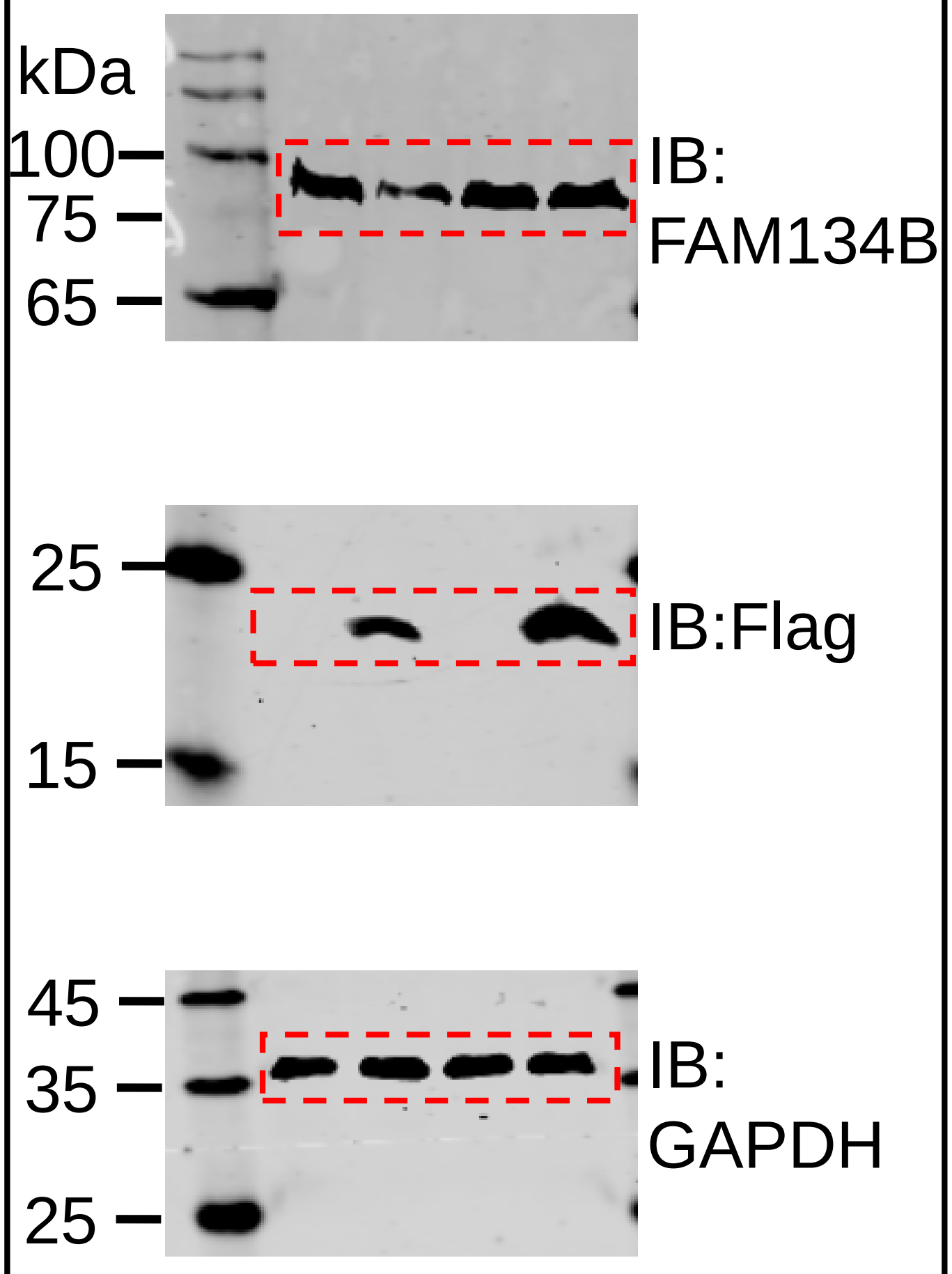

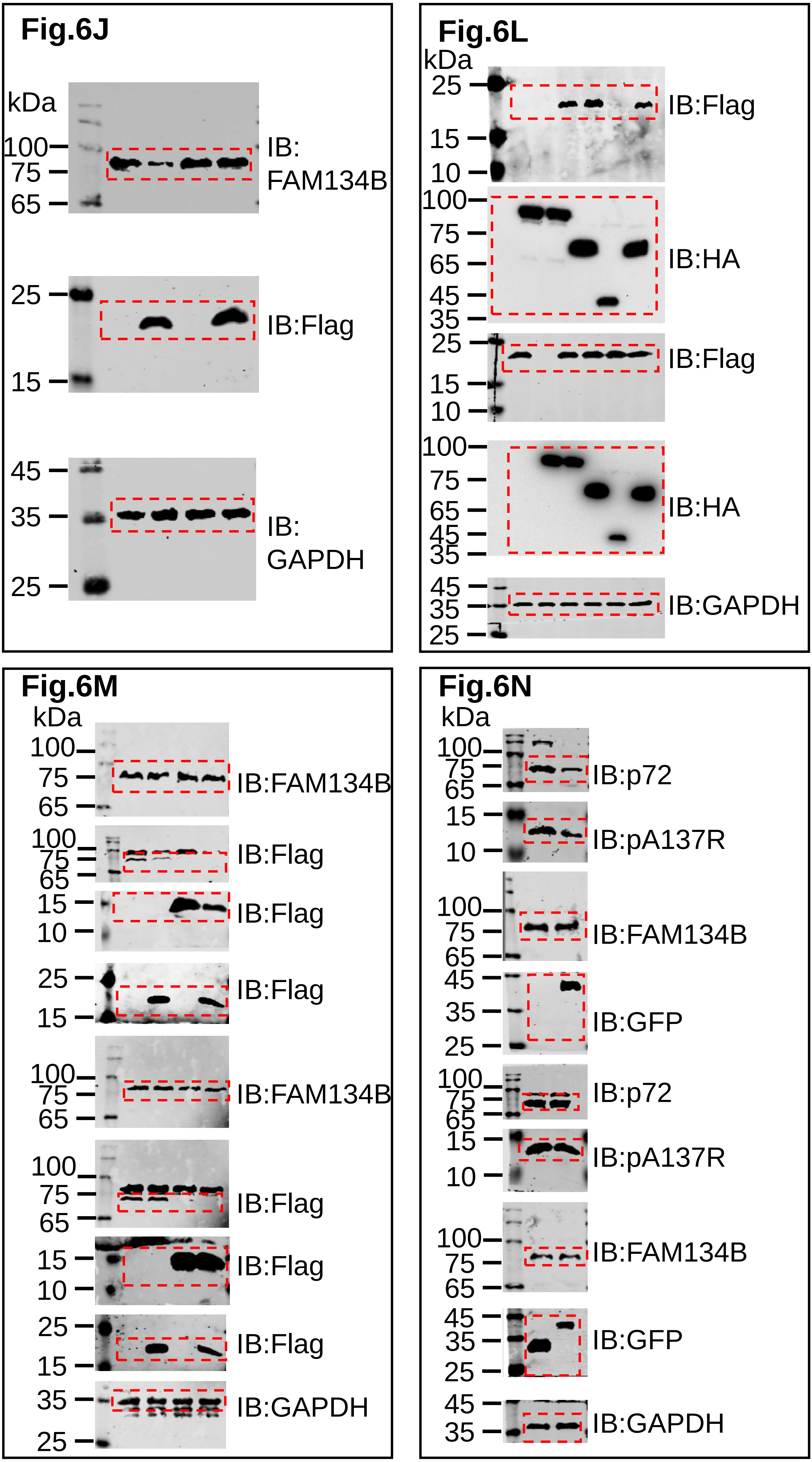

Fig.6J
kDa
100
75
65
IB: FAM134B
25
15
IB:Flag
45
35
25
IB: GAPDH
Fig.6L
kDa
25
15
10
IB:Flag
100
75
65
45
35
IB:HA
25
15
10
IB:Flag
100
75
65
45
35
IB:HA
45
35
25
IB:GAPDH
Fig.6M
kDa
100
75
65
IB:FAM134B
100
75
65
IB:Flag
15
10
IB:Flag
25
15
IB:Flag
100
75
65
IB:FAM134B
100
75
65
IB:Flag
15
10
IB:Flag
25
15
IB:Flag
35
25
IB:GAPDH
Fig.6N
kDa
100
75
65
IB:p72
15
10
IB:pA137R
100
75
65
IB:FAM134B
45
35
25
IB:GFP
100
75
65
IB:p72
15
10
IB:pA137R
100
75
65
IB:FAM134B
45
35
25
IB:GFP
45
35
IB:GAPDH

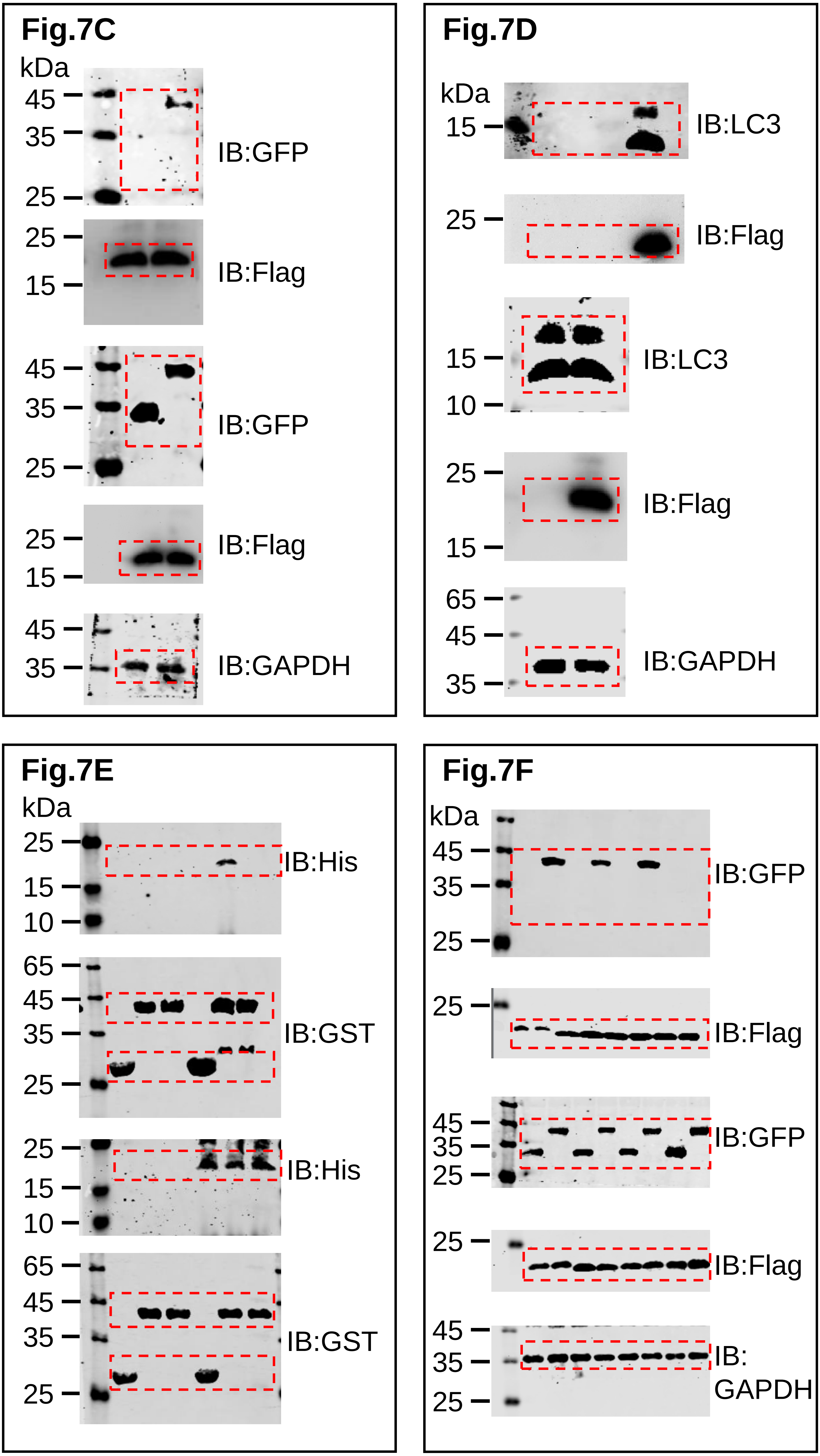
Fig.7C
kDa
45
35
25
IB:GFP
25
15
IB:Flag
45
35
25
IB:GFP
25
15
IB:Flag
45
35
IB:GAPDH
Fig.7D
kDa
15
IB:LC3
25
IB:Flag
15
10
IB:LC3
25
15
IB:Flag
65
45
35
IB:GAPDH
Fig.7E
kDa
25
15
10
IB:His
65
45
35
25
IB:GST
25
15
10
IB:His
65
45
35
25
IB:GST
Fig.7F
kDa
45
35
25
IB:GFP
25
IB:Flag
45
35
25
IB:GFP
25
IB:Flag
45
35
25
IB:
GAPDH

Fig.7G

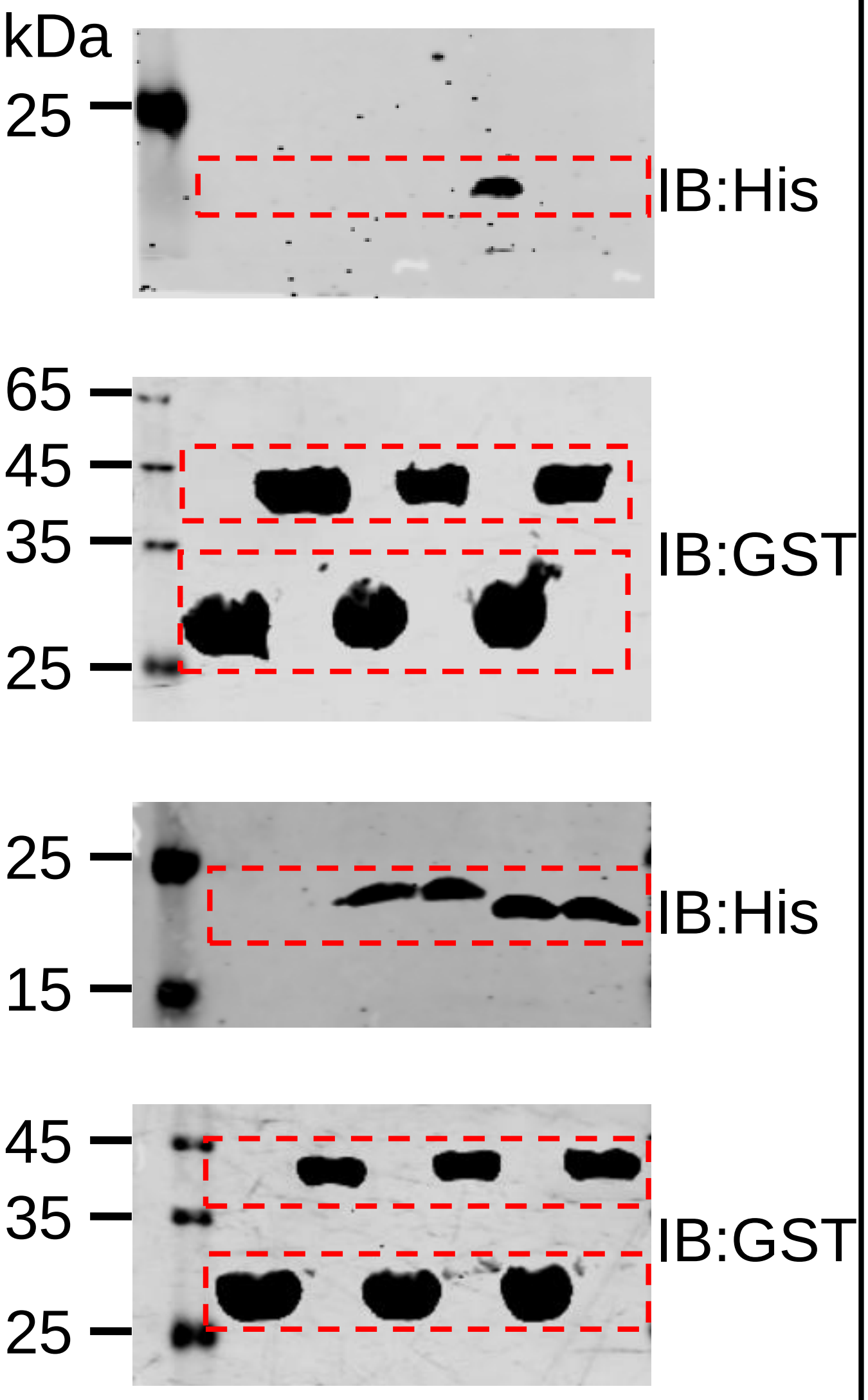

Fig.7H

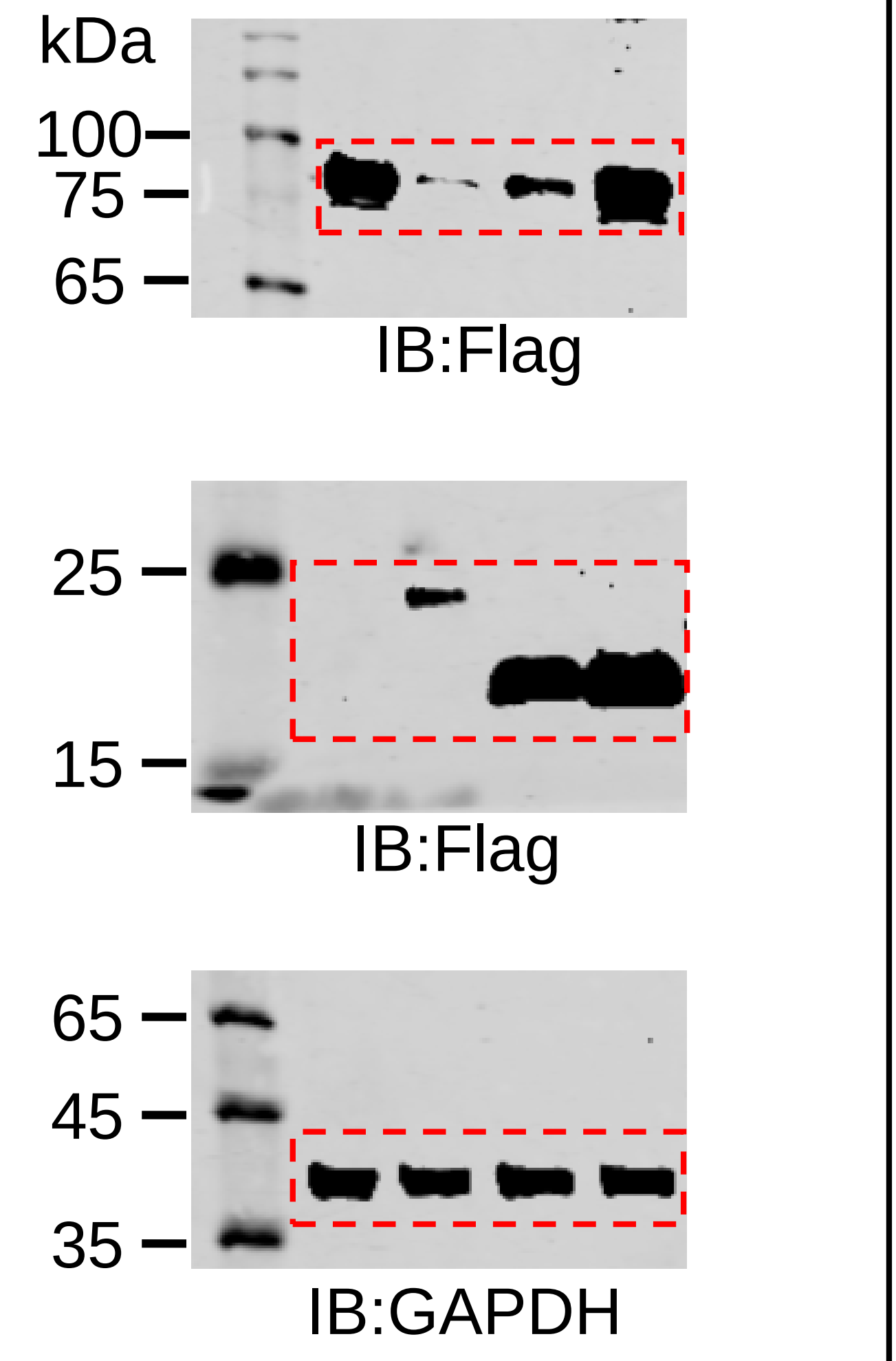

Fig.7I

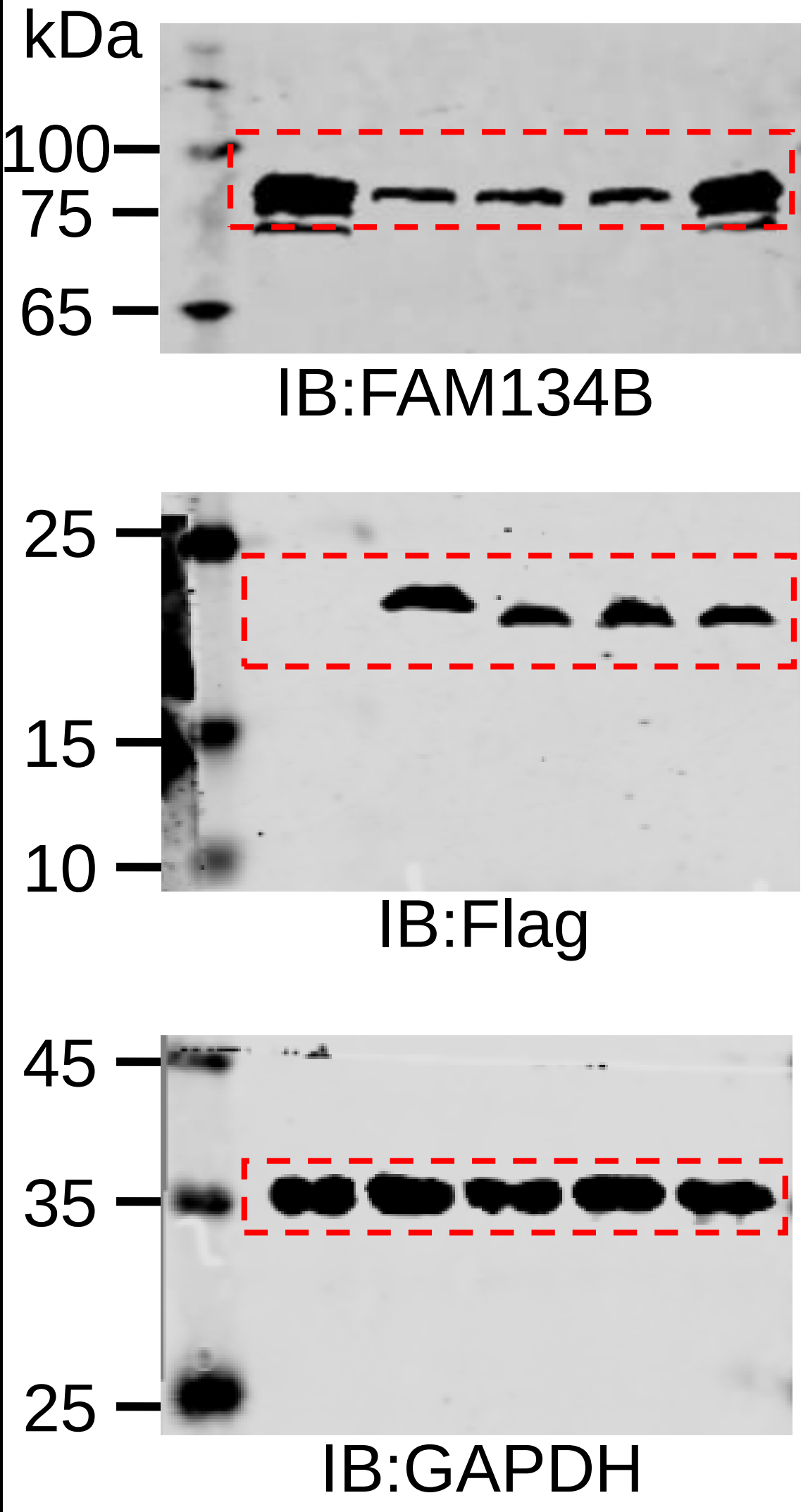

Fig.7L

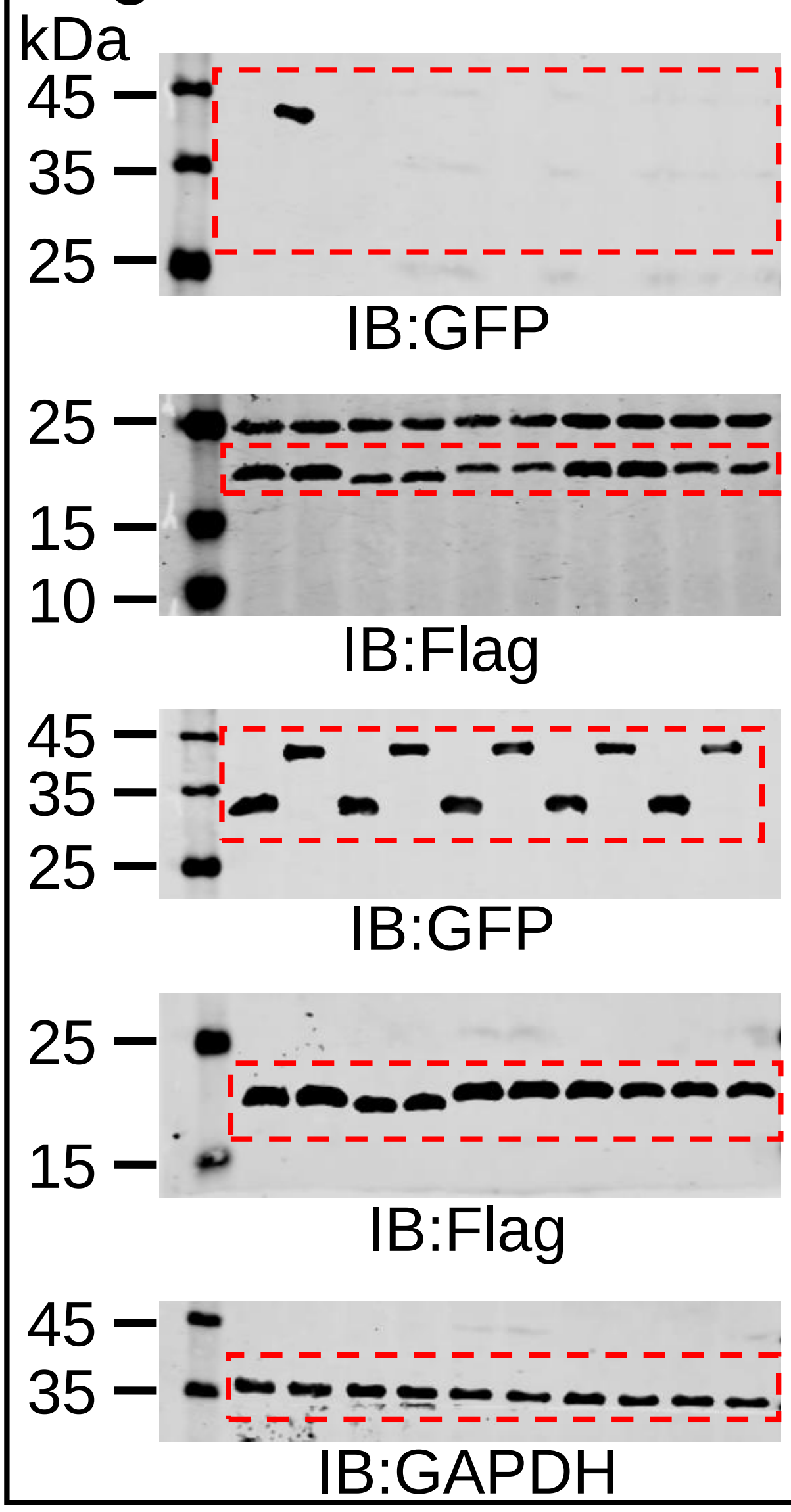

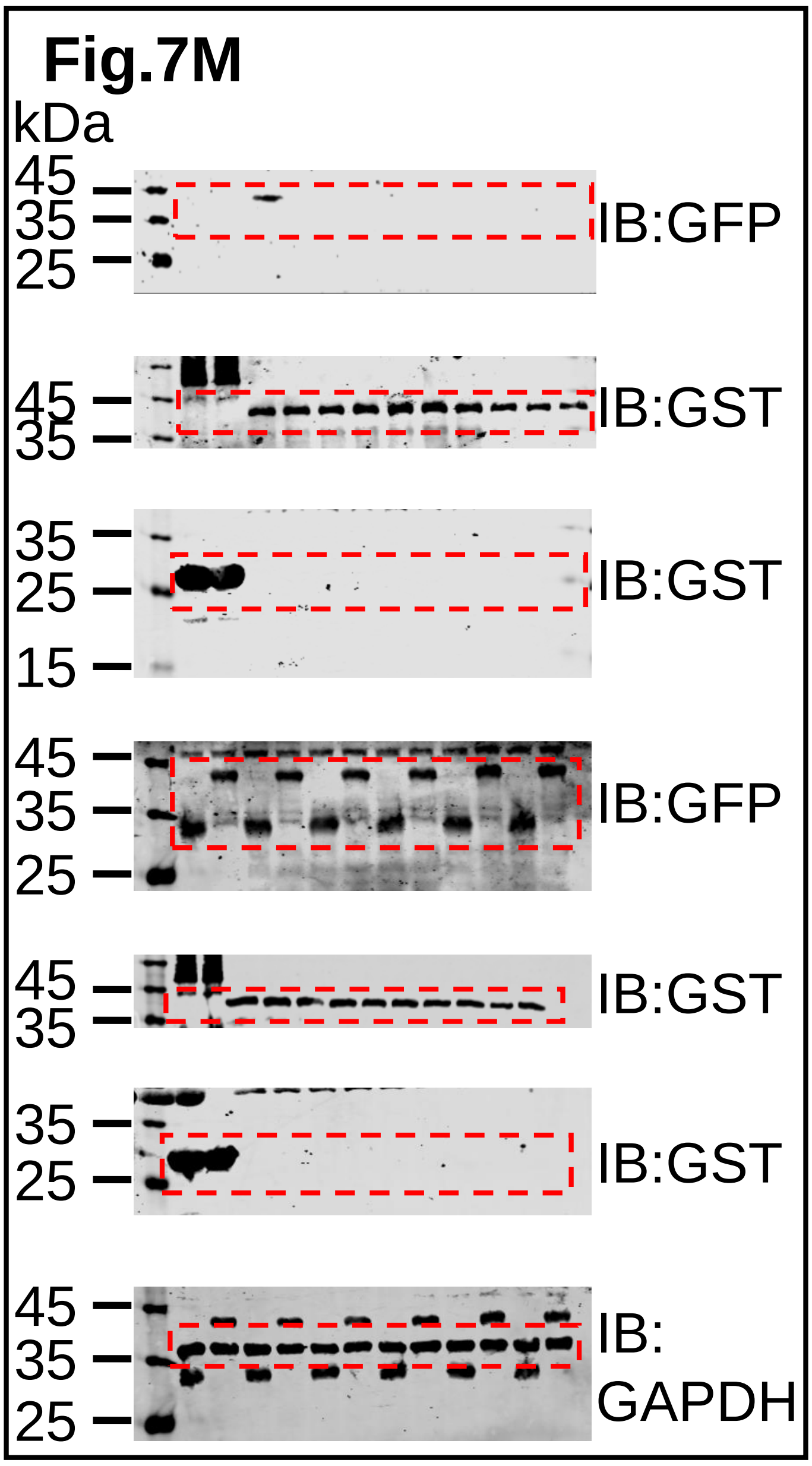
Fig.7M
kDa
45
35
25
IB:GFP
45
35
IB:GST
35
25
15
IB:GST
45
35
25
IB:GFP
45
35
IB:GST
35
25
IB:GST
45
35
25
IB:
GAPDH

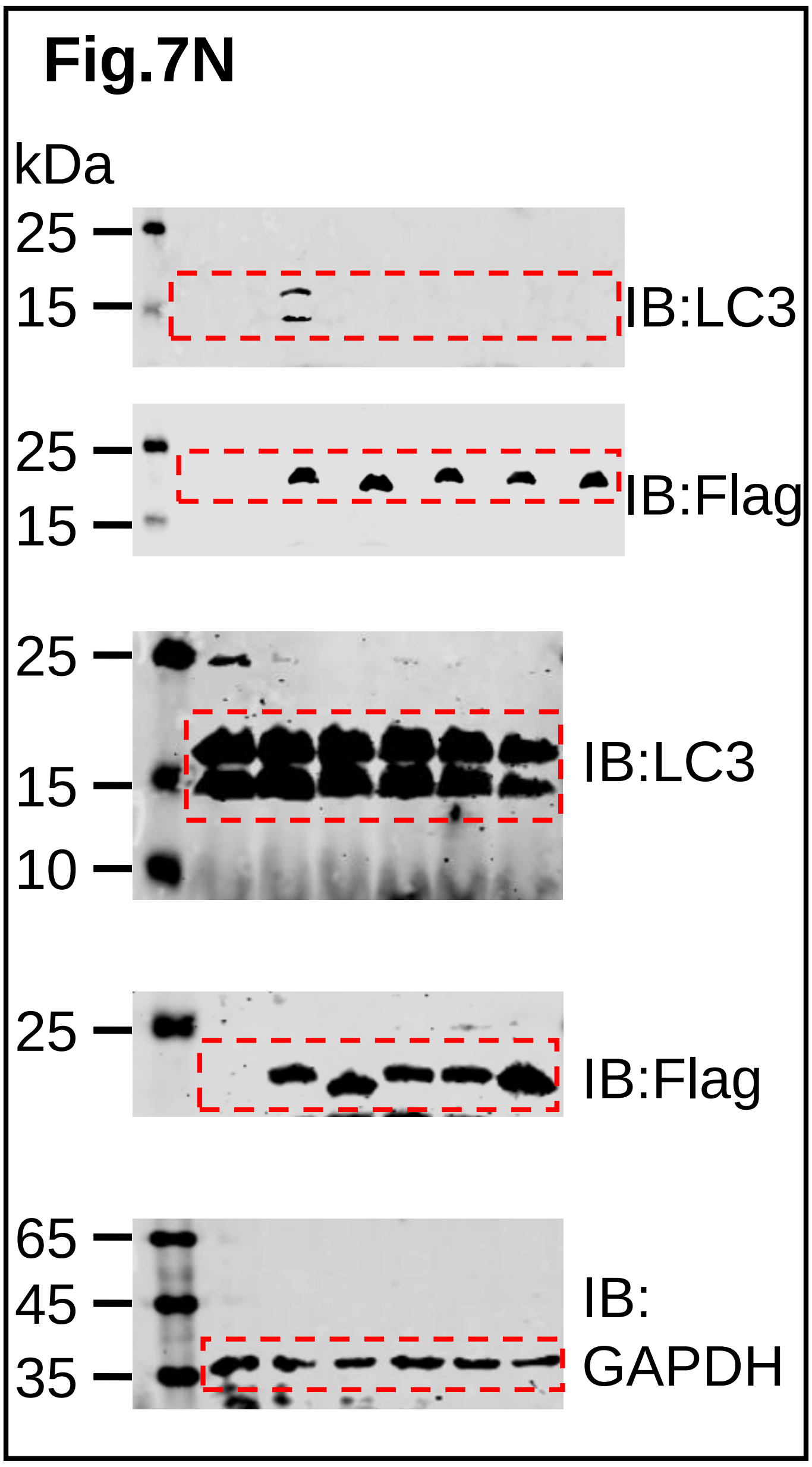
Fig.7N
kDa
25
15
IB:LC3
25
15
IB:Flag
25
15
10
IB:LC3
25
IB:Flag
65
45
35
IB:
GAPDH

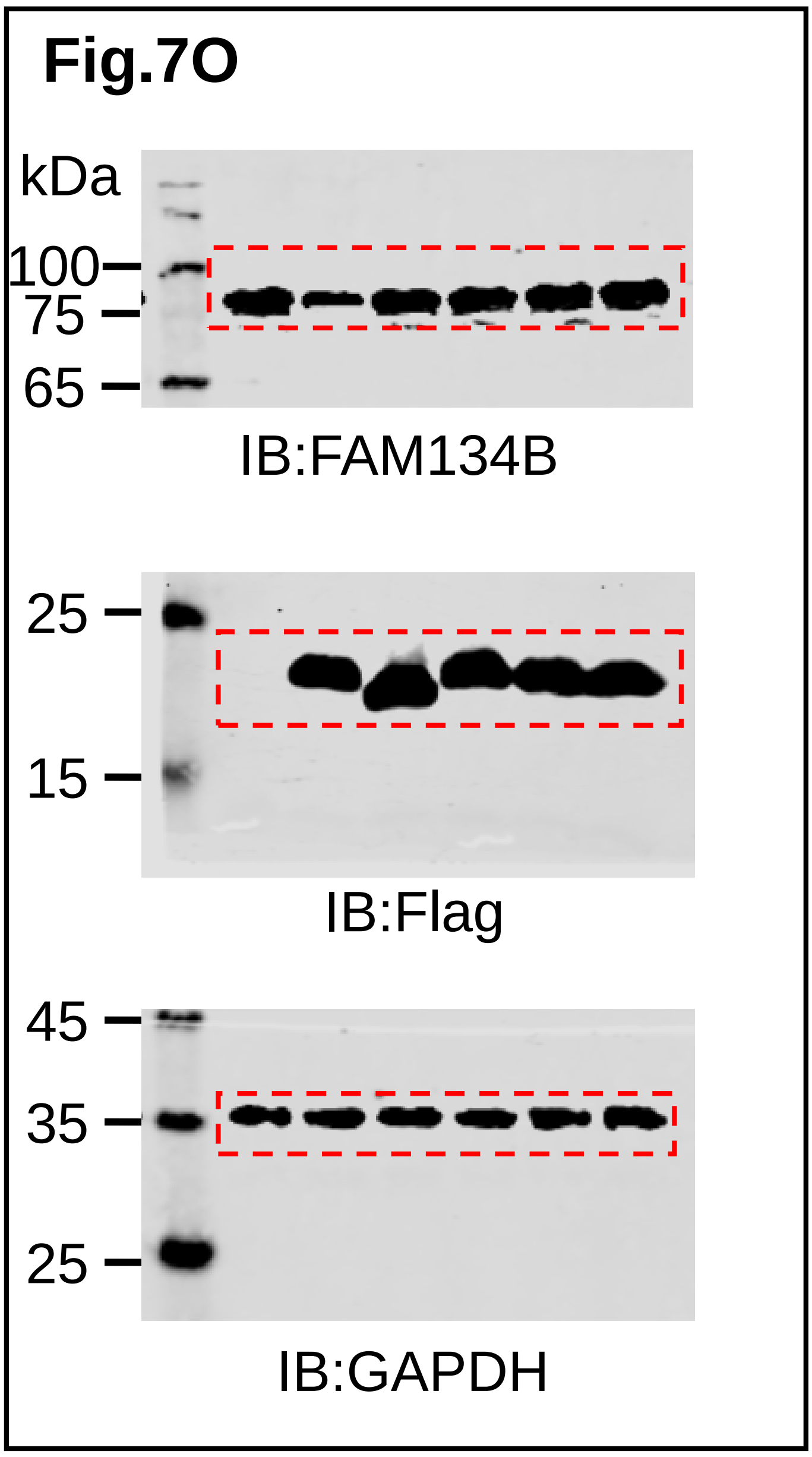
Fig.7O
kDa
100
75
65
IB:FAM134B
25
15
IB:Flag
45
35
25
IB:GAPDH

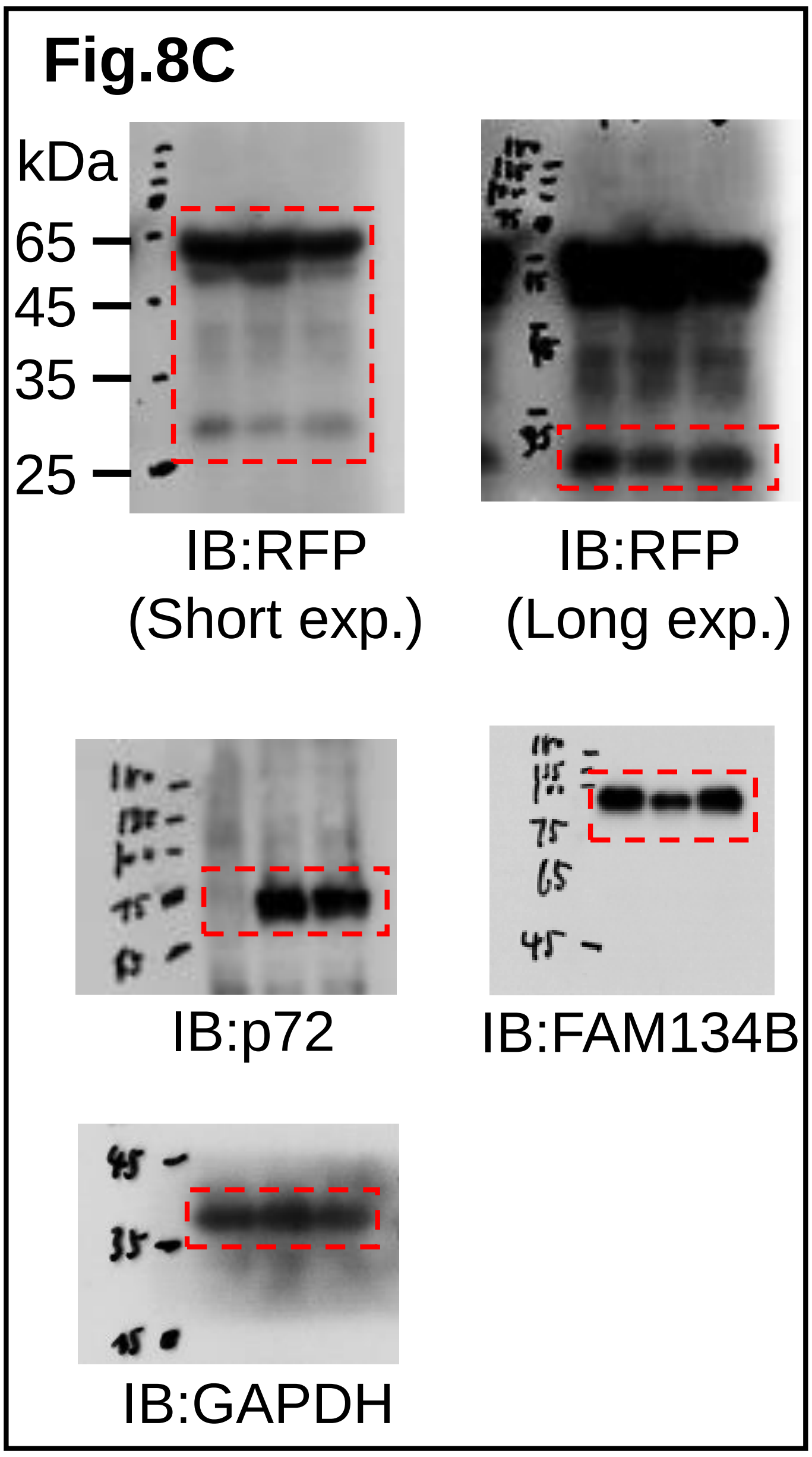
Fig.8C
kDa
65
45
35
25
IB:RFP
(Short exp.)
IB:RFP
(Long exp.)
IB:p72
IB:FAM134B
IB:GAPDH

Fig.8D

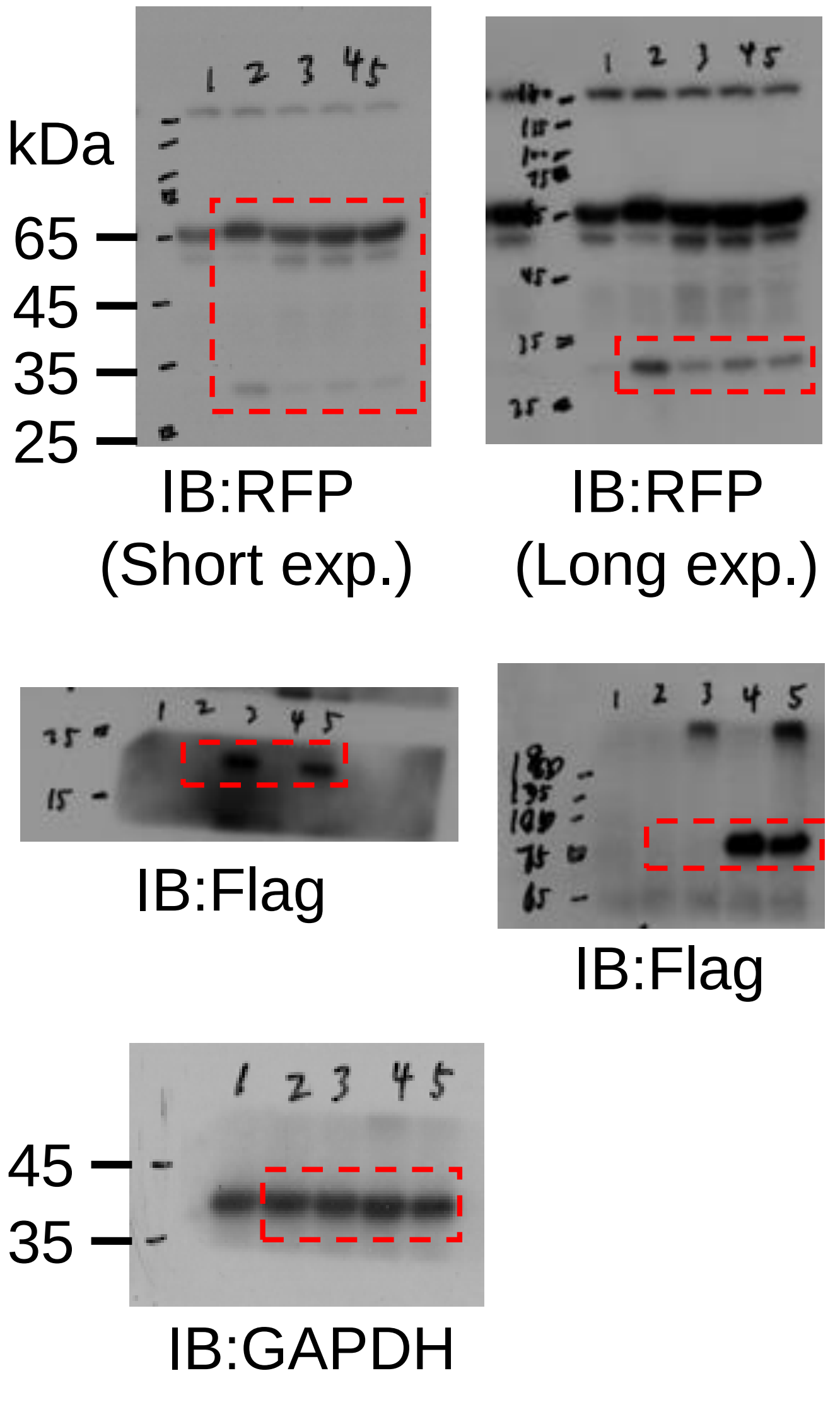

Fig.8E

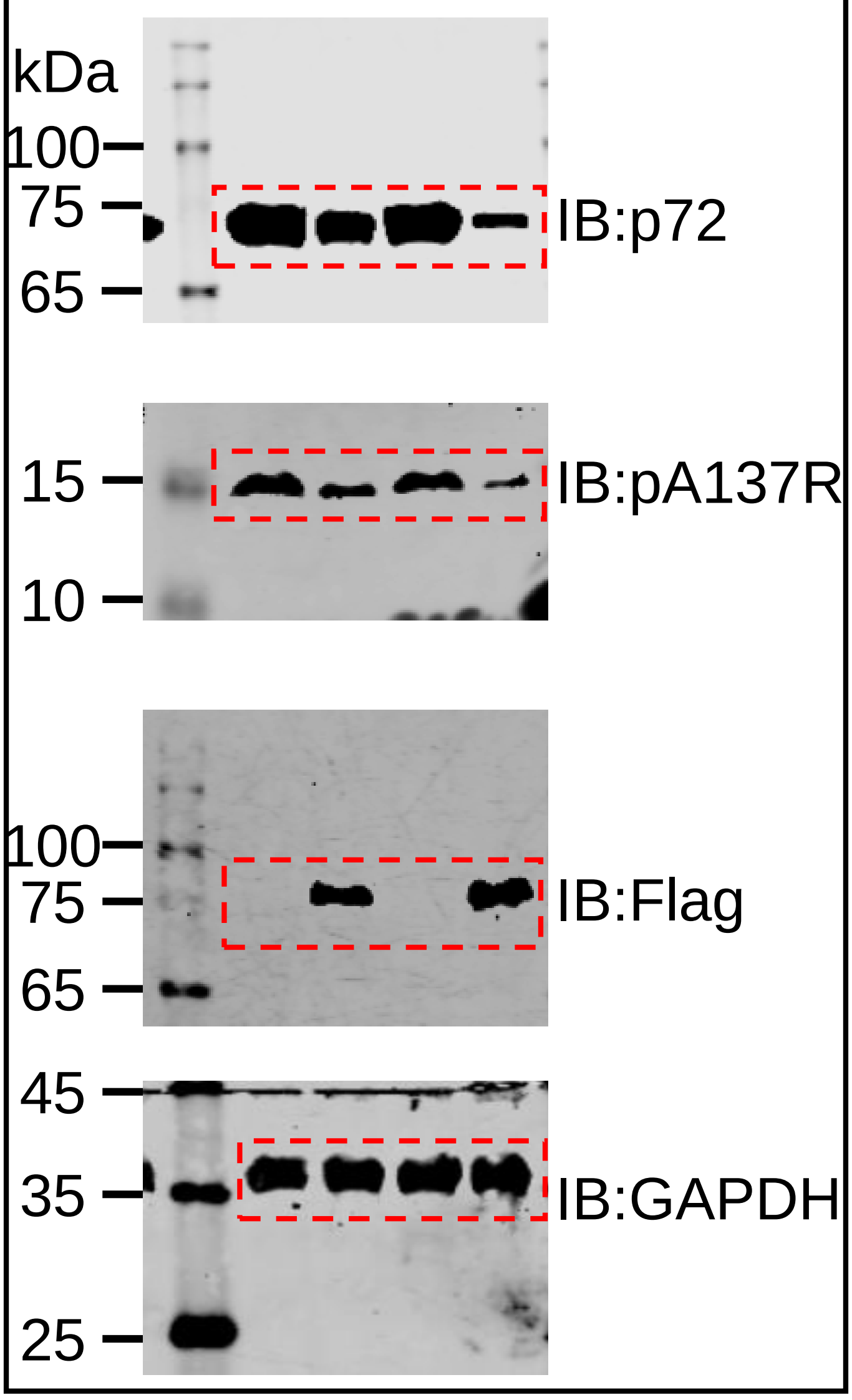

Fig.9D

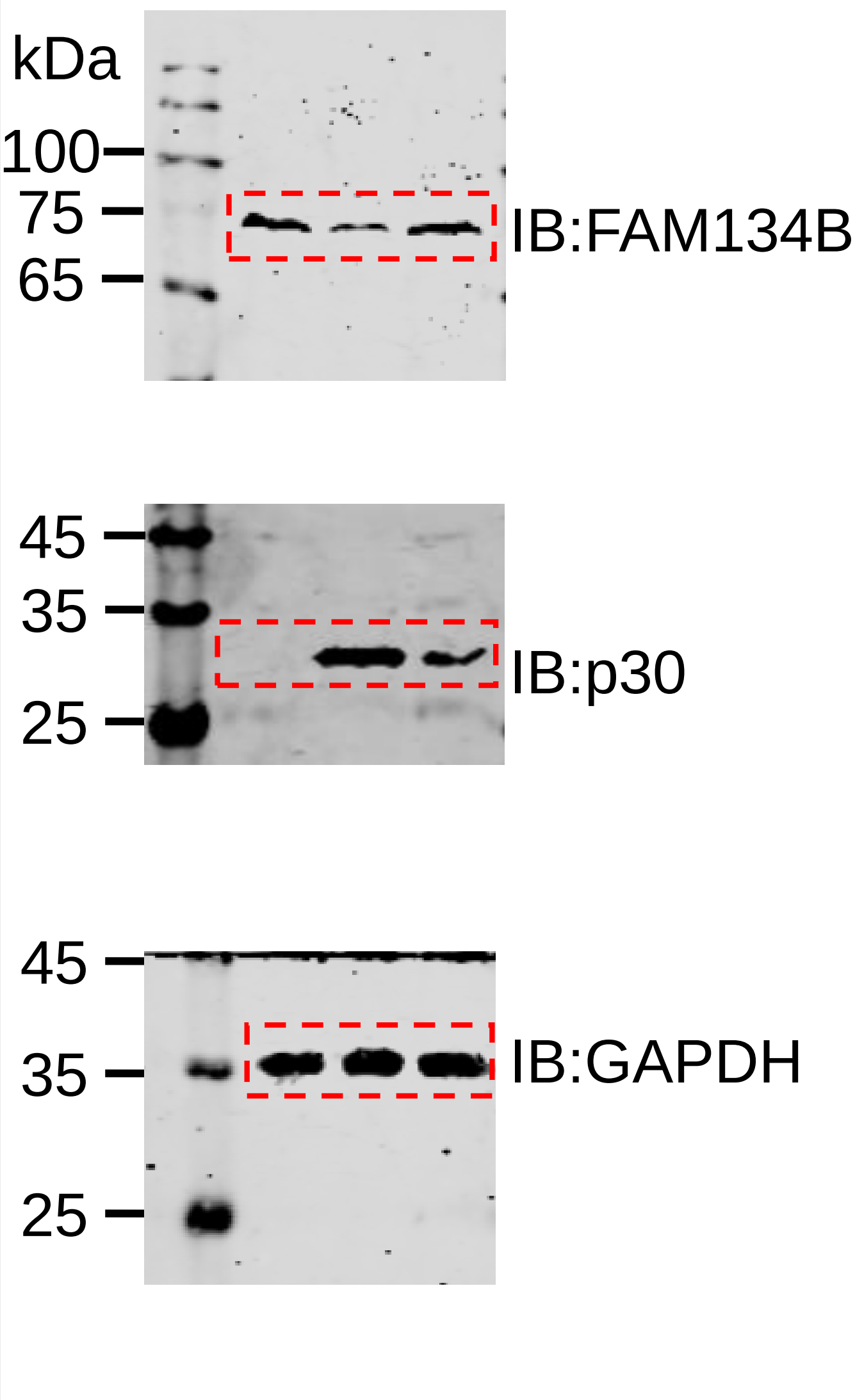

Fig.9I

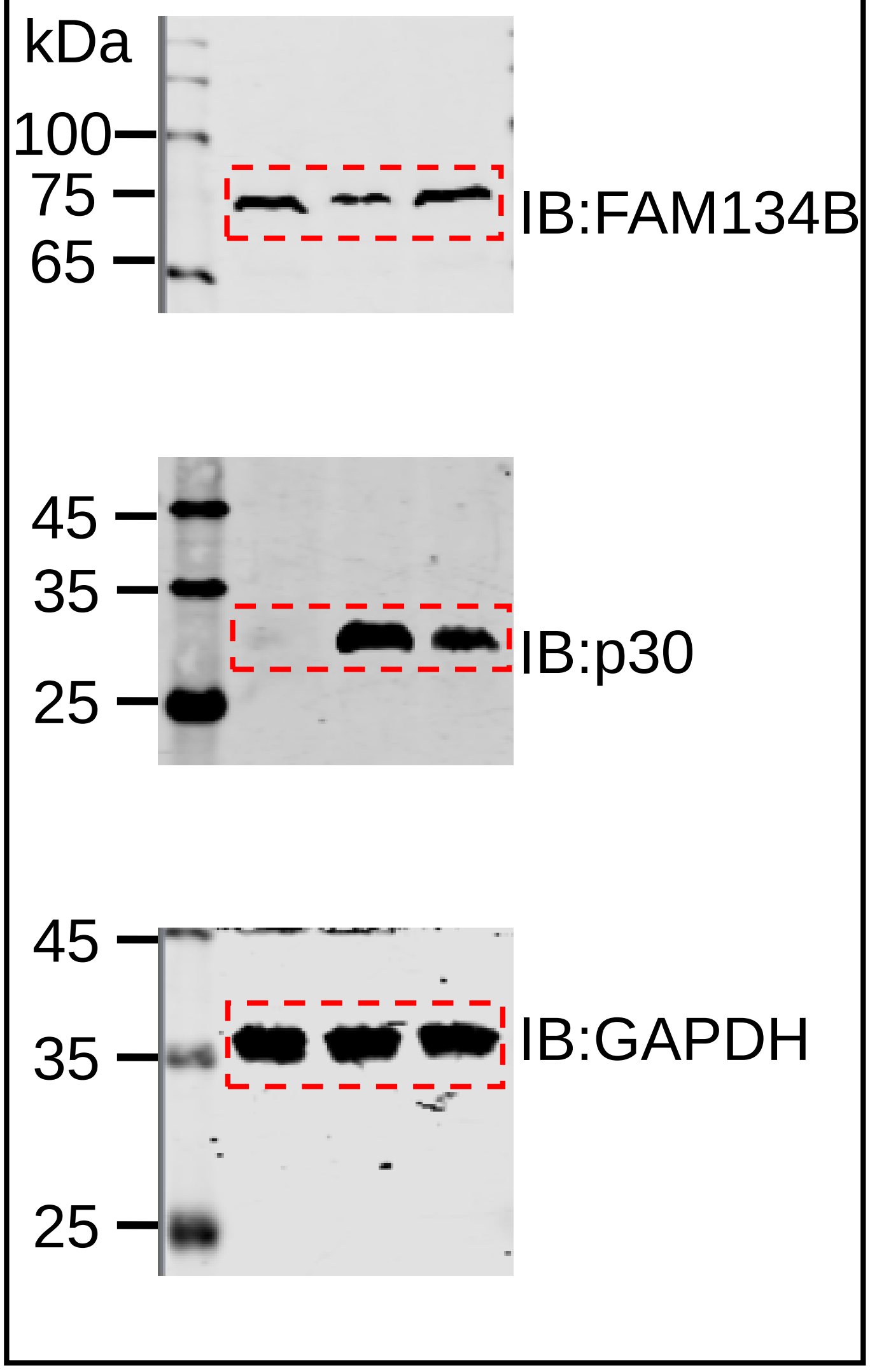

Fig.S3C

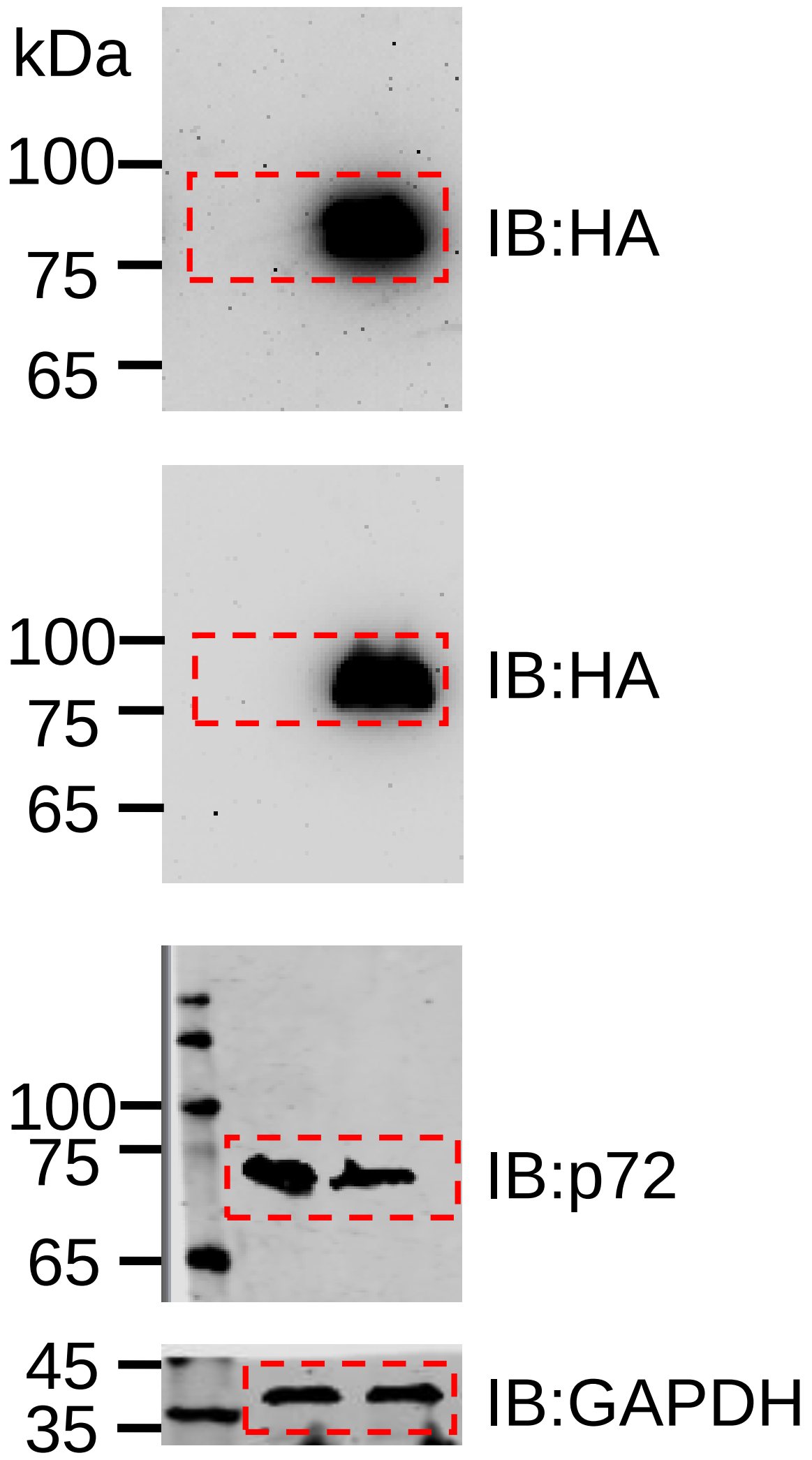

Fig.S4A

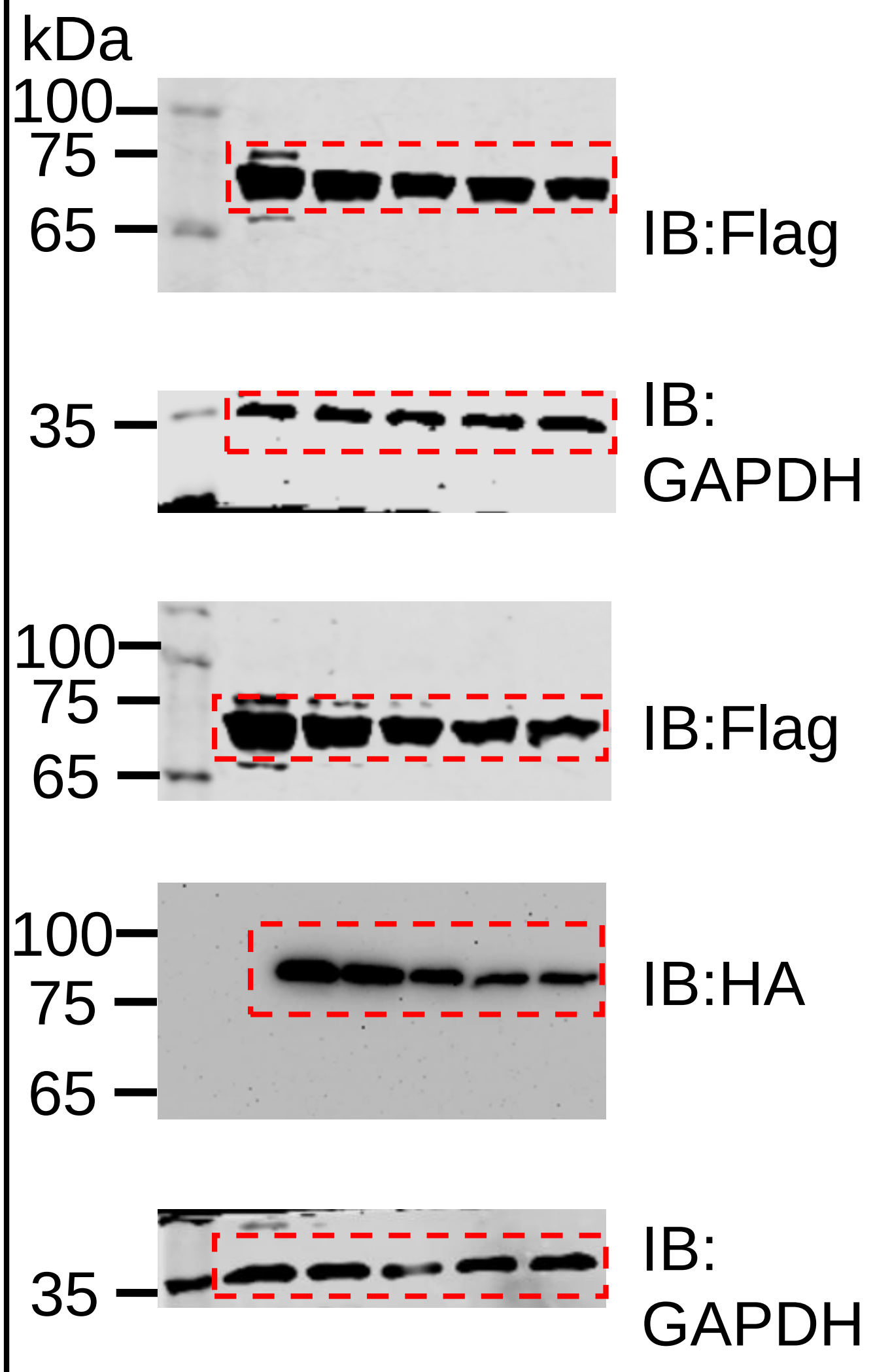

Fig.S4B

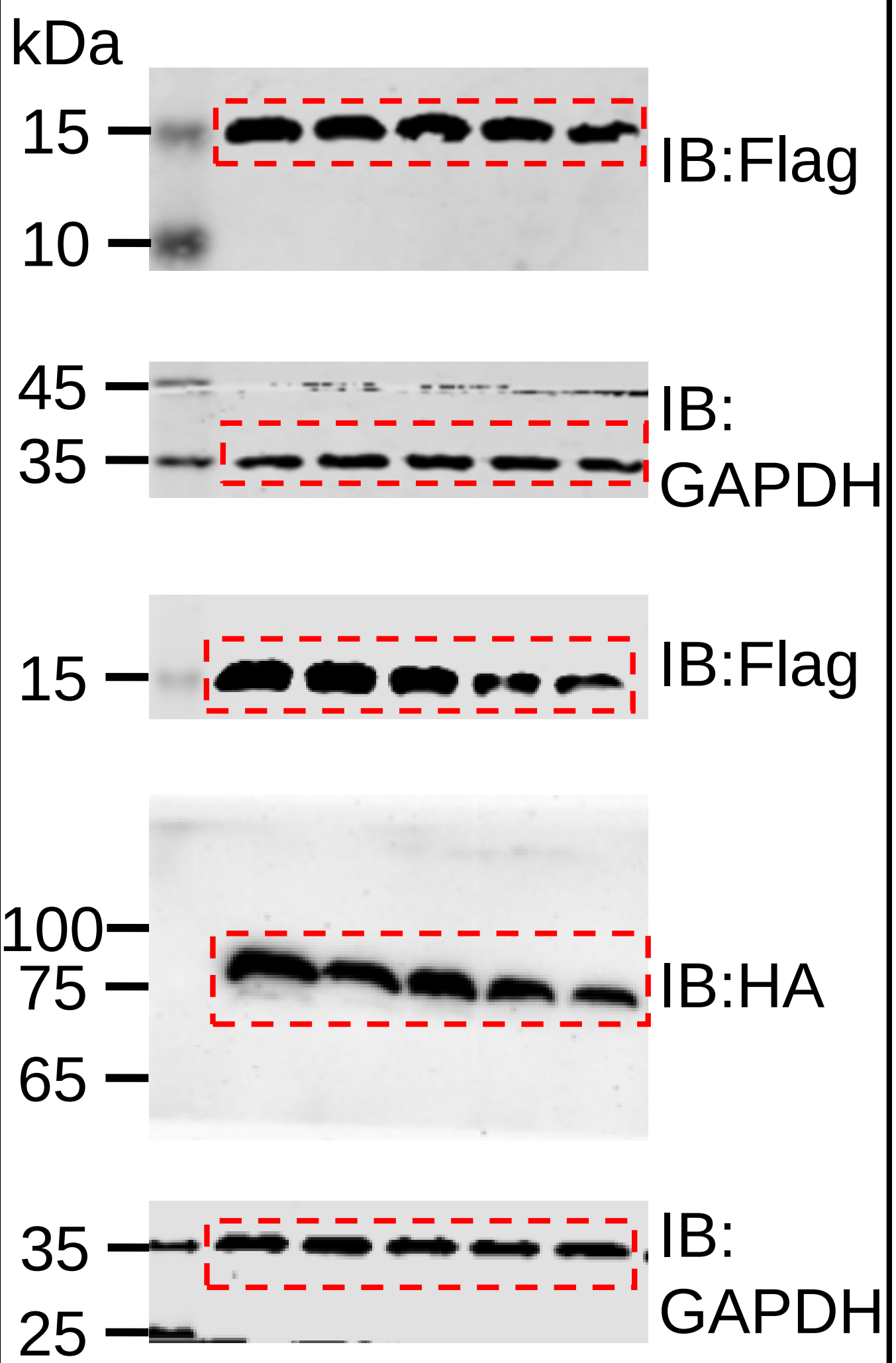

Fig.S4C

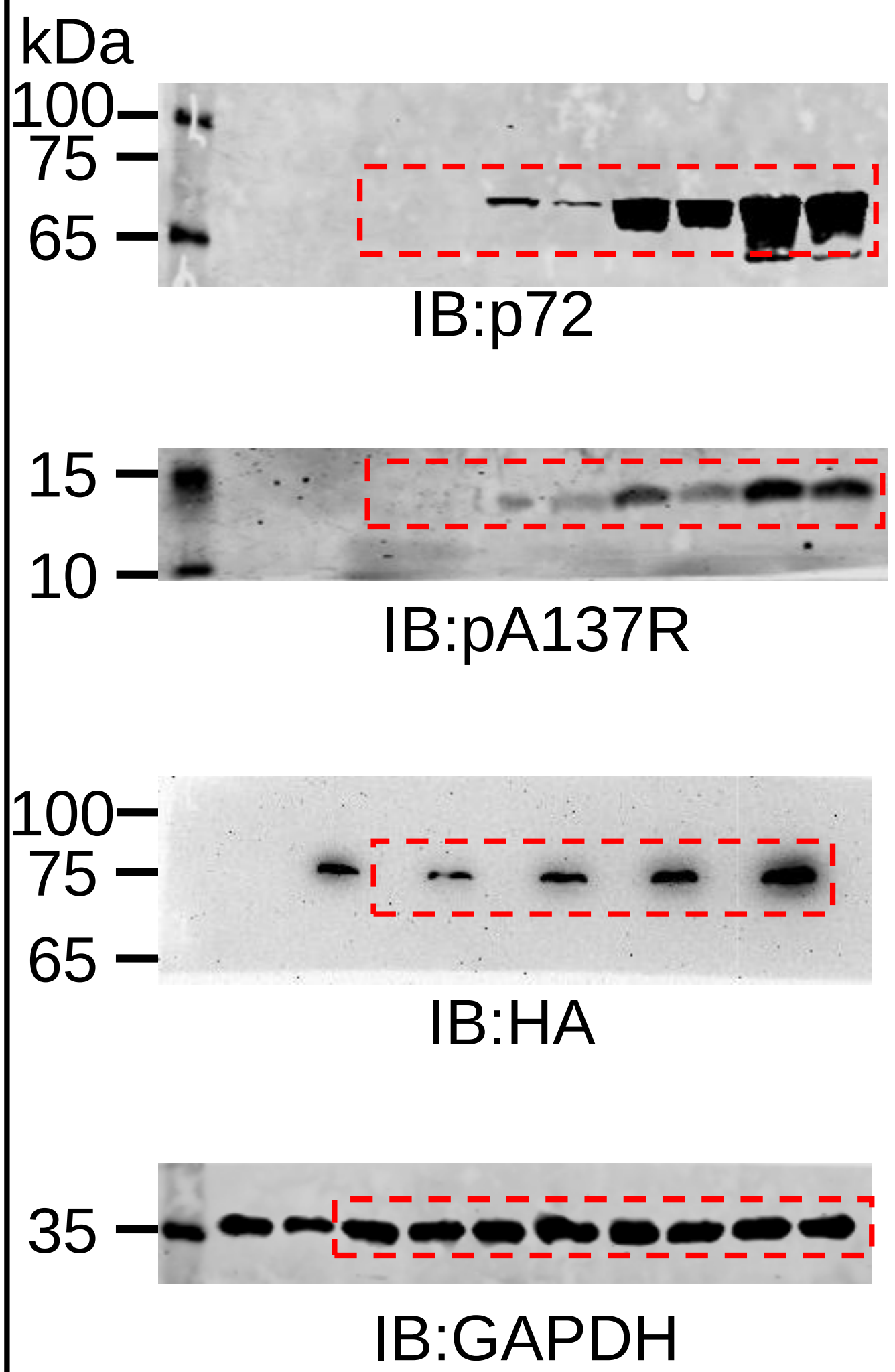

Fig.S5A

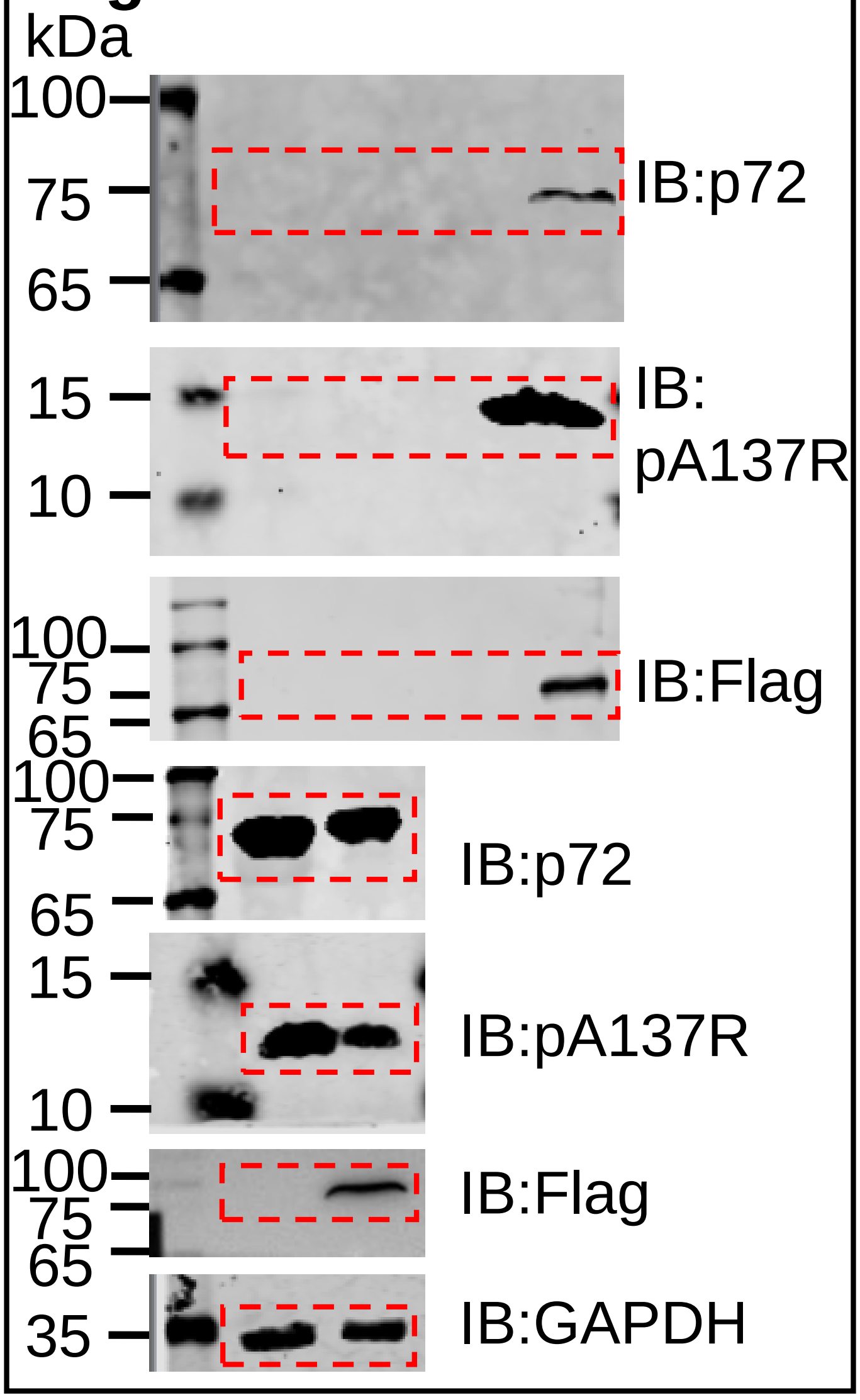

Fig.S5C

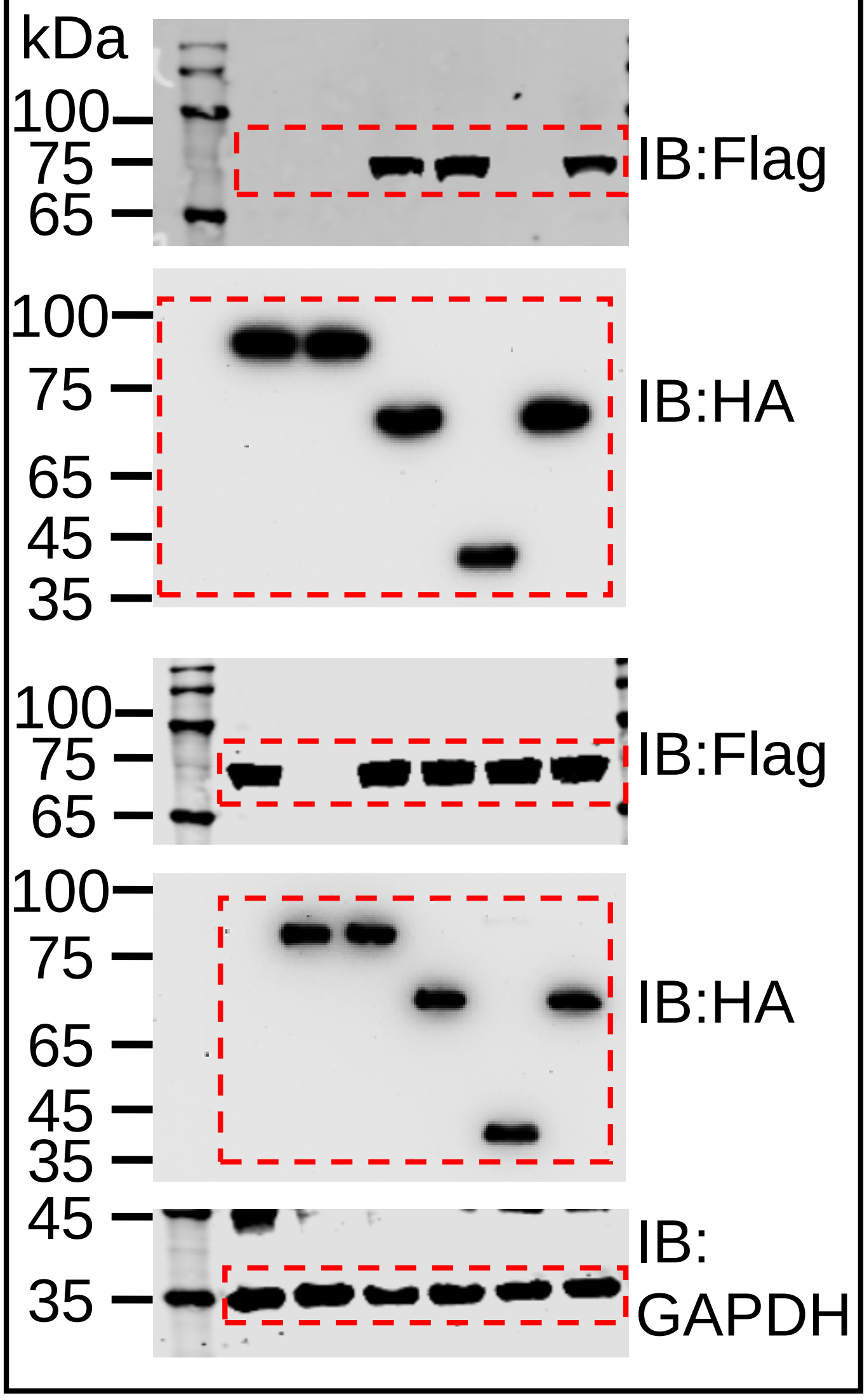

Fig.S5D

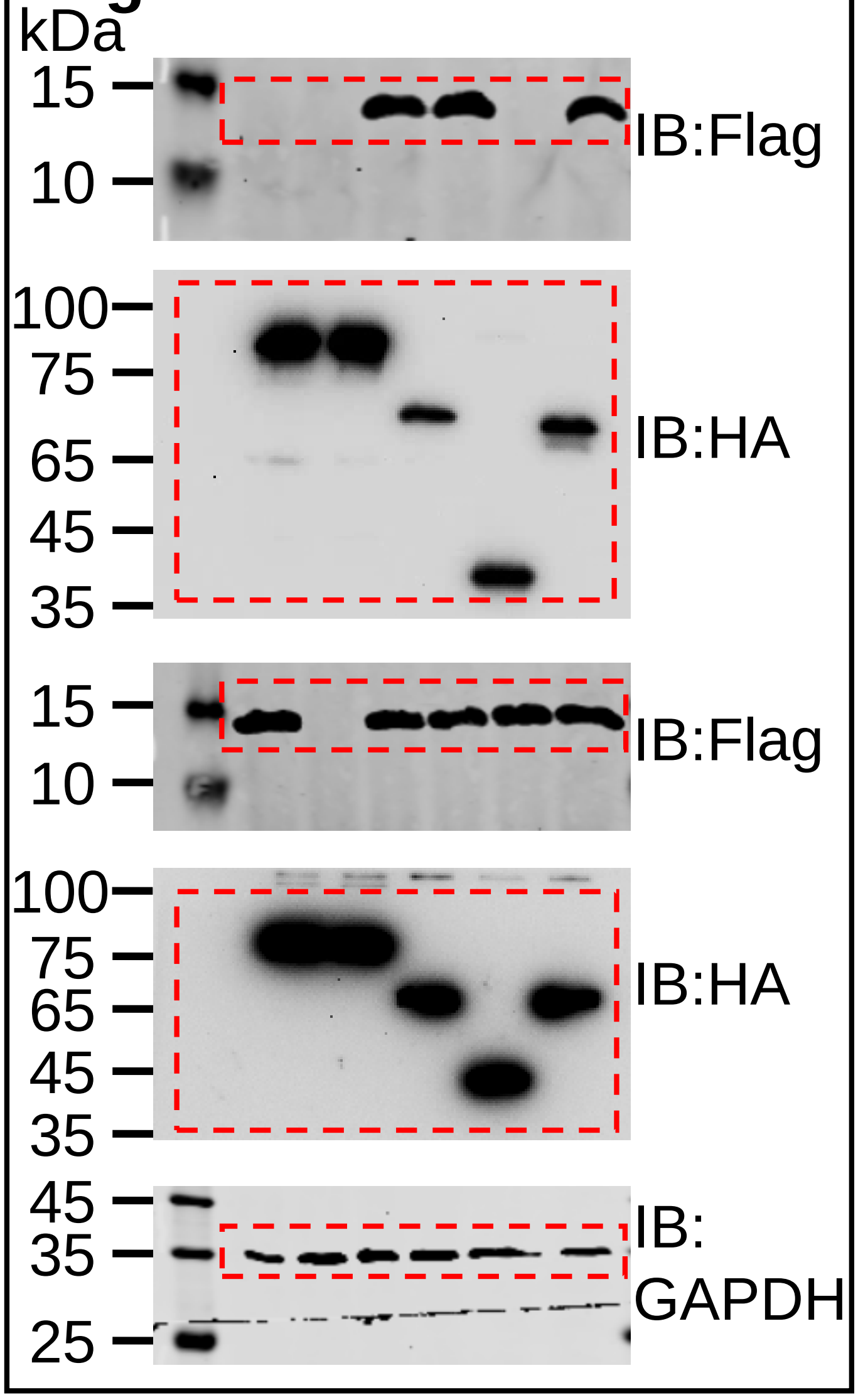

Fig.S5E

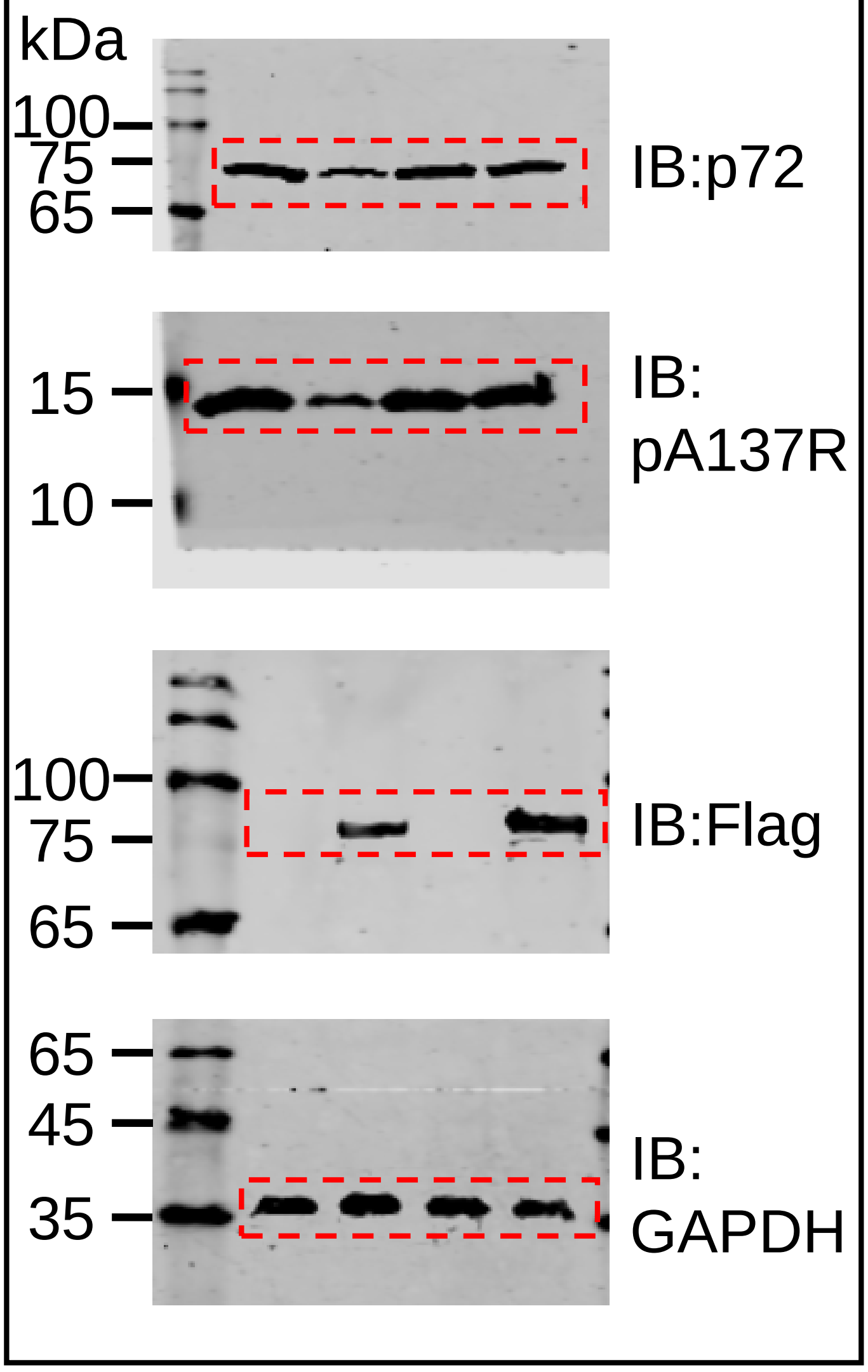

Fig.S6A

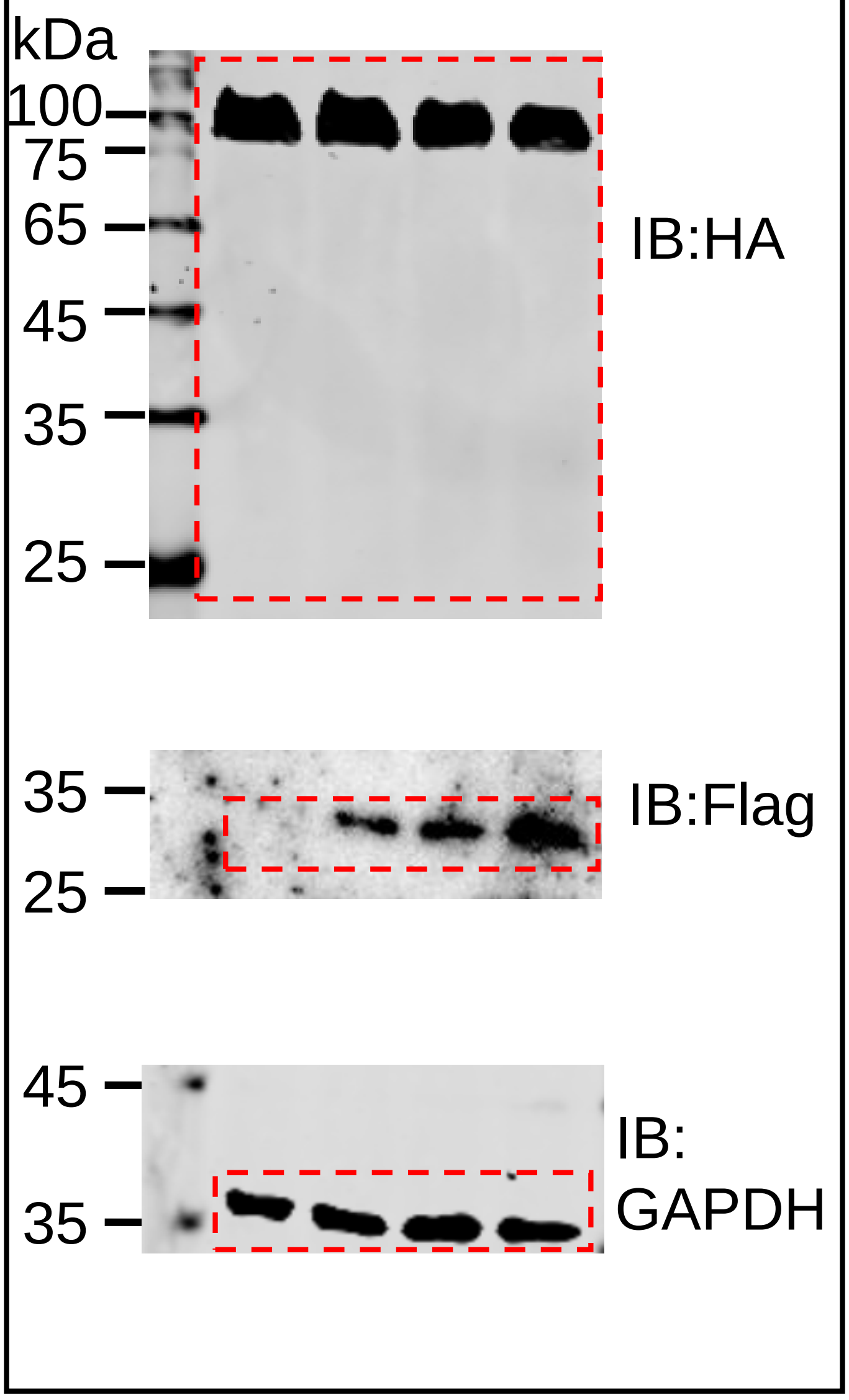

Fig.S6B

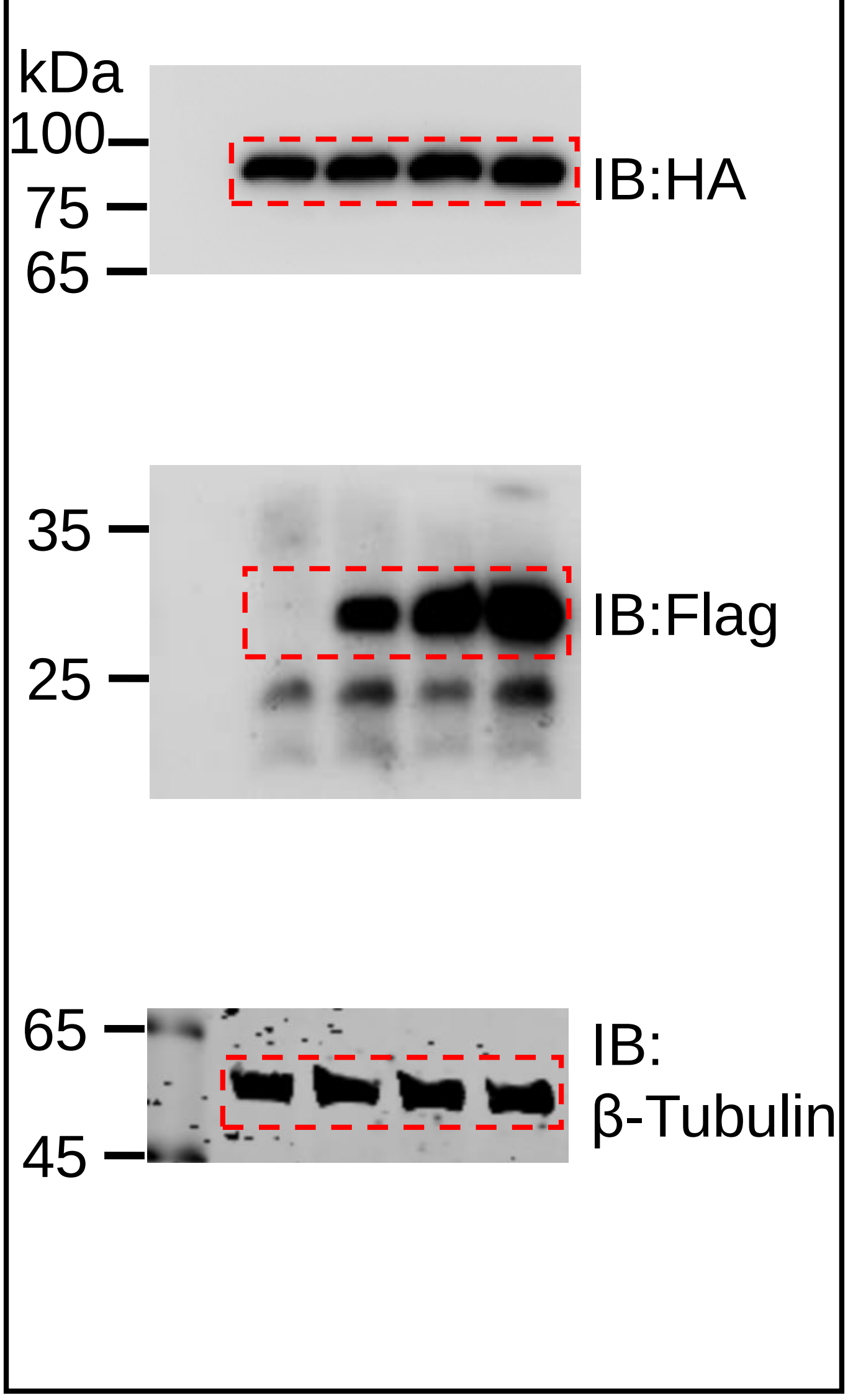

Fig.S6C

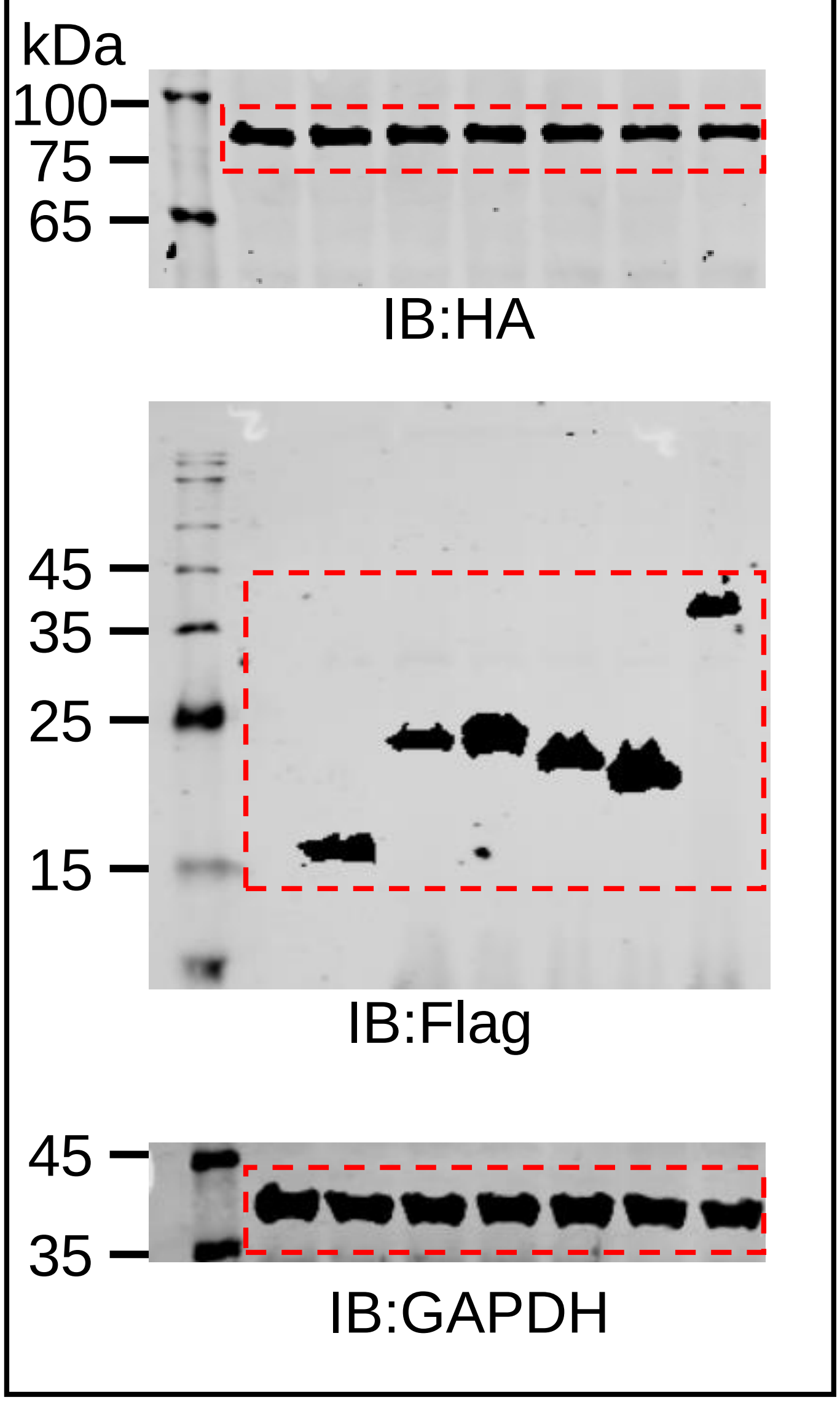

Fig.S6D

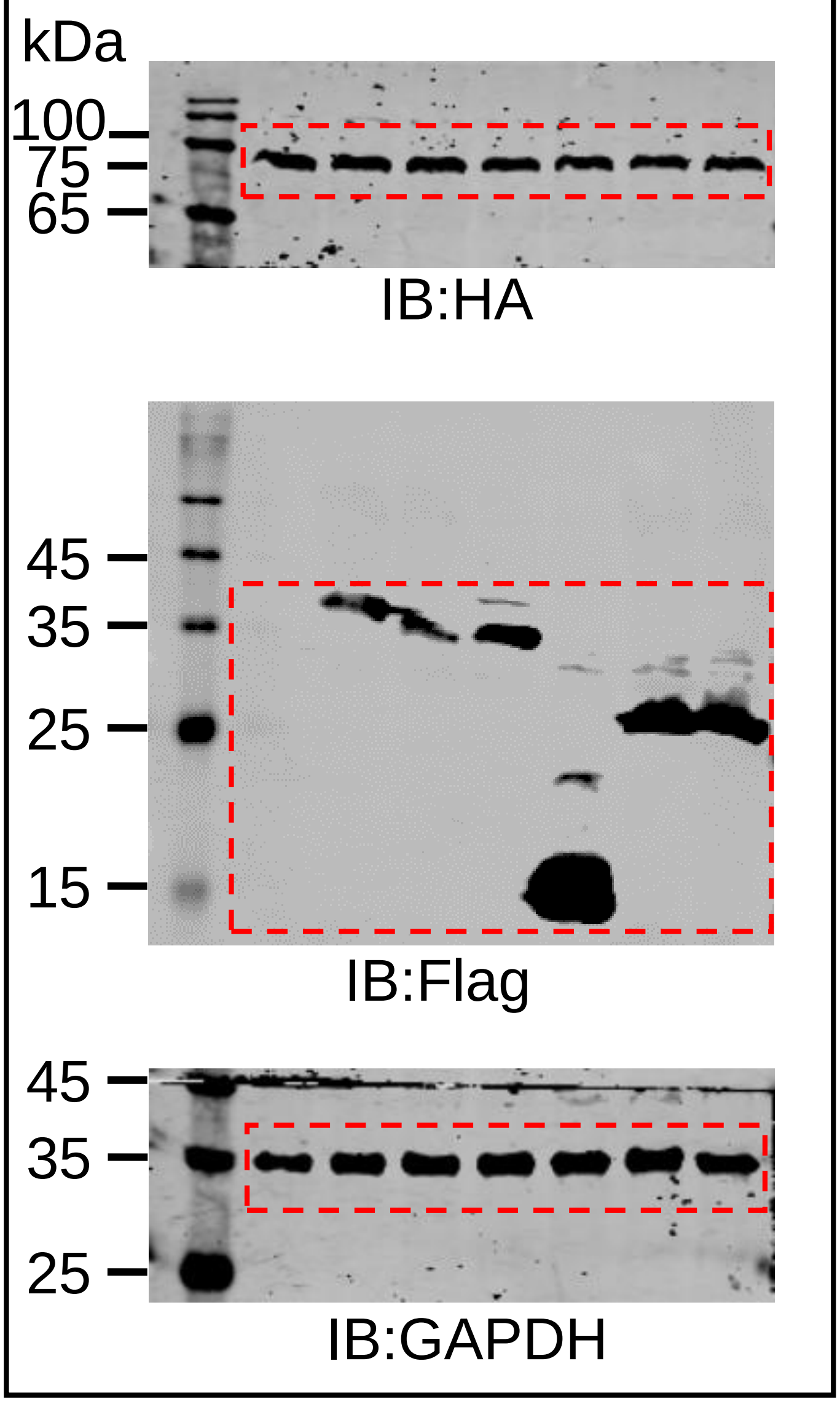

Fig.S6E

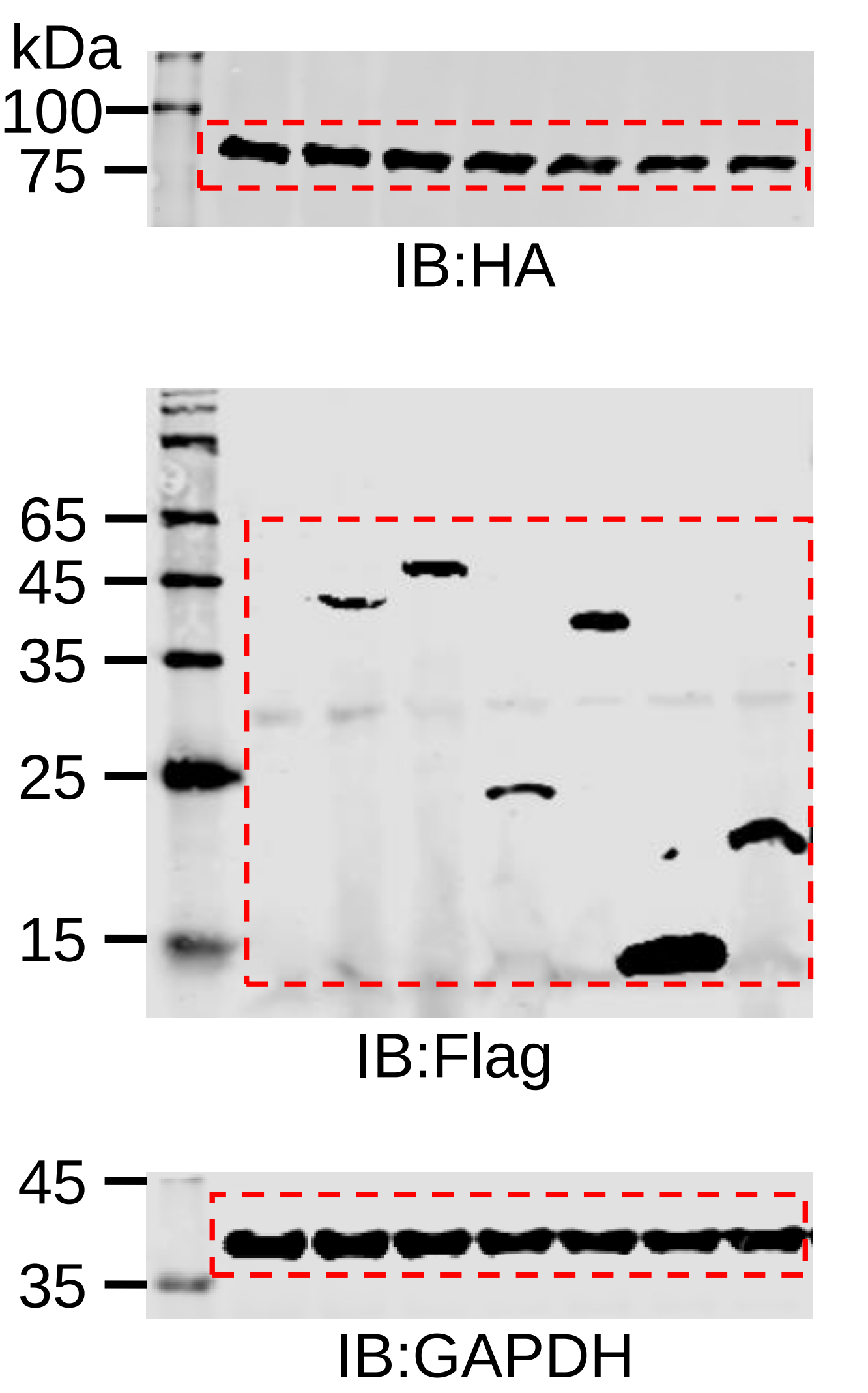

Fig.S6F

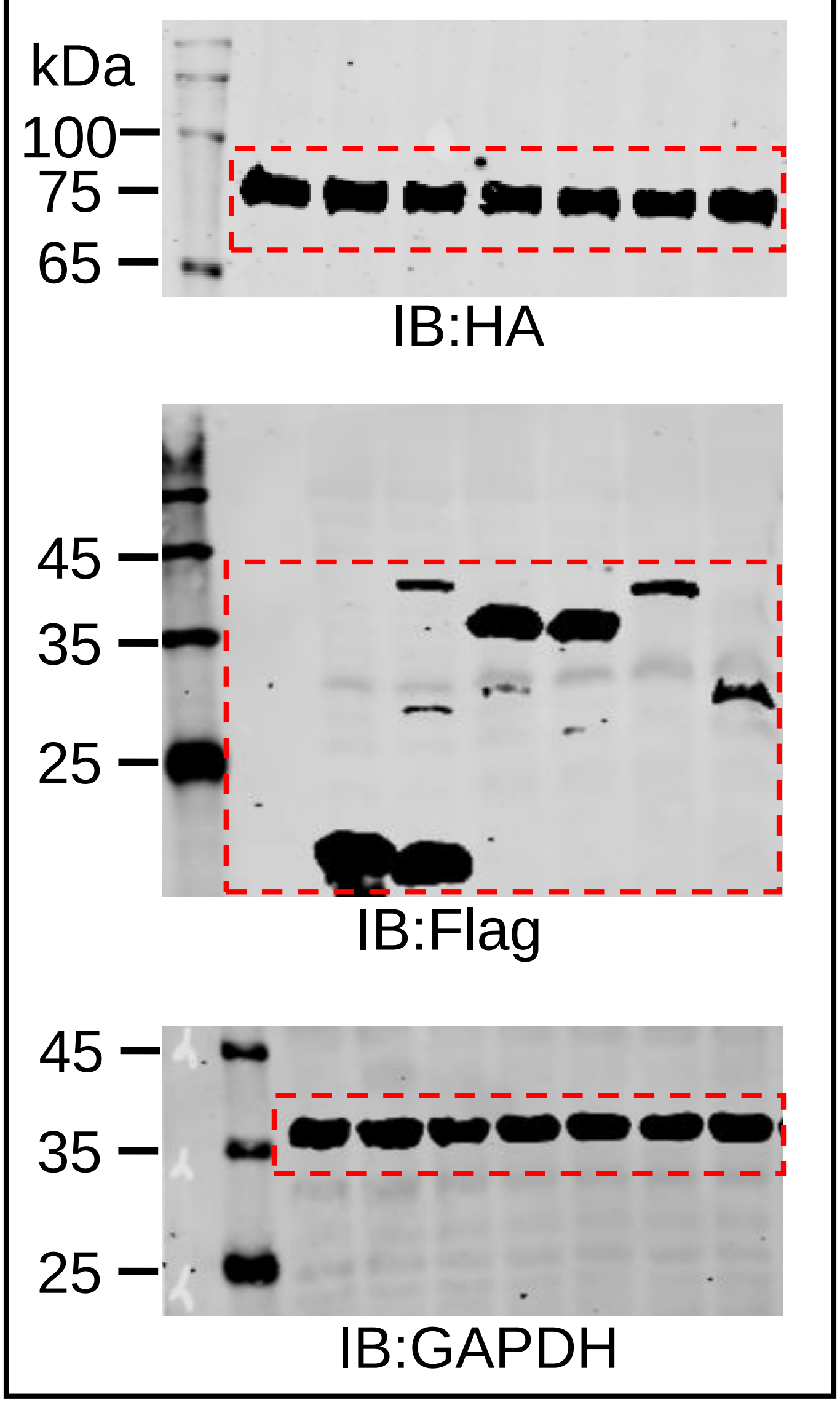

Fig.S6G

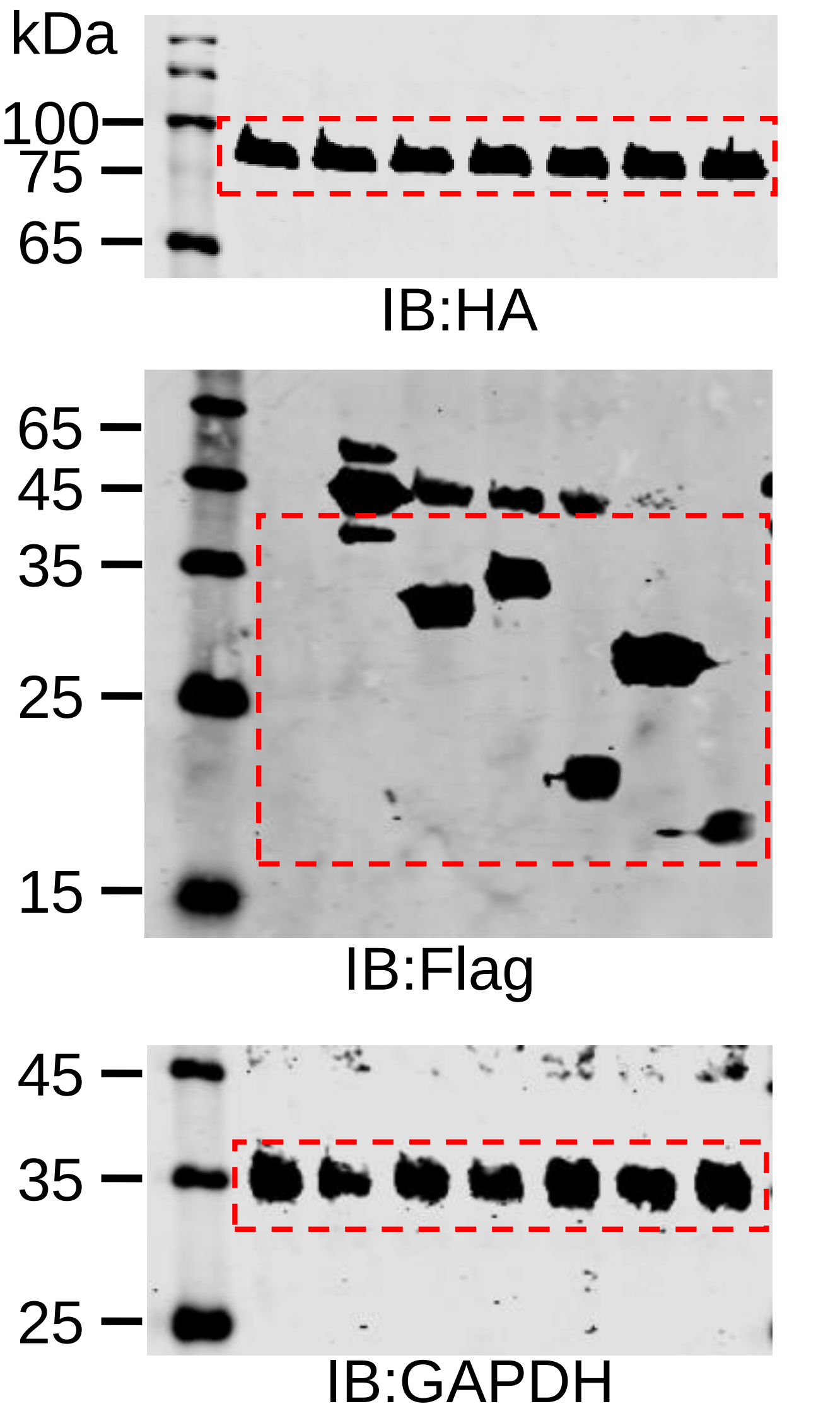

Fig.S6H

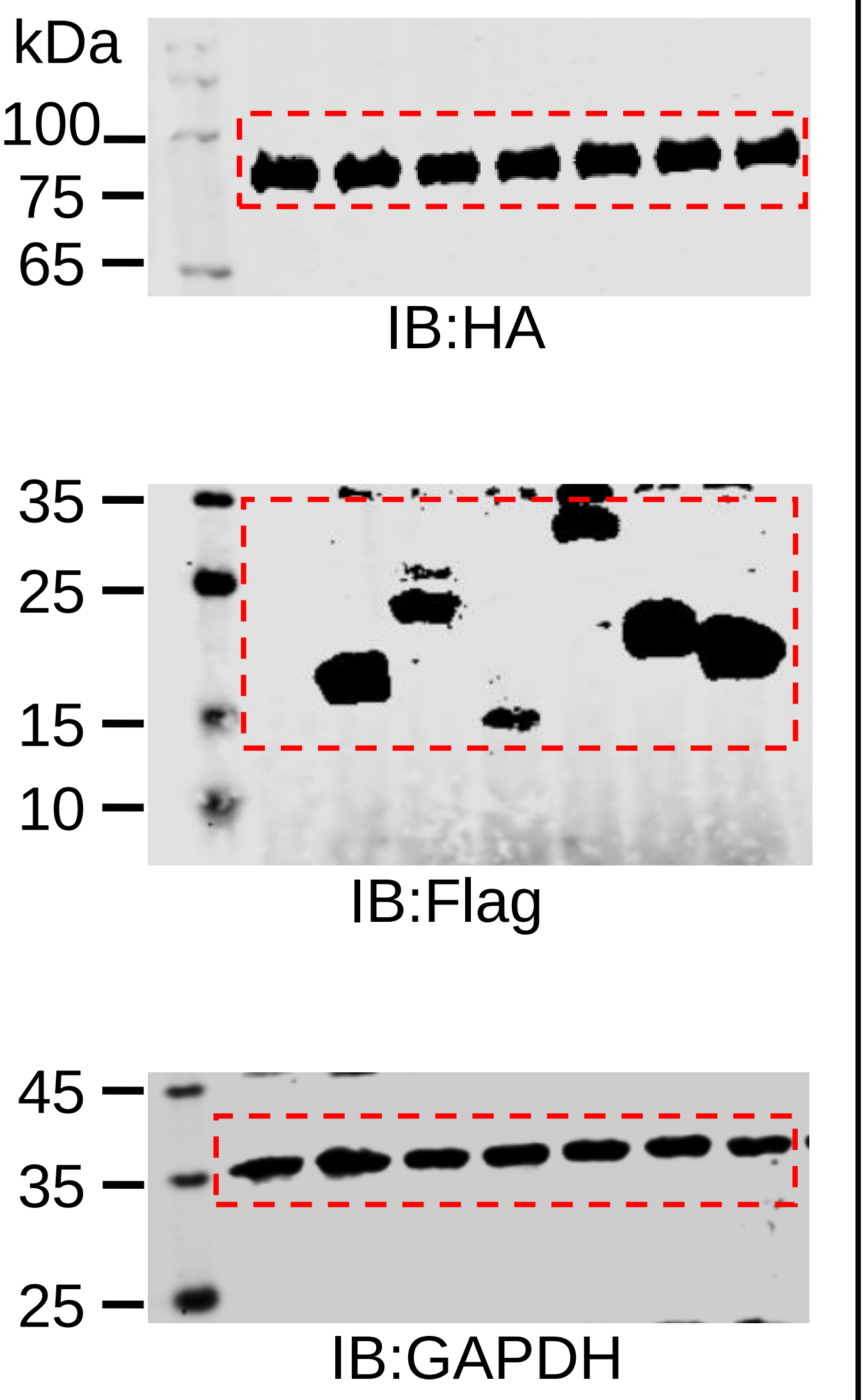

Fig.S6I

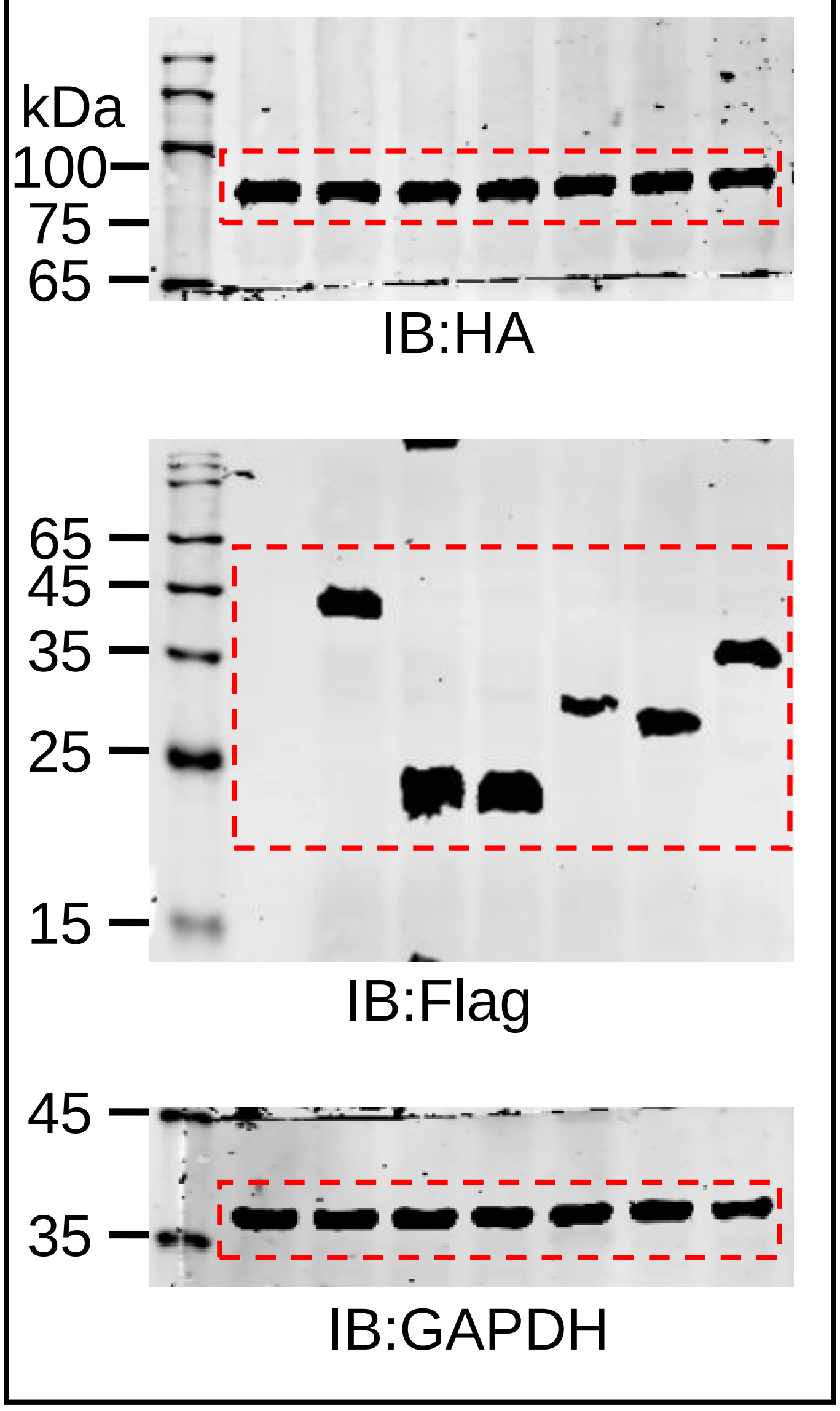

Fig.S6J

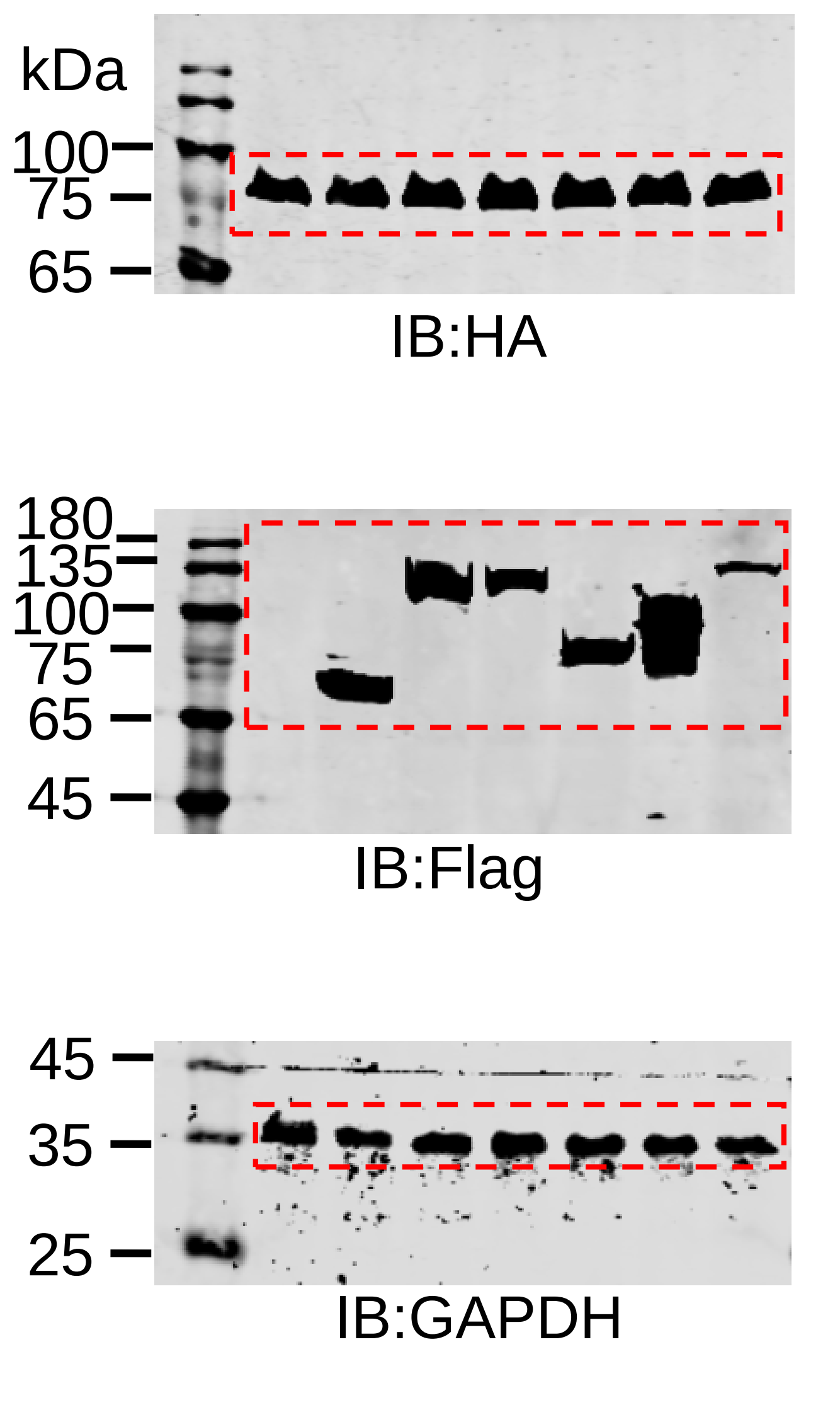

Fig.S6K

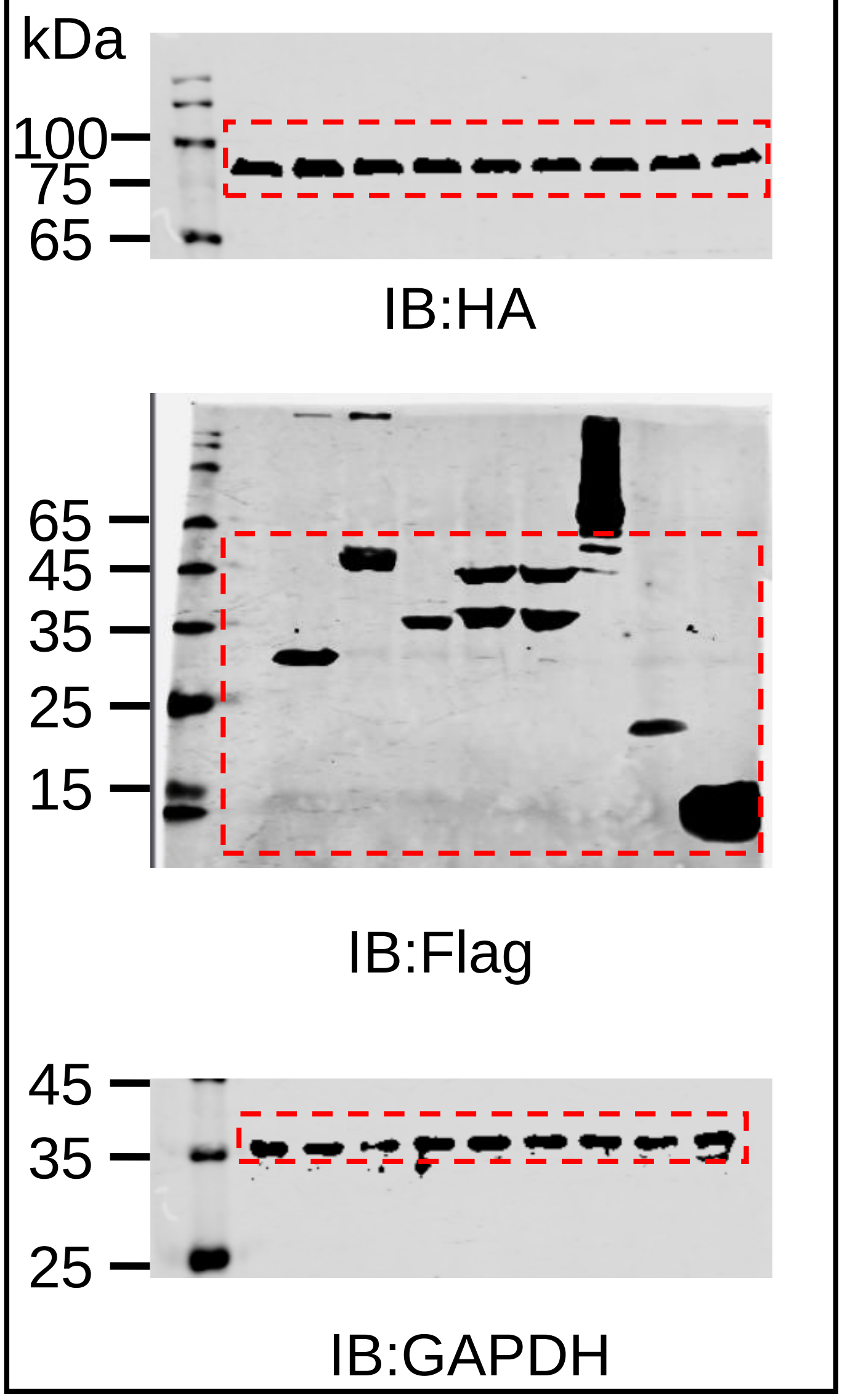

Fig.S7A

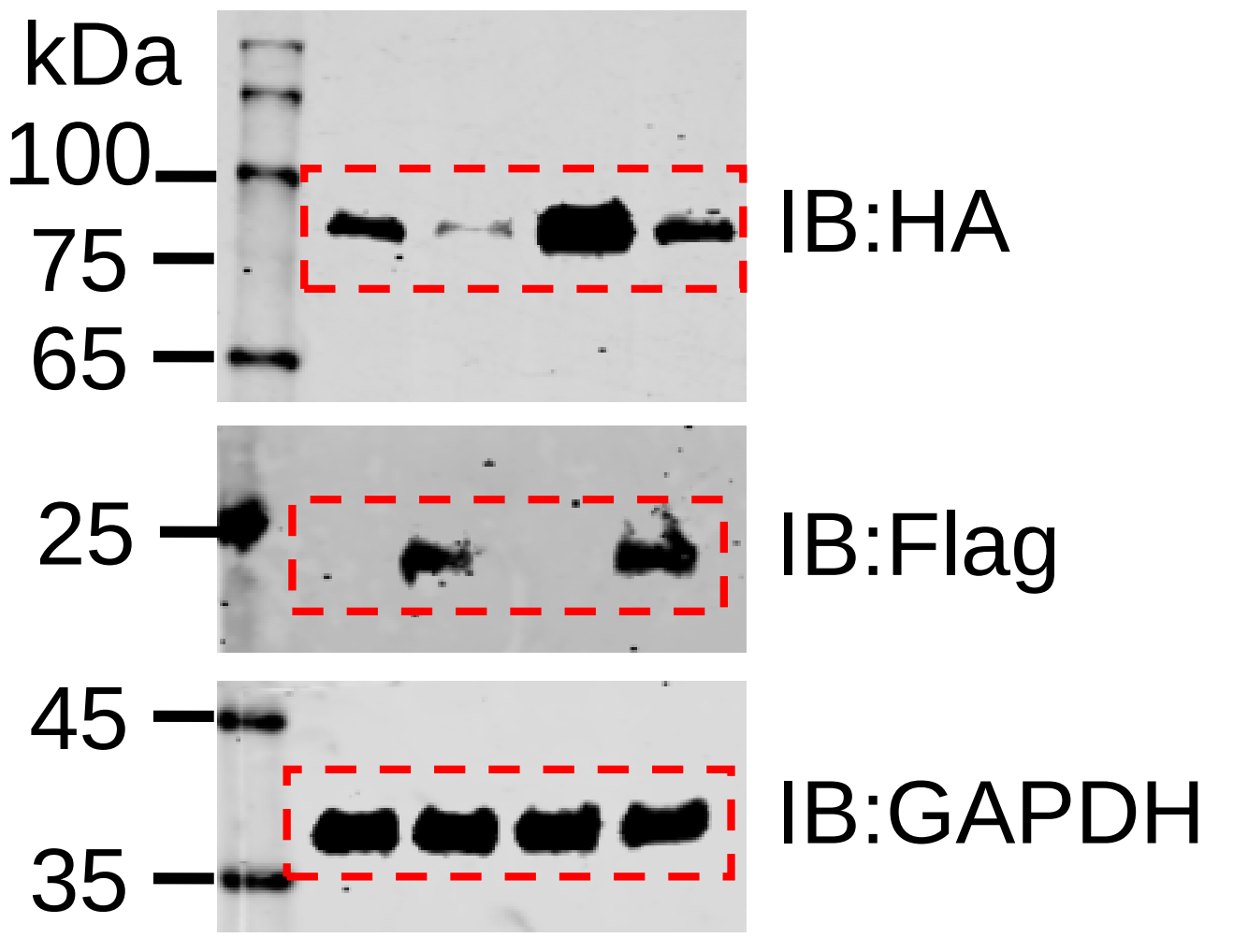

Fig.S7B

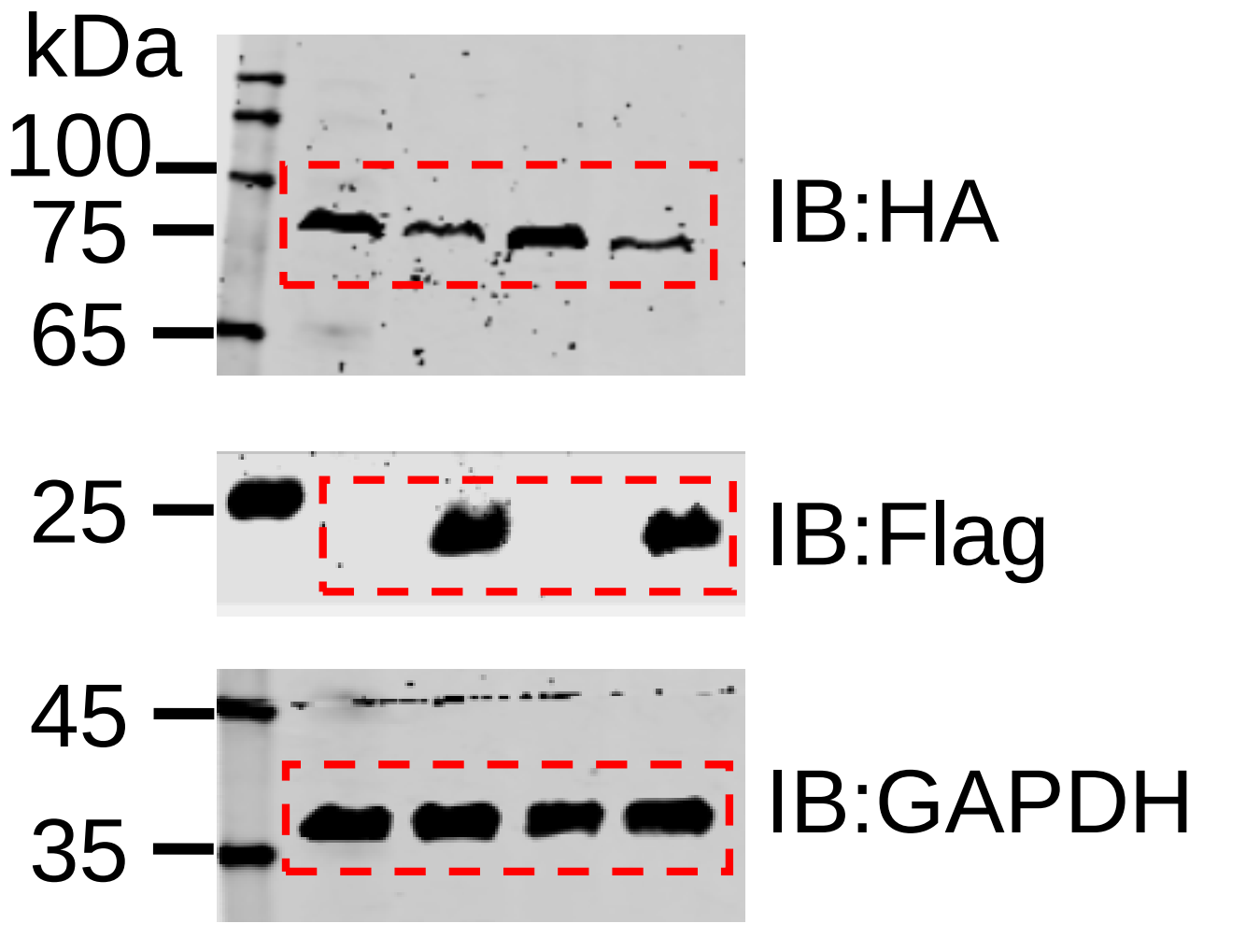

Supplement: Supplementary file 1 [file ADVS-13-e77741-s001.pdf]
